# Spontaneous embryo resorption in the mouse is triggered by embryonic apoptosis followed by rapid removal via maternal sterile purulent inflammation

**DOI:** 10.1186/s12861-019-0201-0

**Published:** 2020-01-09

**Authors:** Barbara Drews, Luis Flores Landaverde, Anja Kühl, Ulrich Drews

**Affiliations:** 1 Group Animal Physiology, Institute of Agricultural Sciences, Departement Environmental System Science, Swiss Federal Institue of Technology (ETH),, Zurich, Switzerland; 2 Group Reproduction Management, Institute of Zoo- and Wildlife Medicine (IZW), Berlin, Germany; 3 Core Unit Immunopathology (ipath), Charité, Berlin, Germany; 4 Institute of Anatomy, Prof. em., University of Tubingen, Tubingen, Germany

**Keywords:** Trophoblast, Decidua, Pregnancy failure, Foam cells, Cell death, Macrophage

## Abstract

**Background:**

In normal mammalian development a high percentage of implantations is lost by spontaneous resorption. This is a major problem in assisted reproduction and blastocyst transfer. Which embryo will be resorbed is unpredictable. Resorption is very fast, so that with conventional methods only final haemorrhagic stages are encountered.

Here we describe the histology and immunohistochemistry of 23 spontaneous embryo resorptions between days 7 and 13 of murine development, which were identified by high-resolution ultrasound (US) in a previous study.

**Results:**

In the early resorptions detected at day 7, the embryo proper was replaced by maternal haemorrhage and a suppurate focus of maternal neutrophils. In the decidua maternal macrophages transformed to foam cells and formed a second focus of tissue dissolution.

In the late resorptions detected at day 9, the embryo underwent apoptosis without involvement of maternal cells. The apoptotic embryonic cells expressed caspase 3 and embryonic blood cells developed a macrophage like phenotype. Subsequently, the wall of the embryonic vesicle ruptured and the apoptotic embryo was aborted into the uterine lumen. Abortion was initiated by degeneration of the embryonic lacunar trophoblast and dissolution of the maternal decidua capsularis via sterile inflammation and accompanied by maternal haemorrhage, invasion of the apoptotic embryo by maternal neutrophils, and contraction rings of the uterine muscle layers.

**Conclusions:**

We conclude that spontaneous resorption starts with endogenous apoptosis of the embryo without maternal contribution. After break down of the foetal-maternal border, the apoptotic embryo is invaded by maternal neutrophils, aborted into the uterine lumen, and rapidly resorbed. We assume that the innate maternal unspecific inflammation is elicited by disintegrating apoptotic embryonic cells.

**Graphical abstract:**

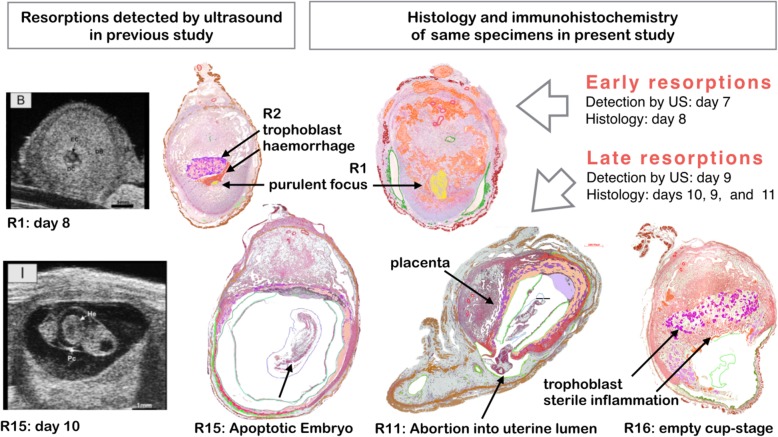

## Background

Spontaneous resorption designates loss of implantations and implies that maternal or embryonic causes are unknown. In normal development of mammals, a high percentage of blastocysts are lost before or after implantation by spontaneous resorption. In the human, Hertig [1] observed that about 70% of human implantation sites between day 6 (implantation) and day 14 *p.o.* were defective and thus prone to resorption. Spontaneous resorption in the human occurring within the second week of embryonic development is followed by normal menstruation and therefore goes unnoticed. The loss of the ovum before implantation under perfect conditions of reproduction, the “perfect failure” [1] and spontaneous resorption after implantation are evolutionary conserved reproduction strategies of mammals and accommodate the normal mutation rate leading to a high percentage of non-viable embryos during normal reproduction.

Assessment of the occurrence of spontaneous resorption in early stages of pregnancy is challenging, especially in polytocous species. Based on the difference between number of ova, embryos and actually born offspring, total embryonic loss rate is estimated to be over 20% [2]. In the hare, total resorption rate calculated from the difference between the number of ovulations and viable embryos is 42% [3]. In embryo transfer studies, early embryonic loss rate in cattle amounts to over 30% [4] and is estimated to reach 14% post implantation [5]. In vivo ultrasonographic studies in the dog and horse present a post implantation resorption rate of over 10% [6] and 8% [7] respectively. These findings correspond to the resorption rate of 10% observed in our study.

In the human, the embryo becomes visible by ultrasound not before the 3 mm stage, when implantation has already taken place. Goldstein [8] monitored normal pregnancies by vaginal ultrasound starting in the 4th week of development (*p.o*.). From a total of 232 pregnancies, 27 were spontaneously lost during the embryonic period (12%). Embryonic transfer (two blastocysts) in the human results in a pregnancy rate of 30% and a “baby take home rate” of 21% [9].

Spontaneous resorption is a major problem in assisted reproduction in the human. Therefore, the resorption prone murine CBA-DBA model is widely used to investigate underlying mechanisms. Latest research focuses on the self/ non-self immune recognition as the primary resorption generating process [10–13]. While the presence of various immune cells and cytokines was described in the decidua, the definitive role of the specific immune system in the resorption process is still not clear [14].

The goal of the present study is to identify the mechanism of spontaneous resorption in normal pregnancies. For this purpose, we analyse murine resorption sites detected by in vivo high-resolution ultrasound in our previous study [15] by histology and immunohistochemistry.

## Results

### Study outline

In the current study, we describe the histology and immunohistochemistry of spontaneous resorptions, which were identified by reduced growth rate and reduced heart rate in daily scans of pregnant mice with high-resolution ultrasound in a previous study [15]. The pathological details of the implantation sites under resorption are compared with the respective structures in normal littermates. Normal implantations were detected from day 5 onwards. Embryos under resorption could not be detected by ultrasound before day 7. The signs of incipient resorption were reduced growth and decreasing heart rate. The 23 resorptions were scattered among 15 pregnancies. This corresponds to a spontaneous resorption rate of 10%, which is in the normal range reported in literature [16]. The outline of the study is illustrated in the graphical abstract.

Table 1 provides a link between the present histological description and the original ultrasound data of the same specimens. The table contains data such as litter of derivation, day of first detection by ultrasound, location in the right or left uterine horn, day of retrieval for histology, and normal littermates processed for comparison. In both studies, resorptions are designated as R1 through R23. This allows allocation of the ultrasound observations published in the first paper, to the histology of the same implantation sites described here. The histological samples cover the whole implantation sites with adjacent uterine canal. This is in contrast to most other studies, which describe either the development of the embryo or of the placenta.

**Table 1:** Ultrasonographic detection of resorptions and days of collection for histology

Mother ID	**ID of 1st resorption**	Detection (day)	Location in uterus horn	**ID of 2nd resorption**	Detection(day)	Location in uterus horn	**ID of 3rd resorption**	Detection (day)	Location in uterus horn	Collection (day)	Normal littermates collected
Early resorptions											
1743	**R1 1**^**+**^** (Composite 2)**	d7	E5 left	**R2 + (Composite 1)**	d7	E1 right				d8	E4 left, E1 left
5015	R3^+^	d7	E1 right							d8	E4 right
1049	R4^−^	d7	E3 right							d8	
2334	R5^+^	d8	E2 left		d8	E2 right				d9	
8397	R7^+^	d8	E1 right	this cell should not be merged in vertical direction (also below)						d9	
2521	R8^+^	d8	E3 left							d9	
Late resorptions											
2034	R9^−^	d7	E1 left	R10^−^	d7	E2 left	**R11**^**+**^ **(Composite 4)**	d9	E5 left	d9	
9859	R12^+^	d7	E2 right	**R13**^**+**^ **(Composite 5)**	d9	E1 right				d9	E1 left
0530	R14^+^	d8	E6 left	**R15**^**+**^ **(Composite 3)**	d9	E2 left				d10	E5 left, E4
7003	**R16**^**+**^ **(Composite 6)**	d9	E3 right	R17^+^	d9	E4 right				d11	E5 right, E2 right
5039	R18^−^	d10	E5 right							d10	E1 und E2
0512	R19^−^	d9	E2 left	R20^−^	d10	E3 left				d10	E3 right
Placentae											
6119	R21^**+**^	d12	E4 left							d12	E3 left, E4 left
8207	R22^**+**^	d12	E3 left							d12	E2 right
5828	R23^**+**^	d13	E1 right							d13	E5 right

Table 1 and nomenclature correspond to Table 1 in [15]. Because of the complexity of histology more details were included such as original mother ID, embryo number and location in the uterine horn. This allows identification and comparability of the resorptions described. The resorptions underlying the compsite figures are indicated. Histology (+) and no histology (−) is indicated by uppercase symbols

For histological description, we grouped the specimens into early and late resorptions. The early resorptions were retrieved for histology at day 8 (R1 - R4, Table 1) and the late resorptions between days 9 and 11 (R5 – R20, Table 1). In the resorptions collected at days 12 and 13 (R21 – R23, Table 1), only the placentae were preserved.

**Fig. 1:**
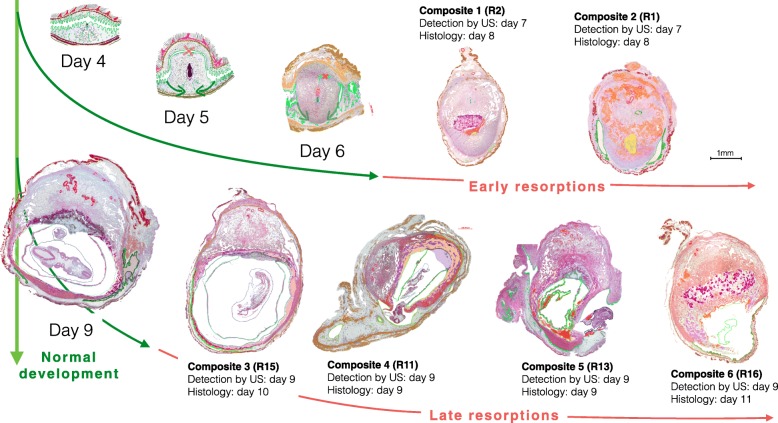
Synopsis of early and late resorptions in relation to normal development, Upper panel: Normal antimesometrial implantation (schematic after Rugh) and two early resorptions first detected by ultrasound (US) at day 7. Composite 1: Neutrophilic liquefaction of embryo proper (R2, Table 1). Composite 2: Additional resorption and liquefaction of maternal tissues via foam cells (resorption R1, Table 1). Composites 3–6: Stages of abortion of apoptotic embryos into the uterine lumen

**Fig. 2:**
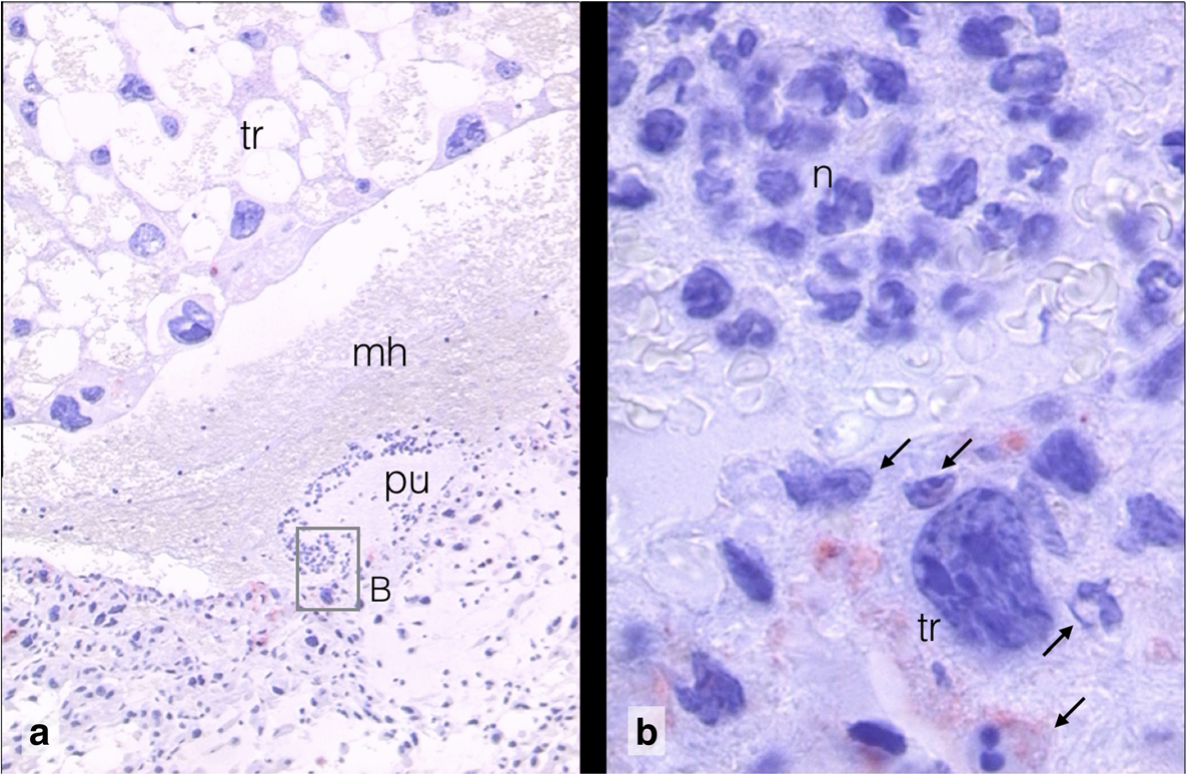
Trophoblast array, haemorrhage, and suppurated focus of Composite 1, Higher magnification of underlying section of composite 1 (Additional file 1, Layer Histology). **a** self-organizing trophoblast array (tr), maternal haemorrhage (mh), and purulent focus (pu), 10x. **b** Inset: purulent focus with neutrophil granulocytes (n) and maternal erythrocytes. Trophoblast cell (tr) with faint caspase 3 staining in the cytoplasm, invaded by neutrophils and lymphocytes (arrows). 100x. Caspase 3. For localization see Additional file 1: Detailed Observations, Slide 2

**Fig. 3:**
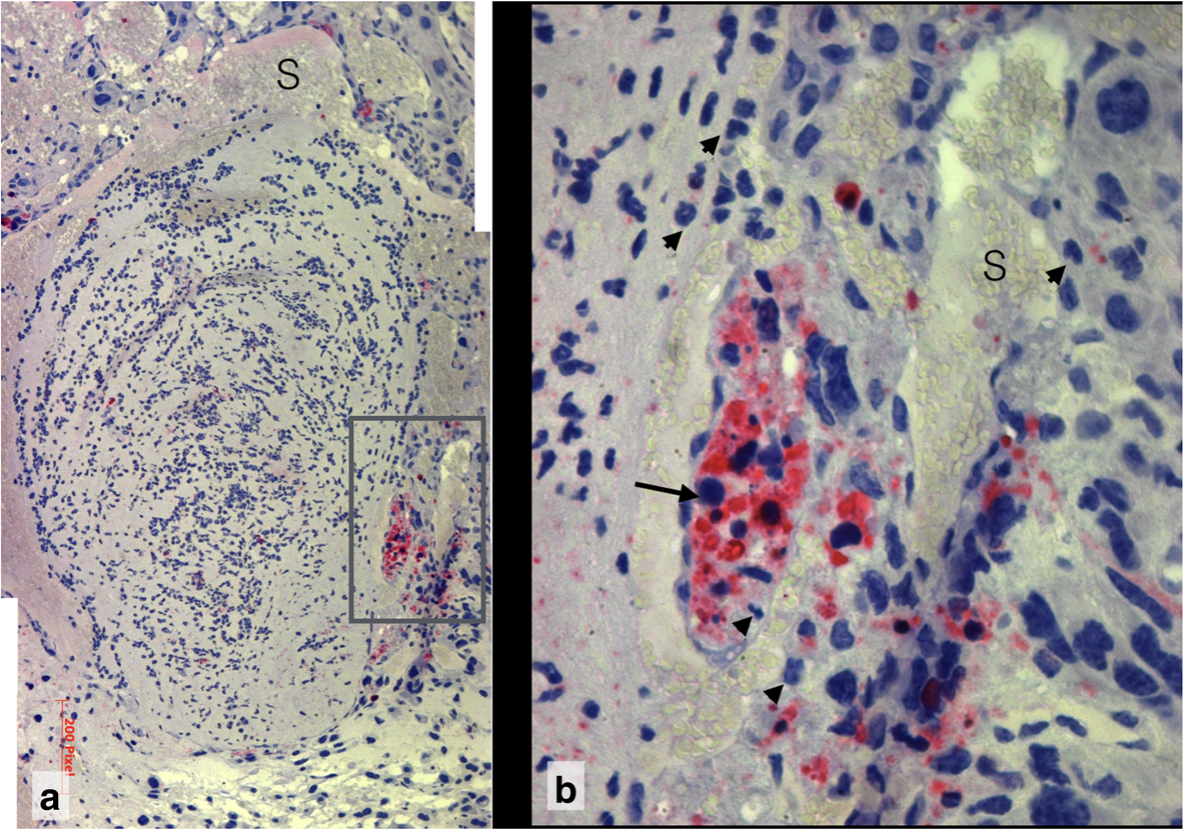
Purulent focus of composite 2, **a** Massive accumulation of maternal neutrophil granulocytes in a gel-like amorphous matrix surrounded by congested maternal sinusoids (si). Caspase 3 immunoreactivity. 20x. **b** Inset: delimited spot of terminal apoptosis in the sinusoidal decidua with caspase 3 positive foam cells (black arrow), cell detritus and neutrophil granulocytes (white arrow heads). 40x. For localization see Additional file 1: Detailed Observations, Slide 4

**Fig. 4:**
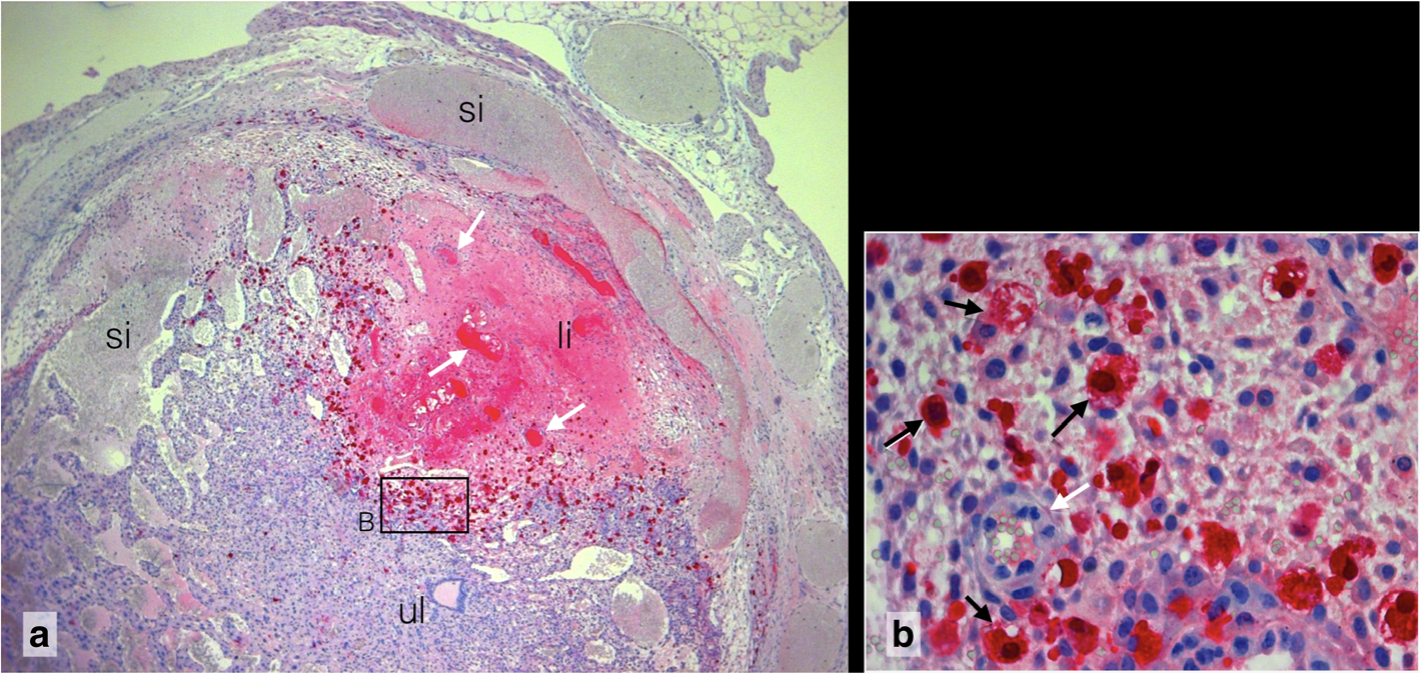
Liquefaction of decidua basalis via foam cells of Composite 2, **a** Centre of liquefaction (li) in the sub-mesometrial decidua surrounding the arteries (white arrows). si congested sinusoids, ul primary uterine lumen. Caspase 3 5x. **b** Inset: Caspase 3 positive foam cells (black arrows), arteries (white arrows).63x. For localization see Additional file 1: Detailed Observations, Slide 4

**Fig. 5:**
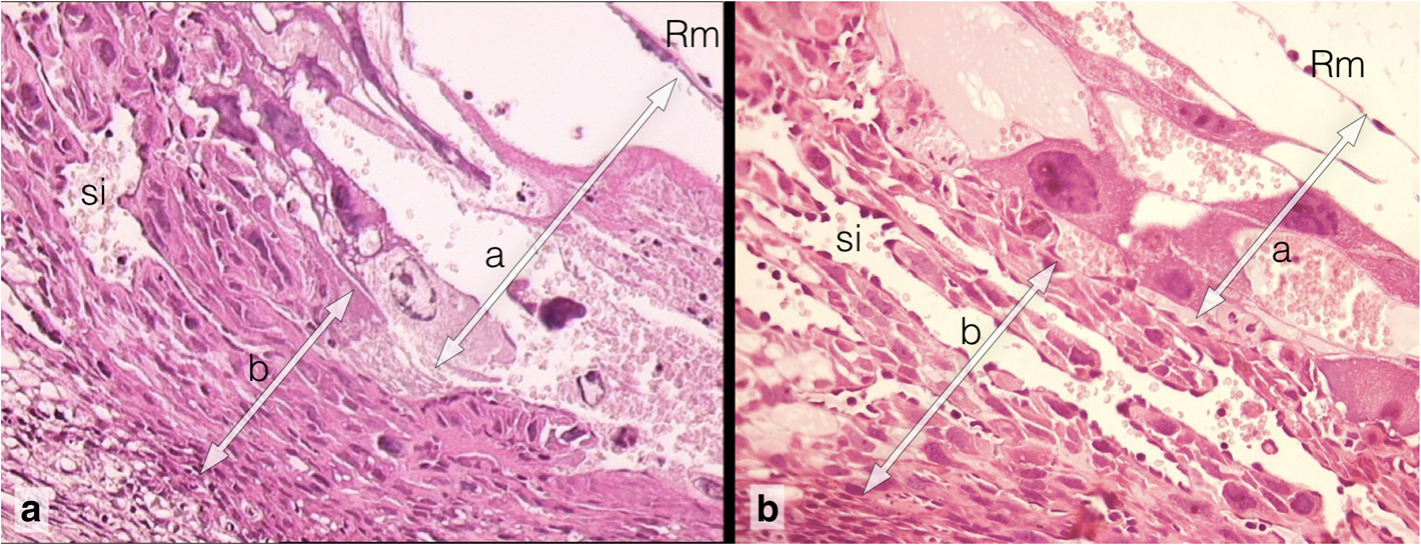
Degenerated lacunar trophoblast and intact decidua capsularis of Composite 3, **a** Resorption site with intact decidua capsularis and autolytic lacunar trophoblast. HE. 63x **b** Day 9 normal development. HE 63x. Frame **a** and **b** (indicated in Slide 7) are located in the yolk sac angle and display the same overall structure. Rm Reichert membrane, si maternal sinusoid, a lacunar trophoblast; b decidua capsularis. For localization see Additional file 1: Detailed Observations, Slide 7

**Fig. 6:**
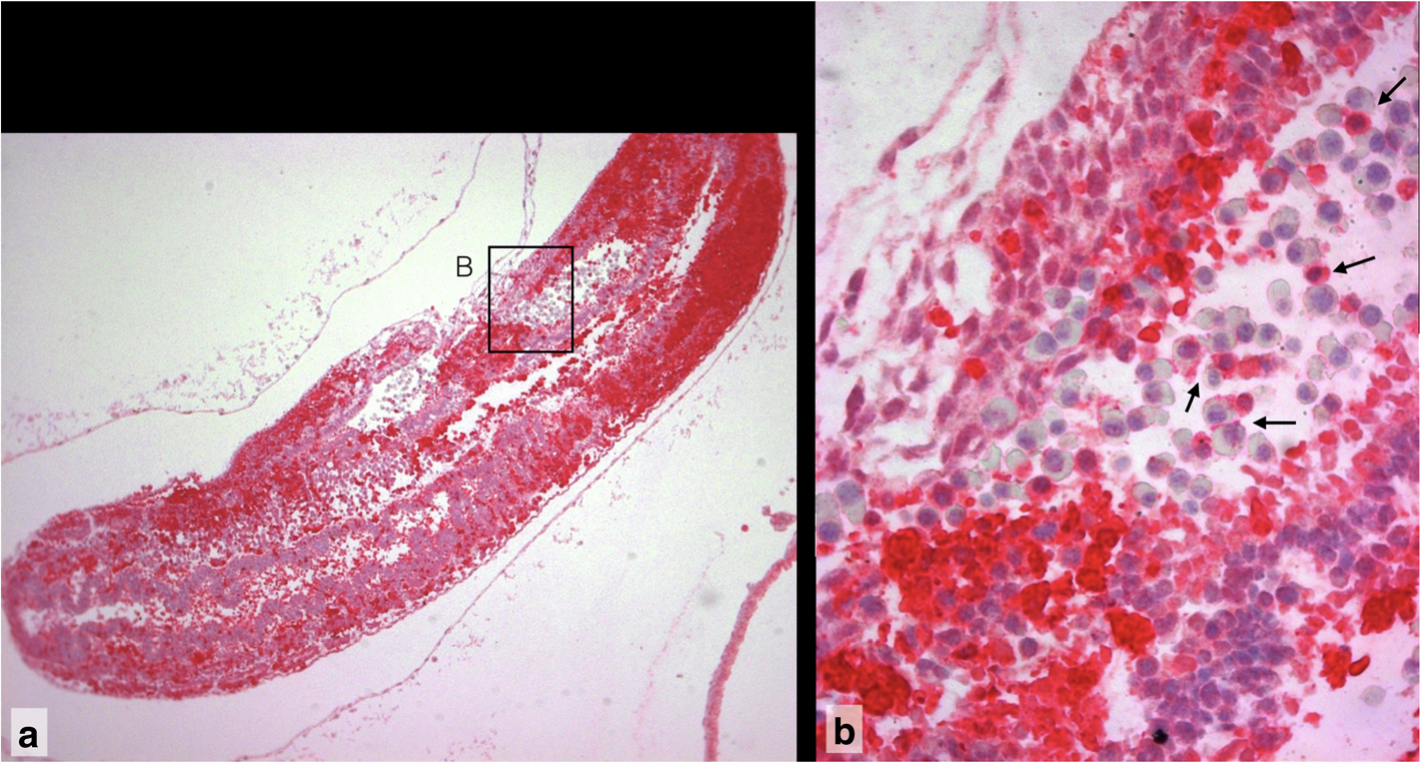
Caspase 3 immunoreactivity of Composite 3, **a** In the apoptotic embryo, almost all cells show caspase 3 immunoreactivity. **b** Transformed embryonic haematoblasts are caspase 3 negative and aggregate with caspase 3 positive embryonic cells (black arrows) 100x

**Fig. 7:**
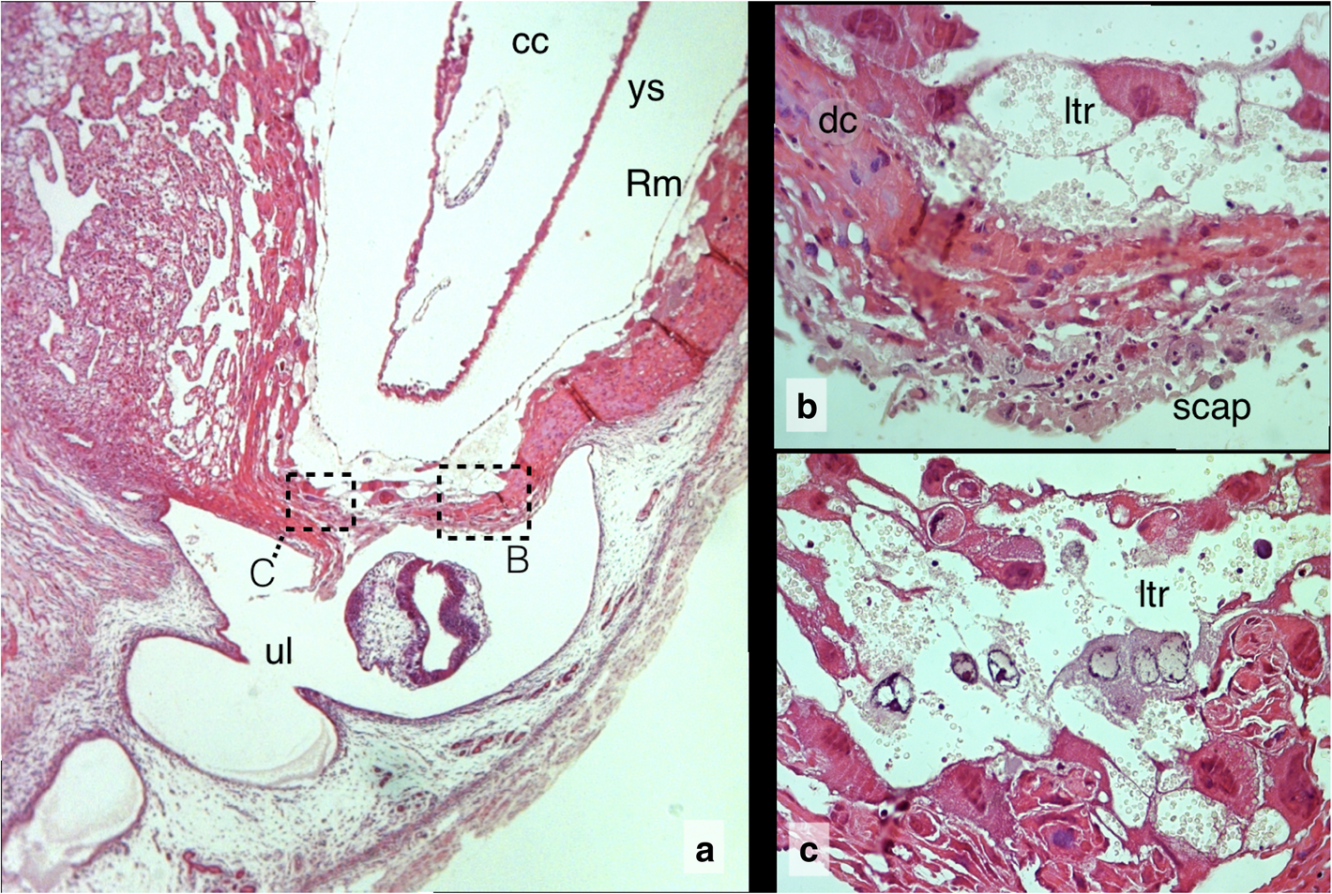
The rupture site of Composite 4, **a** Section above rupture site. cc chorionic cavity, ys yolk sac, Rm Reichert membrane, ul uterine lumen. HE 5x. **b** Inset: Fibrinoid scap with neutrophils on decidua capsularis (dc) 40x HE. **c** Inset: Nuclear swelling in lacunar trophoblast cells (ltr). 40x HE

**Fig. 8:**
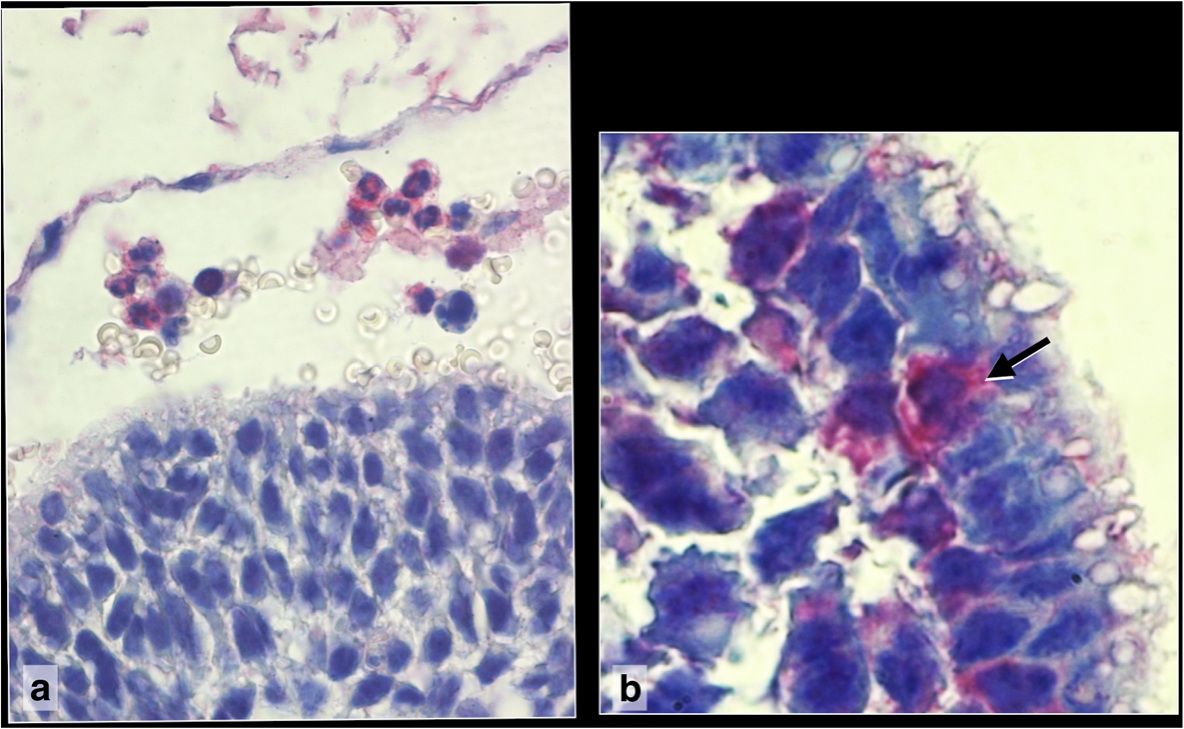
MPO7 positive maternal neutrophils in the apoptotic embryo of Composite 4, **a** MPO7 positive neutrophils and maternal eythrocytes in the amniotic cavity. 100x. **b** Maternal MPO7 positive neutrophil granulocyte (arrow) in ectoderm of head anlage (for localisation compare Slide 21A). 100x with empty magnification. For localization see Additional file 1: Detailed Observations, Slide 15)

**Fig. 9:**
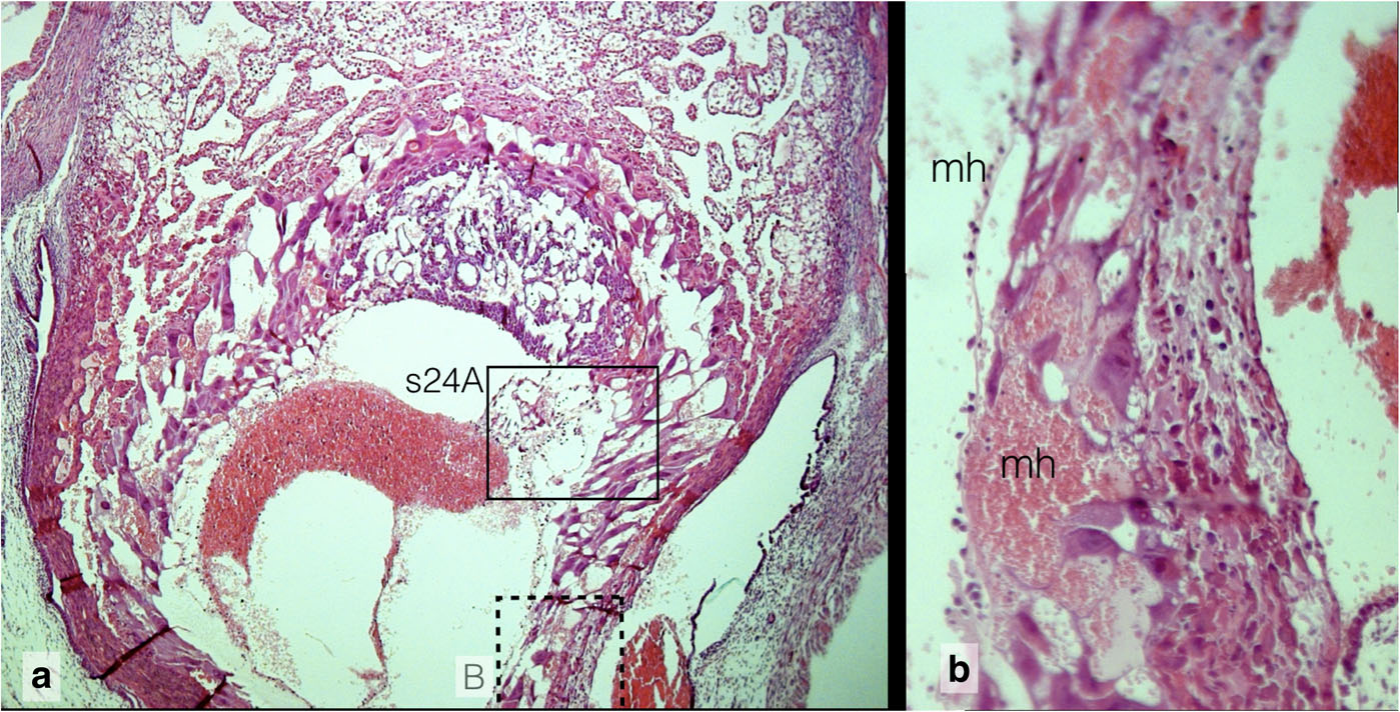
Maternal haemorrhage in Composite 5, **a** Extended and distorted trophoblast lacunae in the rupture zone of the placental anlage. (s24A see Additional file 1) 5x HE **b** Inset (consecutive section of A): Decomposed decidua capsularis with haemorrhagic infiltration. Rm Reichert membrane, mh maternal haemorrhage 40x. For localization see Additional file 1: Detailed Observations, Slide 22

**Fig. 10:**
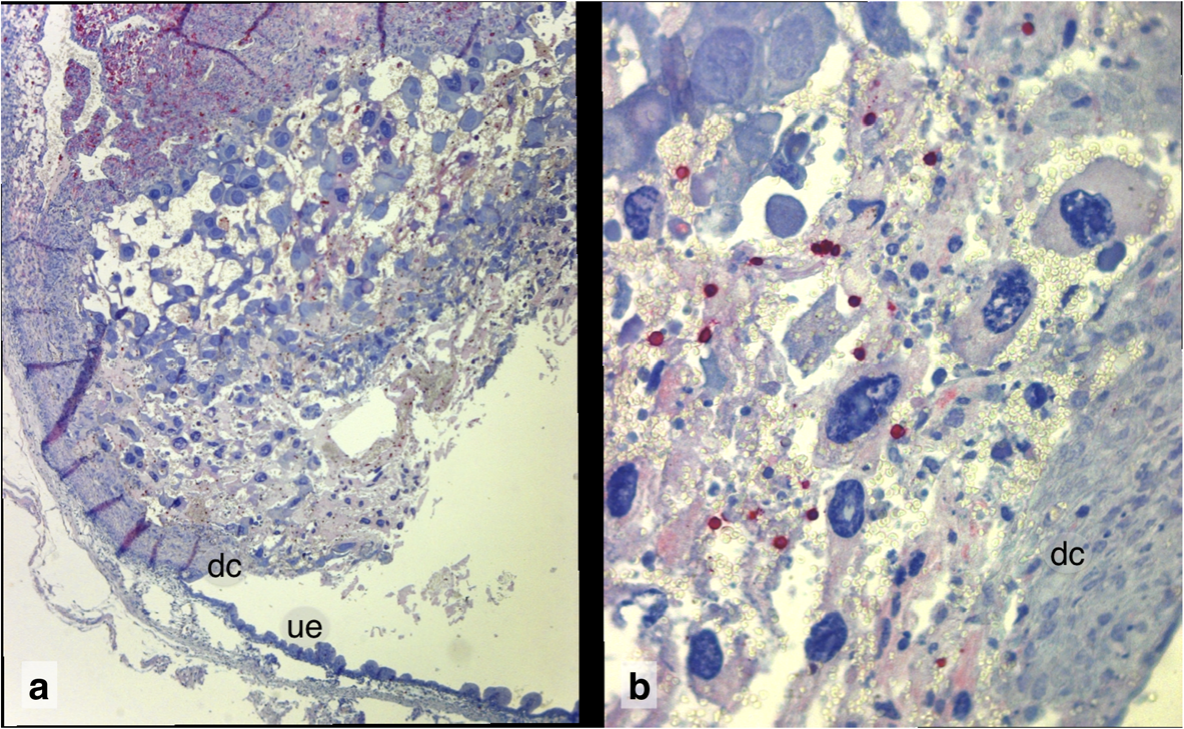
B220 Immunoreactivity in the “final cup” stage of Composite 6, **a** denuded open wound area between disintegrating trophoblast area and uterine lumen. Villus-like extrusions in the high columnar uterine epithelium. (For B220 immunoreactivity of foam cells in the compact zone of the decidua basalis compare Slide 15 and 17A, Additional file 1). dc decidua capsularis, ue uterine epithelium. 5x **b** Sterile neutrophilic inflammation between degenerating trophblast and decidua capsularis. B220 negative neutrophils and B220 positive small lymphocytes. 40x

From the total of the 23 resorptions, we selected six typical specimens, two out of the group of early and four out of the group of late resorptions. Within the groups of early and late resorptions, the description follows the ordering according to the stage of resorption and not according to age. In Fig. 1, the six resorptions are depicted as composites (Composite 1–6) based on central sections of the whole implantation sites with the mesometrial root of the uterus oriented upwards. Histological structures are indicated with different colours and assigned to specific layers. Figures 2, 3, 4, 5, 6, 7, 8, 9 and 10 show the main observations described in Overview of results. Additional file 1 contains the enlarged composites, which serve as guideline for the documentation of the detailed results as slides. The respective slide numbers are given in the list of slides. The figures can be localized in the enlarged composites of Additional file 1 via inset marks.

In Additional file 2, an interactive pdf version of the resorption composites and the composites of a normal day 6 and 9 is provided. Details e.g. in the underlying histology can be viewed after switching off the labelling layers in the side bar of acrobat reader.

### Overview of results

The upper panel of the synopsis of Fig. 1 depicts two early resorptions (Composite 1 and 2). Both were detected by ultrasound at day 7 as implantation sites with reduced size when compared with their normal littermates. They belonged to the same pregnancy and were retrieved for histology at day 8. For comparison normal antimesometrial implantation and a normal day 6 embryo are shown. The overall structure of the early resorptions still corresponds to normal development but the embryo proper has already disappeared. The retained features are: persistence of the primary uterine lumen in the mesometrial decidua and incipient formation of a secondary lumen at the antimesometrial side. Compression of the antimesometrial decidua to a decidua capsularis has not yet occurred. Therefore, the embryo has probably already regressed at day 6. Nevertheless, the extraembryonic and maternal parts of the failing implantation sites have developed further and nearly reach the size of a normal day 7 embryo. Resorption occurred directly without abortion into the uterine lumen, which in these early stages is transiently occluded by the antimesometrial implantation.

Composite 1 is characterized by maternal haemorrhage at the site of the former embryo and development of a suppurated focus with accumulation of neutrophil granulocytes (Fig. 2). An abnormal array of self-organizing lacunar trophoblast has formed at the mesometrial side. Composite 2 is further developed than Composite 1. The secondary lumen of the uterine canal already appears on both sides of the implantation cone. The central suppurated focus with accumulation of neutrophil granulocytes has enlarged (Fig. 3). In the basal decidua surrounding the central artery, a second centre of tissue liquefaction appears. It is characterized by accumulation of foam cells (Fig. 4).

The lower panel of the synopsis (Fig. 1) depicts four late stages of abortion of apoptotic embryos into the secondary uterine lumen first detected at day 9 and retrieved for histology at days 10, 9, and 11 (Composites 3-6). They were considerably smaller than the normal day 9 embryo depicted on the left side of the synopsis.

Resorption R15 of Composite 3 was collected at day 10. The heart was still beating. The embryo is in a final state of apoptosis whereas the maternal moiety of the implantation site is intact (Fig. 5). The final state of apoptosis is indicated by caspase 3-expression (Fig. 6). Decomposition is accompanied by caspase 3-negative transformed embryonic blood cells probably engaged in removal of cellular debris. They show cellular polymorphism with eccentric nuclei, cytoplasmic vacuoles, and blebbing at the cell surface (Additional file 1, Slide 9 and 11). The function as embryonic innate immune cells is indicated by expression of the myeloperoxidase MPO 7 (Additional file 1, Slide 12).

Composite 4 depicts a resorption at day 9 caught in the process of abortion (Fig. 1). The embryonic vesicle is ruptured and the embryo halfway extruded into the uterine lumen. The embryonic part of the chorioallantois placenta has lost its contact to the decidua basalis and is dislocated. In both zones in which the implantation bulge faces the open uterine lumen, the zone of rupture and the opposite zone of imminent rupture, single trophoblast cells exhibit nuclear swelling and disintegrate. The trophoblast lacunae are discontinuous and extended. The rupture site of the embryonic vesicle is characterized by a typical sterile inflammation of the decidua capsularis with insudation of the tissue and invasion of neutrophils (Fig. 7). After break down of the foetal-maternal border maternal neutrophils invade the embryonic tissue (Fig. 8).

Composite 5 illustrates the role of maternal haemorrhage in the abortion process (Fig. 1). The sources of bleeding are the marginal zone of the chorioallantois placenta where maternal and embryonic vessels interdigitate, leading to the formation of mixed blood, and rupture of extended trophoblast lacunae (Fig. 9). Lakes of blood with aggregated erythrocytes are found in the chorionic and the yolk sac cavity. The blood in the lacunae is not coagulated. The embryo with its amnion is completely expelled into the uterine lumen where it is still present.

Composite 6 represents the “final cup”- stage of resorption found in most of the resorptions studied. The embryo has disappeared. The implantation site consists of mesometrial maternal decidua and remnants of single cell or abnormally reorganized trophoblast. An area of denudation covered by fibroid and neutrophils faces the uterine lumen like an open wound (Fig. 10). Invasion of neutrophils and a small proportion of small B220 positive B-lymphocytes characterize the wound area.

### Detailed observations

#### Early resorptions

In the two early resorptions depicted in the upper panel of the synopsis (Fig. 1), the antimesometrial decidua has fused with the mesometrial decidua, where finally the chorioallantois placenta develops. The antimesometrial implantation bulge is not yet separated from the uterine wall. R1 (E5 left, Composite 2) is more advanced in development than R2 (E1 right, Composite 1). The two resorptions are littermates located in different uterine horns in different positions.

### Composite 1 (R2): Haemorrhage, purulent focus, and self-organizing trophoblast

In Composite 1 (Additional file 1, Slide 2 and Additional file 2) the dense antimesometrial decidua has increased in thickness as compared to normal day 6 embryo (Additional file 2). Compression to a decidua capsularis has not yet occurred. Remnants of uterine epithelium indicate the location of the former primary uterine lumen in the compact zone of the mesometrial decidua. Instead of the ectoplacental cone and embryonic trophoblast, a large array of lacunar trophoblast has developed. The lacunae are filled with non-coagulated maternal blood. The trophoblast cells appear normal without signs of degeneration. The outer trophoblast cells establish with their cell extensions an epithelial-like border towards the sinusoidal layer of the decidua.

The inset in Composite 1 (Additional file 1, Slide 3) covers the lower border of the pathological trophoblast, maternal haemorrhage and purulent focus. In the haemorrhage maternal erythrocytes are aggregated in contrast to the erythrocytes in the lacunae in the trophoblast. The purulent focus contains decomposed tissue and maternal neutrophils as demonstrated by higher magnification. Maternal neutrophils and lymphocytes invade the cytoplasm of single loose trophoblast cells.

### Composite 2 (R1): Resorption with additional liquefaction of decidua

The Composite 2 (Additional file 1, Slide 4 and Additional file 2) the sinusoids are dilated and congested with maternal blood. The degenerating primary lumen is visible. Two extensions of the uterine lumen are under way to separate the antimesometrial decidua from the uterine wall by formation of the secondary lumen. The suppurated focus is large and compact and is located in the antimesometrial decidua at the former site of the embryo (Additional file 1, Slide 5).

In the decidua basalis around the central spiral artery an array of liquefaction evolves (Additional file 1, Slide 6). The rim of this area is formed by caspase 3 positive foam cells, which disintegrate towards the centre of liquefaction. In the remaining decidua basalis caspase 3 positive foam cells are engaged in apoptosis of single decidua cells. The majority of foam cells are caspase 3 negative. The pattern of small apoptotic spots is also encountered during remodelling and removal of cell debris by macrophages in the normal littermate.

### R3 (not documented)

R3 (Table 1) is implanted antimesometrially and shows massive haemorrhage.

#### Late resorptions

Late resorptions were collected at days 9, 10, and 11. Some were already detected by ultrasound at day 7 or 8 (Table 1). They are characterized by abortion of the dead embryo into the uterine lumen, where the decomposed embryonic tissues are resorbed. The Composites 3–6 prepared from the specimens R15, R11, R13 and R16, respectively, demonstrate consecutive stages of abortion (Synopsis in Fig. 1).

### Composite 3 (R15): the apoptotic embryo in situ

The resorption site was first detected at day 9 by reduced size of the embryonic vesicle as compared to its littermates and by reduced heart rate. Heartbeat was still detectable at the day of collection (day 10). The specimen (Additional file 1, Slide 7 and Additional file 2) is extraordinary due to the fact that the dead embryo is not yet aborted into the uterine lumen. Therefore, it is particularly informative with respect to cellular details of the final stage of embryonic apoptosis.

The general appearance of the extraembryonic membranes corresponds to the development of the normal day 9 embryo (Additional file 2). In the contact zone with the uterine epithelium, a fibroid layer replaces the decidua capsularis. This corresponds to physiological involution of the decidua basalis in the normal day 10 and day 11 littermates. In both the resorption and the normal littermate, the outer layer of the decidua capsularis consists of densely packed spindle shaped cells. In the sinusoidal layer, maternal sinusoids are lined by intact endothelium and communicate with the lacunar spaces of the trophoblast (Additional file 1, Slide 8). As constitutive part of the yolk sac placenta the normal lacunar trophoblast consists of a network of mural giant cells with intercellular lacunae perfused by maternal blood. In the resorption the network is disrupted. The giant cells are pycnotic or show nuclear swelling.

Morphological signs of apoptosis of the embryo proper are the disintegration of embryonic tissues with large intercellular spaces and fragmentation of cell nuclei indicated by nuclear fragments of different sizes (Additional file 1, Slide 9A). Within the apoptotic tissue, transformed embryonic blood cells are interspersed. They exhibit an eccentric nucleus and large cytoplasmic vacuoles (Additional file 1, Slide 9B). The apoptotic embryonic cells exhibit strong caspase 3 immunoreactivity as sign of final apoptosis (Additional file 1, Slide 10 A). The transformed embryonic blood cells are caspase 3 negative (Additional file 1, Slide 10B).

Transformed embryonic blood cells are not only encountered in the apoptotic embryo but throughout the embryonic circulation including the allantois mesoderm (Additional file 1, Slide 11). Cellular polymorphism of the embryonic blood cells includes eccentric nuclei, cytoplasmic vacuoles and formation of blebs at the cell surface. These morphological characteristics of the blood cells were a constant feature in all embryos under resorption in which yolk sac blood islets had already developed. By contrast, in the normal littermates the blood cells were homogeneous and characterized by artificial osmotic shrinkage not present in the resorptions (Additional file 1, Slide 11B). Within the apoptotic embryonic tissue, a large proportion of the transformed blood cells show a positive immunoreactivity for MPO7 (Additional file 1, Slide 12).

### Littermate resorption R14

Resorption R14 (Table 1) from the same mother was first detected by US one day earlier at day 8 and thus represents a later stage of resorption with an earlier starting point. The embryo proper and the yolk sac have totally disappeared. The empty embryonic cavity is lined by lacunar trophoblast, which is composed of 4–5 cell layers thick at the basal, and of 1–2 cell layers at the capsular side of the decidua. Fading and fragmented trophoblast cell nuclei indicate advanced apoptosis.

### Composite 4: Resorption with halfway aborted embryo

By US, the hypoechogenic fluid of the embryonic cavity consisted only of a thin, oval shaped layer that did not completely surround the embryo proper, as it was the case in its normal littermates [1]. Retrospectively, this observation represents the opening of the implantation site towards the uterine lumen as seen in the histologic sections. In the histologic section the embryo proper is halfway expelled from the embryonic into the uterine cavity with its outstretched body still inside and its head already outside (Additional file 1, Slide 13 and Additional file 2). The embryo is retarded by about one day of development corresponding to a day 8 embryo. The slit in the wall of the embryonic vesicle connects chorionic cavity with uterine lumen. The slit is located at the base of the implantation bulge and runs through the lacunar trophoblast, decidua capsularis, and the covering uterine epithelium. The amnion is broken with non-continuous chunks covering the inside and the outside moieties of the embryo. Yolk sac and Reichert membrane form a cup like structure through the opening of which the embryo is released. The whole embryo and its membranes including the allantois mesoderm of the developing chorioallantois placenta are rotated clockwise within the embryonic vesicle by about 90°. The lacunae of the trophoblast are extremely extended. The zone of displacement cuts horizontally through the extended and broken lacunae of the trophoblast. The implantation site was preserved for histology as segment of the otherwise intact uterine horn and conventionally fixed with formalin. A contribution of the preparation procedure to expulsion of the embryo cannot totally be excluded. Therefore, as in forensic medicine, the description focuses on pre-mortal tissue reactions such as developmental retardation, degenerative processes and infiltration with immune cells indicating later stages of inflammation.

### Normal involution of lacunar trophoblast and decidua capsularis in littermates

In normal development the wall of the embryonic vesicle is formed by embryonic lacunar trophoblast and maternal decidua capsularis. Lacunar trophoblast, Reichert membrane, and the internal leaflet of the yolk sac membrane form the embryonic layers of the yolk sac placenta. Due to the internal pressure in the embryonic cavities, the embryonic vesicle expands rapidly within the uterine canal. The trophoblast cells become spindle shaped and the lacunae compressed, so that the lacunar character disappears. The decidua capsularis, lacunar trophoblast and the covering uterine epithelium normally regress at days 10 and 11. Thereafter, up to birth the internal leaflet of the inverted yolk sac forms the outer layer of the extraembryonic membranes. During normal regression of the decidua capsularis we observed in the decidua basalis as well as in the decidua capsularis caspase 3 positive small spots of apoptosis, accompanied by a moderate infiltration with maternal neutrophils. In the resorption scenario this small inflammatory contribution to normal regression of the decidua capsularis becomes a full-blown sterile unspecific inflammation.

### Premature regression and rupture of the decidua capsularis during resorption

In Composite 4 (Additional file 1, Slide 13) the wall of the embryonic vesicle has ruptured. The free ends of the decidua capsularis show swollen and disintegrating cell nuclei and are infiltrated with neutrophil leucocytes. In the rupture zone the uterine epithelium covering the decidua capsularis, has faded away. The section directly above the rupture site (Additional file 1, Slide 14) demonstrates the underlying sterile inflammatory process in more detail. A fibroid matrix covers the decidual tissue with cellular debris infiltrated by maternal neutrophils. The decidua capsularis denuded from uterine epithelium, opens like a wound with a scap layer into the uterine lumen (Additional file 1, Slide 14B). The underlying trophoblast lacunae are disrupted and the trophoblast cells exhibit different forms of cell death such as nuclear swelling, pycnosis, fading, cellular fragmentation, rounding up, and shrinkage. The oblique section (Additional file 1, Slide 14A) also covers the adjoining uterine canal with contraction rings of the uterine muscle layers.

The whole specimen is cut in an oblique longitudinal plane so that opposite to the rupture site the uterine lumen is also in view (Additional file 1, Slide 15). As in the rupture zone, the uterine epithelium covering the decidua capsularis has faded away. The reflecting folds of the uterine lumen are filled with coagulated maternal blood, which merges into the fibroid matrix derived from the degenerating decidual layer. The decidua capsularis, now in direct contact with the outer epithelium of the uterine lumen, disintegrates and is infiltrated by leucocytes (Additional file 1, Slide 16B). Formation of the secondary lumen is not yet completed. The central area of the antimesometrial endometrium, from which the decidua capsularis arises, is still connected with the uterine wall. Here, a layer of true epithelioid decidual cells is retained, which is also infiltrated by single maternal leucocytes (Additional file 1, Slide 16A).

The section (Additional file 1, Slide 15) is stained for B220 immunoreactivity. The antibody B220 is used to identify B-lymphocytes. In addition to a population of small maternal lymphocytes in the maternal blood of the trophoblast lacunae (Additional file 1, Slide 17B and 18), in our hands, B220 stains the peripheral cytoplasm of foam cells in the basal decidua mainly located in compact zone. (Additional file 1, Slide 17A). In the B220 patterns no difference exists between normal littermates and resorptions.

### Early stage of apoptosis in the embryo

The halfway-extruded embryo of Composite 4 is in an early stage of apoptosis (Additional file 1, Slides 18–21). This is in contrast to the resorption of Composite 3, which has reached the final stage of apoptosis with completely decomposed embryonic tissues. Caspase 3 stains circumscribed arrays of cellular breakdown not only in the embryonic head but also in the embryonic body, where in the normal embryo physiological apoptosis does not occur (Additional file 1, Slide 18 and 19). With high magnification, single caspase 3 negative and caspase 3 positive embryonic blood cells are discernible (Additional file 1, Slide 19A). As histological signs of early apoptosis, the intercellular spaces in the neural epithelium are extended and the epithelial cells form cytoplasmic blebs at the apical poles (Additional file 1, Slide 18B and 20A). A few retained mitotic figures display blurred chromosomes indicating apoptosis (Additional file 1, Slide 18B).

### Invasion of the apoptotic embryo by maternal neutrophils

Due to the rupture of the embryonic vesicle the embryo is aborted into the uterine lumen. The apoptotic embryo comes into direct contact with maternal blood. In this setting, maternal neutrophils invade the embryonic apoptotic tissues. The caspase 3 immunoreaction stains single maternal neutrophils invading the neuroepithelium (Additional file 1, Slide 18B). Similarly, single maternal neutrophils with segmented nuclei invading the embryonic tissue can be identified by MPO7 immunoreactivity (Additional file 1, Slide 20). Caspase 3 is a marker of the final stage of apoptosis. MPO7 is expressed in activated monocytes and neutrophils and is indicative for cell destabilization. Both markers are expressed only in a small proportion of the neutrophils in the inflammatory infiltrates of the resorptions.

In Composite 4 maternal blood with MPO7 positive leucocytes and embryonic blood cells (extravasated mixed blood, see below) has entered the amniotic cavity and is in direct contact with the embryo proper (Additional file 1, Slide 15 and 20A). MPO7 positive maternal neutrophils adhere to embryonic blood cells, thus insinuating a functional relationship.

### F80/4 positive macrophages in the contact zone of uterine epithelium and embryonic tissue

The macrophage-specific antibody F4/80 stains fibrocyte-like macrophages with a maximum in the mesometrial muscle layers and a declining frequency in the endometrium and towards the antimesometrial pole. There is no difference between F4/80 immunoreactivity between normal implantations and implantation sites under resorption. However, where the extruded embryo is in direct contact with the uterine epithelium, F4/80 positive macrophages accumulate underneath the epithelium (Additional file 1, Slide 21). In the contact zone, the embryonic epithelium flattens and the uterine epithelium increases in height. A similar sub-epithelial accumulation of macrophages is also present in the normal embryo in the contact zone of the uterine epithelium with the physiologically regressing decidua capsularis. Together, the histological and immunohistochemical findings depict a sterile inflammation in the resorption process and strongly imply that the expulsion of the embryo is not an artefact.

### Littermate resorption R10

Littermate R10 (Table 1) was in the empty cup stage as described below in Composite 6.

### Composite 5 (R13): Resorption with aborted embryo and maternal haemorrhage

In the ultrasound scan, the embryo of resorption R13 was clearly visible in the uterine canal outside and between the implantation bulges of R12 and R13.

In the histological sections, the embryo is completely located in the uterine lumen next to the broken and collapsed wall of the embryonic vesicle (Additional file 1, Slide 22 and Additional file 2). The yolk sac is still inside the embryonic vesicle formed by the embryonic lacunar trophoblast and maternal decidua capsularis. The shattered amnion is also expelled and partially covers the embryo in the uterine lumen. In the rupture zone, the decidua capsularis degenerates and the lacunar trophoblast is discontinuous. Vis-á-vis to the rupture site in the contact zones with the maternal uterine epithelium the decidua capsularis has also faded away and a fibroid layer has formed.

### Maternal haemorrhage

The resorption of Composite 5 is characterized by massive evasion and clotting of maternal blood (Additional file 1, Slide 22). Lakes of maternal blood with aggregated erythrocytes have entered the yolk sac cavity and displace the definitive yolk sac with mesoderm and blood islets into the imploded chorionic cavity. As in other resorptions the maternal blood does not clot where the lacunar structure of the trophoblast is still intact. Clotting occurs in areas with degenerating lacunar and ruptured trophoblast lacunae at the Reichert membrane-trophoblast interface where the maternal blood spills over into the spaces between both structures.

One possible source of bleeding into the embryonic vesicle is the marginal zone of the early chorioallantois placenta (Additional file 1, Slide 23). The placental sinusoids are extended and ruptured so that maternal and embryonic erythrocytes spill out into an area of scattered and folded remnants of Reichert membrane (Additional file 1, Slide 24A). The cytoplasm of fading trophoblast cells is stuffed with intracytoplasmatic vesicles reminiscent of maternal erythrocytes, although of different sizes (Additional file 1, Slide 24B). This phenomenon is also visible at day 8 of normal development. At the rupture site of the embryonic vesicle maternal inflammatory cells infiltrate the decidua capsularis accompanied by haemorrhage into the intercellular spaces (Additional file 1, Slide 23B).

In a parasagittal section the primary uterine lumen in the decidua basalis is extended and filled with clotted maternal blood (Additional file 1, Slide 25A). The maternal blood in the primary lumen completes the ring of extravasated blood observed in Composite 5 around the embryo and its membranes. The ring extends between Reichert membrane and yolk sac (Additional file 1, Slide 22) and between decidua basalis and placenta, so that the placental anlage is included into the abort. Thus, at this stage of resorption abortion of the embryo is powered by maternal haemorrhage.

### Mixed embryonic and maternal blood

Mixed blood is observed in the final stages of abortion. The maternal blood in the chorionic cavity contains many embryonic erythrocytes (Slide 25A). One possible source is the rupture of interdigitating maternal and embryonic vessels in the placental anlage (Slides 23 and 24). In the expelled embryo proper, maternal erythrocytes are present within the apoptotic embryo, e.g. between neural tube and somites (Slide 25B). They must have been transported to these locations by a functioning embryonic circulation. Likewise, maternal erythrocytes intermingle with embryonic blood cells in the large embryonic vessels and in the embryonic vessels of the chorioallantois placenta. No embryonic blood cells are found in the maternal blood flowing through the lacunae of the trophoblast and through the sinusoids of the decidua.

### Littermate R12: early resorption, lately collected

Resorption R12 (Table 1) was detected at day 7 and collected at day 9 together with its littermate R13 detected and collected on day 9. R12 thus represents a late stage of an early resorption with the embryo proper totally aborted (“empty cup”-stage, see below). The decidua basalis opens with an open wound into the uterine lumen. The wound is covered by a maternal blood clot, which also contains many embryonic blood cells. The centre of liquefaction is composed of decidua cells, maternal erythrocytes, macrophages and foam cells.

### Composite 6 (R16): the final “empty cup” stage

The resorption of Composite 6 (R16) was retrieved for histology at day 11, two days after first detection (Additional file 1, Slide 26). It represents the final stage of the abortion process. The embryo proper has disappeared and the remaining implantation site forms an empty cup-like structure, which opens into the uterine lumen. The surface is denuded of uterine epithelium and similar to an open wound with a haemorrhagic inflammation covered by fibroid. Decaying lacunar trophoblast is infiltrated by maternal lymphocytes and neutrophils, some of which also invade the cytoplasm of degenerating trophoblast giant cells (Additional file 1, Slide 27). In the compact zone of the decidua basalis foam cells prevail (Slide 28A). The adjacent trophoblast cells of the self-organizing array degenerate (Additional file 1, Slide 28B). In the place of the placenta, an array of self-organizing lacunar trophoblast has formed. Intercellular spaces and lacunae are filled with non-clotted maternal blood. Whereas in the early resorption of Composite 1 the trophoblast cells establish with their cell extensions an epithelial border, in Composite 6 the outer zone of the trophoblast array has a loose character and is infiltrated by maternal neutrophils.

The wall of the embryonic vesicle has totally disappeared (Additional file 1, Slide 26 and Slide 29), the rupture of which was described in Composite 4 and Composite 5 (compare Additional file 1, Slides 13 and 22). In place of the embryo only some remnants of the Reichert membrane remain. Thus, a sneaking transition between abortion and resorption in place is indicated. As sign of a resorptive function the uterine epithelium is high columnar and forms small folds (compare Slide 26). At the rim of the open cup among maternal neutrophils some small lymphocytes and lymphocyte aggregates exhibit B220 immunoreactivity (Additional file 1, Slide.

Littermate resorption R17 (Table 1).

Only placental parts have been preserved for histology.

R5, R7 and R8 (Table 1).

These resorptions were characterized by massive maternal haemorrhage.

R12 (Table 1).

The remnants of the implantation site were sequestered and shed into the uterine lumen by contraction of the uterine muscle layer.

#### Resorption in the day 12-placenta

At day 12, a functional labyrinth chorioallantois placenta has developed with a counter current between maternal sinusoids and embryonic vessels. The yolk sac placenta has disappeared in the periphery, but is still fully functional in the marginal zone of the placental disc. In the histological specimens of the resorptions detected at day 12 (R21 and 22, Table 1), the embryo was not included. Destruction of the placenta R21 is more advanced than in R22.

### Placenta with advanced destruction (R21)

The decidua basalis of the placenta R21 is in the state of dissolution (Additional file 1, Slide 30). Large areas of tissue destruction range from arrays with caspase 3 activities, zones of decaying tissue, demarcated purulent foci, and drained empty spaces. At the border of decidua basalis and placenta some disorganized giant cells are left over. In the centre of R21, the pus of a purulent focus creeps into the lumen of the central artery (arrow) and into the lumen of a sinusoid. The artery is relaxed and contains only few maternal erythrocytes, indicating that its connection to the maternal circulation is lost. The homogeneously apoptotic placental area is covered towards the uterine lumen with purulent debris. Extravasal embryonic blood cells and maternal neutrophils populate the fading and disintegrating tissue (Additional file 1, Slide 31A). The purulent focus in the decidua contains MPO7 positive maternal neutrophils (Additional file 1, Slide 31B).

### Placenta with incipient destruction (R22)

The placenta of R22 is better preserved than R21 (Additional file 1, Slide 32). In the adhering remnants of the foetal membranes a large purulent focus with maternal neutrophils marks the former connection to the embryo. The structures of the yolk sac placenta are still visible (Additional file 1, Slide 33A). The well-preserved Reichert membrane is the border between maternal blood compartment and detached embryonic yolk sac layers. Maternal neutrophils invade the maternal decidua capsularis. Clusters of neutrophils have accumulated in the maternal blood lacunae covering the outer surface of the Reichert membrane. The inner layer of the yolk sac (yolk sac proper) forms loose irregular folds (Additional file 1, Slide 34A). The high columnar epithelial cells resemble principal cells of the small intestine indicating the function of absorption. The yolk sac epithelium shows no signs of degeneration. Likewise, the vascular layer of the yolk sac mesoderm is still intact. Attached to the epithelium is a group of transformed embryonic blood cells with MPO7 reactivity. The structure of the murine labyrinth placenta is still preserved (Additional file 1, Slide 34B). A trophoblast layer separates maternal sinusoids with maternal erythrocytes from embryonic capillaries with embryonic erythrocytes. Signs of degeneration are MPO7 containing granules in the cytoplasm of the trophoblast cells and invasion of the tissue by maternal neutrophils, some of which are MPO7 positive.

At the border between decidua basalis and placental tissue a sickle shaped area of tissue destruction and liquefaction has developed (Additional file 1, Slide 35A), which at the decidual side is characterized by foam cells as described in the early resorption R1. F4/80 immunoreactivity of the foam cells (Additional file 1, Slide 35B) indicates that the foam cells are derived from macrophages. The original F4/80 positive macrophage population in the muscle layer is visible in the same section.

The embryo proper of the resorptions still visible by US, was not found in the histological sections. Except for yolk sac, amnion and umbilical vessels no embryonic tissue was preserved. An explanation is that the embryonic tissues were already in an advanced state of dissolution, so that by histology only the non-soluble remnants trapped between relatively more intact structures, were picked up. The yolk sac forms at this developmental stage the outer shell of the embryo. The well-preserved state of yolk sac and yolk sac placenta indicates that they are the last structures to degenerate.

## Discussion

The reproductive strategy of mammals includes the spontaneous resorption of impaired or otherwise non-viable implantations. Up to now it was impossible to foresee and to pick up spontaneous resorptions, which in the majority of cases occur shortly after implantation. We have overcome this problem by daily screening of normal pregnant mice with high resolution US as described in our previous study [15].

In the present study we show that spontaneous embryo resorption is initiated by endogenous apoptosis of the embryo proper, which proceeds autonomously without maternal interference. Only when the foetal-maternal border breaks down and the innate maternal immune system comes in contact with apoptotic embryonic tissues, the mother mounts a sterile unspecific inflammation and rapidly removes the embryonic remnants. The maternal reaction corresponds to the removal of a foreign body via formation of a purulent focus by accumulation of granulocytes.

In early pregnancy stages it was impossible to detect the failing implantations when the embryo proper is still present. A more detailed analysis of the resorption process was possible in later stages. The late resorptions fall into the period from turning of the embryo at day 8 to development of the chorioallantois placenta at day 10 and coincide with a functioning yolk sac placenta. In the late resorptions we observed apoptosis of the embryo proper without participation of maternal immune cells followed by rupture of the embryonic vesicle and abortion of the embryo proper into the uterine lumen. Rupture of the embryonic vesicle and abortion were accompanied by maternal haemorrhage and massive invasion of maternal neutrophils. Failure of lacunar trophoblast and dissolution of the decidua capsularis in the resorptions were clearly discernible from physiological involution of these structures in the normal littermates at days 10 and 11.

### Sterile inflammation

Conventional purulent inflammation implies the focal dissolution of tissue by neutrophil granulocytes in the course of a bacterial infection or foreign body removal. Neutrophil granulocyte invasion and formation of a purulent focus are the pivotal reactions of the innate immune system. Components of disintegrating bacteria such as ATP, DNA and membrane components trigger the attraction and accumulation of neutrophils [18]. Chemotactic cytokines are released directly or by mediation through mast cells and macrophages. Sterile inflammation designates a reaction of the innate immune system without bacteria and occurs in pathological conditions after ischemia reperfusion injury such as heart infarction [﻿16﻿] and acute kidney disease [19] and also in eclampsia [17]. The trigger in these cases is necrosis or interrupted apoptosis passing into necrosis by breakdown of cellular membranes.

We assume that the first step in spontaneous resorption is endogenous embryonic apoptosis. Only when embryo-maternal border and embryonic integrity break down, a damage associated molecular pattern (DAMP, [20]) develops and signals from the disintegrating embryo reach maternal tissue. The mother mounts a rapid sterile inflammatory response. Throughout the resorption process, specific maternal immune cells are absent from embryonic tissues and do not accumulate at the embryo-maternal border.

In the normal littermates, we observed minor forms of unspecific maternal sterile inflammation with invasion of neutrophils in the course of physiological involution of the decidua capsularis. In the resorptions with abortion the decidua capsularis ruptures. In the rupture zone trophoblast cells degenerate possibly triggering the massive neutrophil invasion with haemorrhagic insudation in the overlaying decidua capsularis. These observations indicate that a minor sterile inflammation in the maternal tissue during physiological regression of the decidua capsularis becomes prominent in spontaneous resorption with abortion.

### Embryonic apoptosis

Normal embryonic development requires a steady cellular turnover including apoptosis and cell removal [21]. In embryonic apoptosis, embryonic immune cells fulfil the macrophage function [22, 23], In spontaneous resorption embryonic apoptosis expands over the whole embryo. The self-destruction of the embryo takes place without any participation of maternal tissues or maternal immune cells, as long as the embryonic circulation is intact. This is particularly evident in the entirely apoptotic embryo R15 (Composite 3), in which the heart was still beating two days after detection of developmental failure. Only after breakdown of the embryonic circulation the remnants of the embryonic tissue are removed by maternal sterile inflammation with purulent liquefaction, abortion into the uterine lumen and resorption via the uterine epithelium.

For visualization of apoptosis we used caspase 3 immunohistochemistry. As executioner protease, caspase 3 marks the endpoint of apoptotic cell death [24]. The littermate embryos exhibited physiological caspase 3 positive apoptosis in known locations such as remodelling of tail somites, formation of digits and remodelling of pharyngeal clefts. In the embryos under resorption in the early stage of apoptosis, extended areas, not known for physiological apoptosis expressed caspase 3. They appeared as enlarged foci of physiological apoptosis indicating the same cellular mechanisms but now out of control. The same was true for the spotted caspase 3 expressions in the normal placenta during steady transformation of the decidua, which became large confluent areas of tissue liquefaction.

In the early stages of apoptosis, before expression of caspase 3, the extracellular spaces were extended, and high columnar epithelia showed apical blebs. Apical mitotic figures, regularly present in the littermates, were absent in embryos under resorption. The few retained mitotic figures displayed blurred chromosomes corresponding to an early stage of apoptosis as described by Leidenfrost, et al. [25] . In general disappearance and dissolution of the apoptotic embryo occurs very rapid. Therefore, most of the resorptions were in the final “open cup” stage with a wound area opening into the uterine lumen with no embryo left.

We observed two types of apoptosis, caspase 3 positive apoptosis in embryo proper and placenta, and caspase 3 negative apoptosis in trophoblast cells of the rupturing lacunar trophoblast. This observation may have a major bearing in the light of molecular apoptosis pathways as reviewed by Zhang, et al. [26]. Caspase 3 positive embryonic apoptosis is programmed cell death inside an intact cell membrane. The dying cells expose “eat me signals” (e.g. via the phosphatidylserine flip) on their surface, which attract embryonic immune cells for their disposal [27]. Only in the final state the cell membrane breaks down, and damage associated pattern (DAMP) leads to formation of a purulent focus with maternal neutrophils. Caspase 3 negative apoptosis of trophoblast cells may correspond to primary necroptosis or may be a form of pyroptosis with inflammosome assembly and secretion of IL-1β/IL-18.

In case of pyroptosis of the lacunar trophoblast this would indicate a decisive role of trophoblast cells at the fetal-maternal border in triggering embryo resorption.

### Transformed embryonic blood cells

In the mouse embryo, the first immunological markers for macrophage and B-cell potential of the specific immune system arise around day 10 of murine development [28]. Detection of a phagocyte-like transformation of embryonic haematoblasts in the resorptions demonstrates the presence of an innate unspecific immune system in the early embryo.

The changes in morphology of the blood cells were a constant feature in all embryos under resorption and were regularly also observed in the large allantoic and vitelline vessels. Some of the transformed blood cells in the apoptotic embryonic tissues were MPO7 positive. MPO7 is a marker of finally activated neutrophils, which is released in the last stage of inflammation [29]. The observation of MPO7 positive embryonic blood cells indicates a function in the process of inflammation, as known from adult neutrophils.

### Role of lacunar trophoblast

We designate the trophoblast layer between decidua capsularis and Reichert membrane as lacunar trophoblast since it resembles the lacunar stage of the human syncytiotrophoblast. The lacunar trophoblast in the mouse is a constitutive element of the yolk sac placenta, whereas the lacunae of syncytiotrophoblast in the human represent the early stage of the chorioallantois placenta.

The lacunar trophoblast of the yolk sac placenta in the mouse develops from the mural trophoblast of the blastocyst [30]. The non-syncytial trophoblast cells are polyploid and finally form a spongy network of lacunae filled with maternal blood supplied by maternal sinusoids of the decidua capsularis. In embryos under resorption the lacunae are more extended than in normal littermates, probably due to the reduced tension in the embryonic cavities.

Degeneration of the lacunar trophoblast was characterized by extended lacunae with subsequent formation of free maternal blood lakes between Reichert membrane and decidua capsularis. The blood in the trophoblast lacunae did not aggregate, not even in the embryos under abortion. This was in sharp contrast to the maternal blood in the extravascular space of yolk sac cavity and uterine lumen, which as maternal haemorrhage was instrumental in expulsion of the apoptotic embryo. The trophoblast produces anticoagulant factors. Knock-out mice for the thrombospondin gene undergo resorption abortion around day 9.5 [31, 32].

In the course of abortion, the lacunar trophoblast forms a sliding surface between embryonic and maternal tissue. The dead embryo within its membranes is separated from its original attachment site at the mesometrial decidua by a zone of degeneration in the lacunar trophoblast enabling expulsion of the embryo into the uterine lumen. In the normal littermates, involution of the lacunar trophoblast takes place at day 11. In normal development extension of the yolk scathe lacunar trophoblast is stretched out to a finally discontinuous single cell layer between Reichert membrane and remnants of the decidua capsularis. In normal development and in embryos under resorption, cell death in the non-proliferating trophoblast lacunar trophoblast takes place without caspase 3 expression and seems to represent a special variety of necrobiosis.

In some resorptions, the trophoblast even seems to exhibit further growth and self-organization after the embryo proper has already disappeared. An array of lacunar trophoblast was found at the site of the placenta anlage. This reminds of pathological trophoblast growth in hydatidiform moles, a pregnancy where normal embryonic or foetal elements are lost, and of choriocarcinoma [33].

### Decidua capsularis

The decidua capsularis in the mouse is not homologous to the decidua capsularis in the human. This is due to the antimesometrial implantation and inversion of germ layers in the mouse. In the mouse the antimesometrial and mesometrial decidua fuse and temporarily obdurate the primary uterine lumen. Formation of a secondary uterine lumen separates the antimesometrial decidua from the antimesometrial uterine wall. As the embryonic vesicle expands, the antimesometrial decidua is compressed to a dense layer of tissue, which surrounds the embryonic vesicle and therefore is called decidua capsularis. The decidua capsularis consists not only of decidua (sensu stricto) but also of the sinusoidal and basal layers of the antimesometrial endometrium.

Physiological involution of the decidua capsularis in the mouse takes place between day 10 and 11 [30, 34]. In our study of normal littermates we observed that it is accompanied by moderate leucocyte invasion in the contact zone with the epithelium of the secondary uterine lumen. In the resorptions with abortion the decidua capsularis ruptures to release the apoptotic embryo into the uterine lumen. Dissolution in the rupture zone goes along with massive invasion of maternal leucocytes and extravasation of maternal blood.

### Resorption of maternal tissue via foam cells

In normal implantation sites and in implantation sites under resorption, F4/80 positive, spindle shaped macrophages were mainly present in the muscular layer at the mesometrial root. Our immunohistochemistry indicates, that the macrophages transform gradually into foam cells characterized by densely packed intracytoplasmatic vacuoles. During transformation, the F4/80 immunoreactivity translocates to the outer cytoplasm, leaving the central vacuolated zone unstained. In the resorption sites and in the normal littermates, the foam cells show B220 immunoreactivity. In normal development small apoptotic spots in conjunction with macrophages characterize the continuous remodelling of the decidua [35]. Macrophages exhibiting foam cell morphology are related to vascular remodelling of the spiral arteries [36] and have been described in the human endometrium [37]. In pathological conditions, such as endometrial hyperplasia and preeclampsia, the appearance of foam cells is more pronounced [37–39]. During resorption, the apoptotic processes are enhanced and finally enter into a state of tissue liquefaction, particularly around the spiral arteries. Our histological and immunohistochemical observations show that in spontaneous resorption foam cells derived from uterine macrophages, perform the dissolution of the mesometrial decidua.

## Conclusion

Spontaneous resorption is initiated by endogenous apoptosis of the embryo proper, which proceeds autonomously without maternal interference. During embryonic apoptosis caspase 3 is expressed and transformed embryonic cells fulfil macrophage and neutrophil like functions with expression of neutrophil-specific MPO7. Only when the embryo-maternal border breaks down the mother mounts a sterile unspecific inflammation. The embryonic vesicle ruptures and the apoptotic embryo is aborted into the uterine lumen and rapidly resorbed. The maternal reaction corresponds to the removal of a foreign body via formation of a purulent focus by accumulation of granulocytes. The maternal part of the implantation site dissolves by apoptotic liquefaction and transformation of F4/80 positive macrophages into foam cells.

## Methods

### Animals

Mice from the C57BL/6 strain (30 females and 2 males) were obtained from Harlan Laboratories, Rossdorf Germany and held under conditions described in a previous study [15]. For breeding purposes, the animals were kept in groups of three females and one male for three days without any hormonal treatment under a 12 h dark and light cycle. During this period, females were checked daily for a mating plug to confirm pregnancy. Additionally, high resolution ultrasound was performed four days after establishment of breeding groups. Prior tissue sampling, animals were deeply anaesthetised by isoflurane at a flow rate of 5% (oxygen flow 1 l/min) delivered via face mask and killed via cervical dislocation.

All animal experiments complied with the institutional and governmental regulations (Tierschutz-Versuchstierordnung) and were approved by the State Office of Health and Social Affairs, Berlin (letter 03.11.2010) in accordance with the German law of animal welfare.

### High-resolution ultrasound

Resorption sites were detected by ultra-high frequency ultrasound (30–70 MHz), so called ultrasound biomicroscopy (UBM), as described in the previous study [15].

### Staining protocols

Paraffin sections (10 μm) were dewaxed and stained histochemically with hematoxylin and eosin (HE). For immunohistochemistry, the paraffin sections were dewaxed and subjected to a heat-induced epitope retrieval step except for sections for prior incubation with anti-B220 (clone RA3-6B2, BD Bioscience, 1:400). Primary antibodies against cleaved caspase-3 (Asp175, Cell Signaling, USA, 1:400) and MPO7 (polyclonal rabbit, Dako, code A0398, 1:1000) were used. This was followed by incubation with biotinylated secondary antibodies (Dianova). For detection, alkaline phosphatase-labelled streptavidin and chromogen RED (both Dako) were employed. For detection of macrophages, sections were subjected to protein-induced epitope retrieval employing protease (Sigma) prior to incubation with anti-F4/80 (clone BM8, eBioscience, 1:800). This was followed by incubation with biotinylated rabbit anti-rat (Dako) secondary antibody. Biotin was detected using alkaline phosphatase-labelled streptavidin (Dako). For visualization of alkaline phosphatase, chromogen RED (Dako) was used. Nuclei were counterstained with hematoxylin (Merck). Negative controls were performed by omitting the primary antibody.

### Microscopy and preparation of composites

Pictures were taken with a Zeiss Axiostar microscope equipped with an AxioCam MRC camera and Axiovision software. Brightness, contrast, and colour balance were adjusted only for whole images using the levers in the axiovision software. Feature within an image were not enhanced or otherwise altered.

For six representative specimens, interactive schematic drawings (composites) were prepared with the Corel Draw X7 software. Partial images of central histological sections (objective 5x) were composed and adjusted by digital image processing. Structures such as uterine muscular layers, uterine epithelium, decidua basalis, and extraembryonic membranes were segmented, converted to vector graphics, marked with colors, and assigned to specific layers.

## Supplementary information

**Additional file 1.** Detailed observations.

**Additional file 2.** Interactive composites.

## Data Availability

The original histological samples and derived photographs created and analysed during the current study are available from the corresponding author upon request.

## Abbreviations

li: liquefaction
mh: maternal haemorrhage
MPO: myeloperoxidase
n: neutrophil granuloctes
pu: purulent focus
Rm: Reichert membrane
si: sinusoids
tr: trophoblast
ul: uterine lumen
US: ultrasound
ys: yolk sac
