# Supplementary material for: Spontaneous embryo resorption in the mouse is triggered by embryonic apoptosis followed by rapid removal via maternal sterile purulent inflammation
Source: BMC Dev Biol. 2020 Jan 9;20:1. doi: 10.1186/s12861-019-0201-0 (PMC6953269; doi:10.1186/s12861-019-0201-0)
Supplement: Supplementary file 1 — Additional file 1. Detailed observations. [file 12861_2019_201_MOESM1_ESM.pdf]

Barbara Drews, Luis Flores Landaverde, Anja Kühl, Ulrich Drews

Additional file1: Detailed Observations

LIST OF SLIDES

Slide 2: Composite 1 (R2) Early resorption with purulent focus and self-organizing trophoblast 2
Slide 3: (Inset in s2) Trophoblast array, haemorrhage, and suppurated focus 3
Slide 4: Composite 2 (R1). Purulent focus and liquefaction of the decidua basalis via foam cells 4
Slide 5: Inset in s4. Purulent focus. 5
Slide 6: Inset in s4. Liquefaction of decidua basalis via foam cells.. 6
Slide 7: Composite 3. The apoptotic embryo in situ. 7
Slide 8: (Inset in s7). Degenerated lacunar trophoblast and intact decidua capsularis. 8
Slide 9 (Inset in s7): Apoptotic tissues and embryonic immune cells. 9
Slide 10: Caspase 3 immunoreactivity. Apoptotic tissues and transformed embryolic haematoblsts . 10
Slide 11 (Inset in s7): Comparison of transformed and normal embryonic blood cells. 11
Slide 12: Myeloperoxidase (MPO7) immunoreactivity in transformed embryonic blood cells 12
Slide 13: Composite 4 (R11): halfway aborted embryo. 13
Slide 14: The rupture site. 14
Slide 15: Overview of section with B220 immunoreactivity. 15
Slide 16: Lower insets of slide 15: Untimely decidualized and degenerated decidua capsularis 16
Slide 17: Upper insets of slide 15: B220 immunoreactivity in foam cells and small lymphocytes 17
Slide 18: caspase 3 immunoreactivity in apoptotic embryo. 18
Slide 19: Inset A of Slide 18. 19
Slide 20: MPO positive maternal neutrophils in the apoptotic embryo 20
Slide 21: F4/80 positive maternal macrophages beneath uterine epithelium. 21
Slide 22 Composite 5 (R13): Resorption with totally aborted embryo and maternal haemorrhage 22
Slide 23: Delamination of embryonic and placental tissue by maternal haemorrhage 23
Slide 24: Insets of 23 A: Rupture zone of maternal and embryonic vessels 24
Slide 25 Mixed blood in chorionic cavity and in apoptotic embryo 25
Slide 26 Composite 6 (R16) The final “empty cup” stage of resorption 26
Slide 27: Maternal lymphocytes and neutrophils in the degenerated lacunar trophoblast 27
Slide 28: Regression of decidua basalis and trophoblast in the placental area 28
Slide 29: B220 Immunoreactivity in the “final cup” stage 29
Slide 30: Overview of decaying placenta with caspase 3 immunoreactivity (R21) 30
Slide 31: MPO immunoreactivity in placental and decidual areas (R21) 31
Slide 32: Overview of placenta with embryonic remnants, MPO immunoreactivity (R22) 32
Slide 33: Insets of slide 32 (R22) 33
Slide 34: Yolk sac epithelium and labyrinth placenta (R22) 34
Slide 35: F4/80 immunoreactivity in macrophages and foam cells (R22) 35

Composite 1

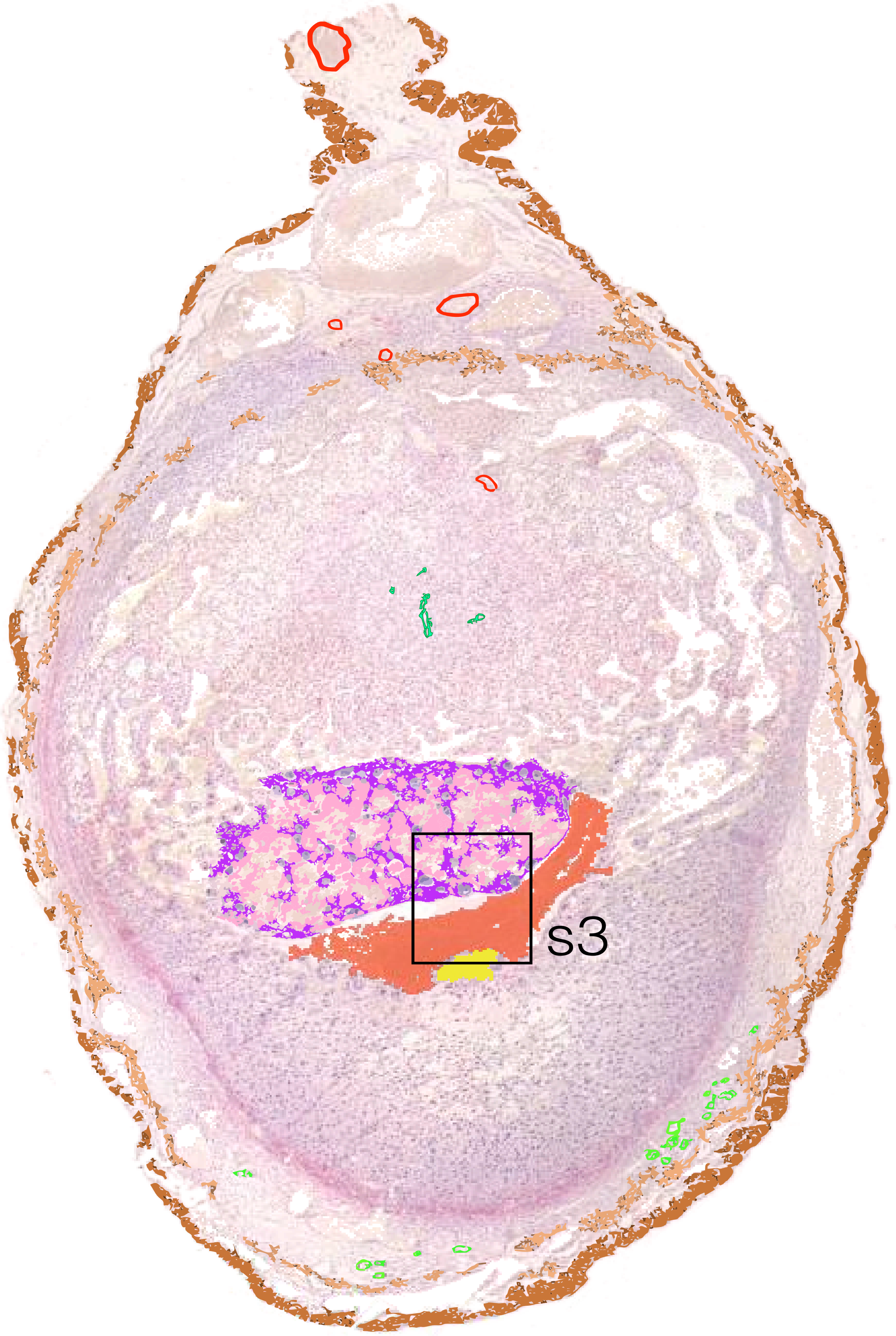

Slide 2: Composite 1 (R2) Early resorption with purulent focus and self-organizing trophoblast

The embryo is replaced by maternal haemorrhage and a suppurated focus. Self-organizing trophoblast seems to escape degeneration. Underlying section with caspase 3 staining. Detection by US: day 7. Histology: day 8. HE

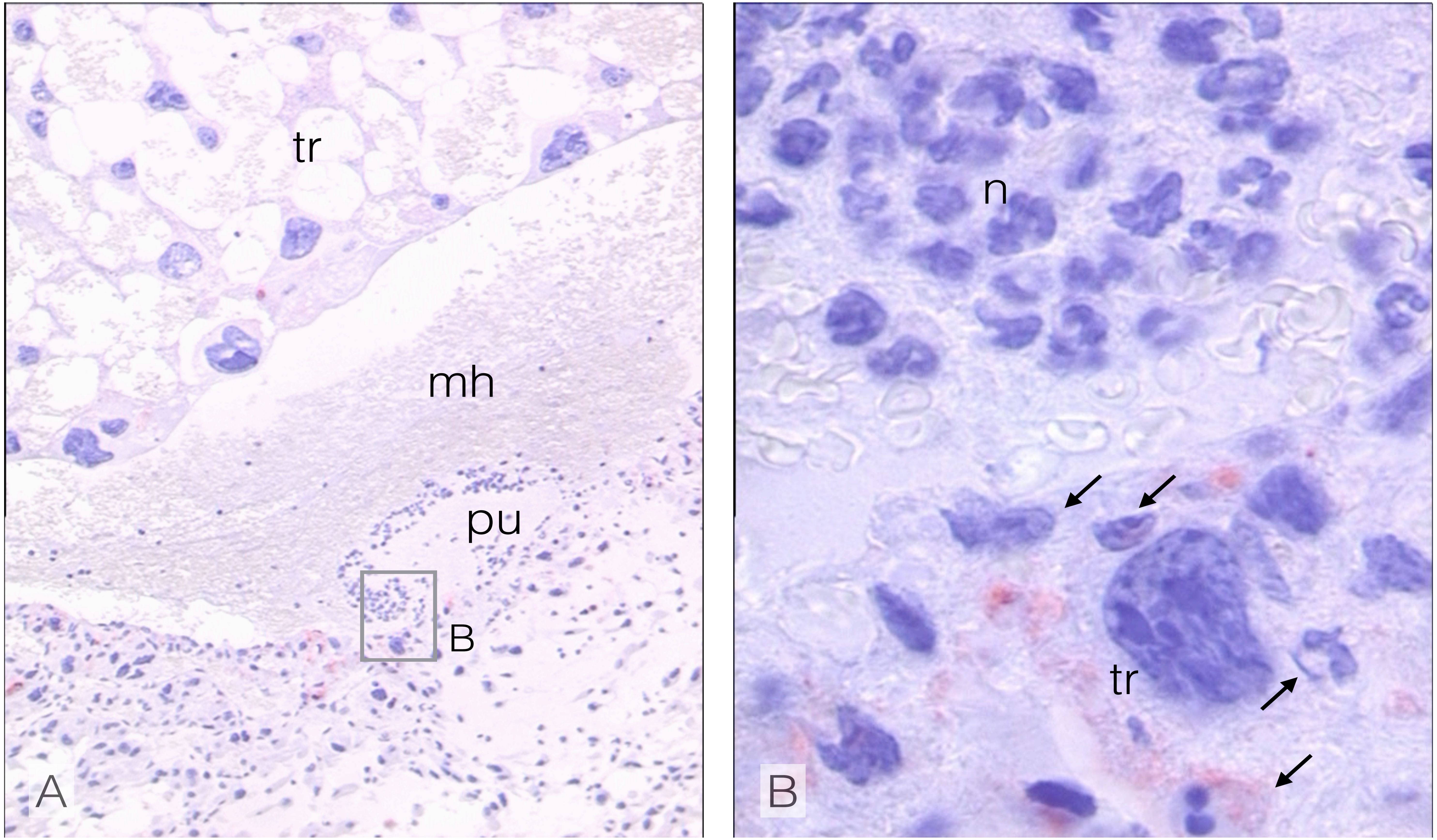

Slide 3 (Inset in s2): Trophoblast array, haemorrhage, and suppurated focus

Higher magnification of underlying section of composite 1. **A** self-organizing trophoblast array (tr), maternal haemorrhage (mh), and purulent focus (pu), 10x. **B** Inset: purulent focus with neutrophil granulocytes (n) and maternal erythrocytes. Trophoblast cell (tr) with faint caspase 3 staining in the cytoplasm, invaded by neutrophils and lymphocytes (arrows). 100x. Caspase 3

Composite 2

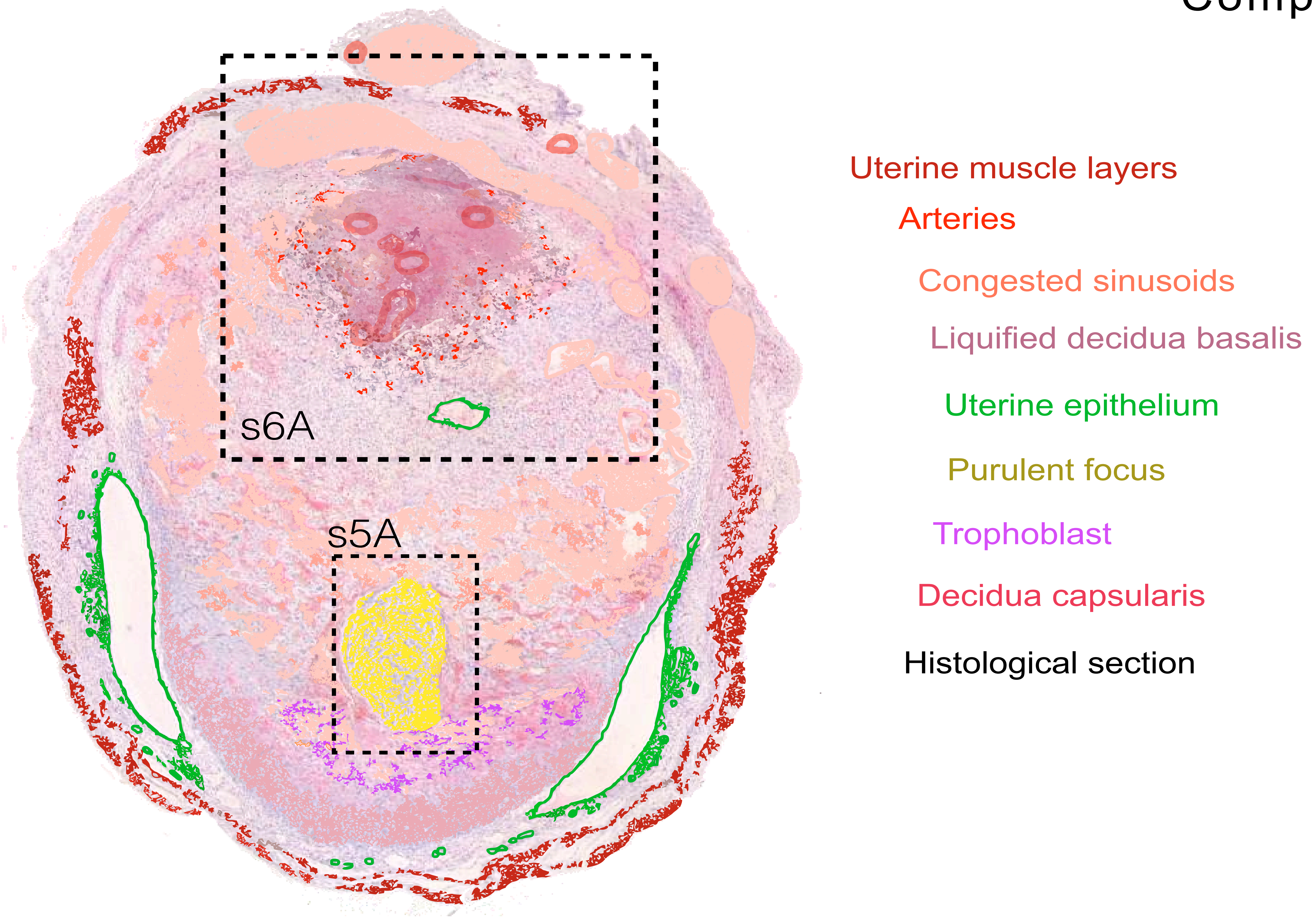

Slide 4: Composite 2 (R1). Purulent focus and liquefaction of the decidua basalis via foam cells

Composite 2 is further developed than Composite 1. The secondary lumen of the uterine canal has formed, the purulent focus is enlarged. In the basal decidua, a second center of tissue liquefaction appears. Detection by US: day 7. Histology: day 8. HE

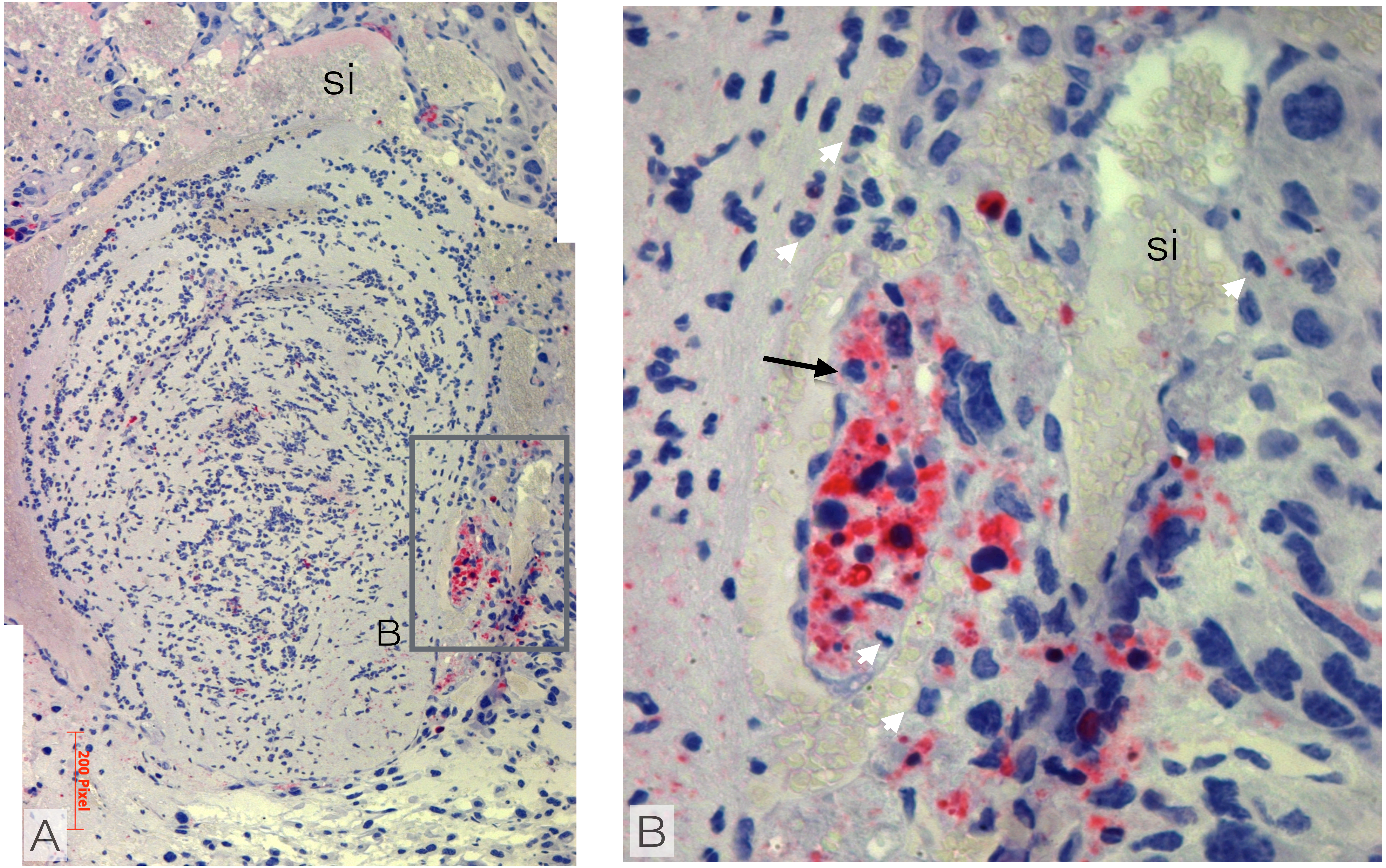

Slide 5: Inset in s4. Purulent focus.

A Purulent focus as indicated in Composite 2. Massive accumulation of maternal neutrophil granulocytes in a gel-like amorphous matrix surrounded by congested maternal sinusoids (si). Caspase 3 immunoreactivity. 20x. **B** Inset: delimited spot of final apoptosis in the sinusoidal decidua with caspase 3 positive foam cells (black arrow), cell detritus, and neutrophil granulocytes (white arrow heads). 40x

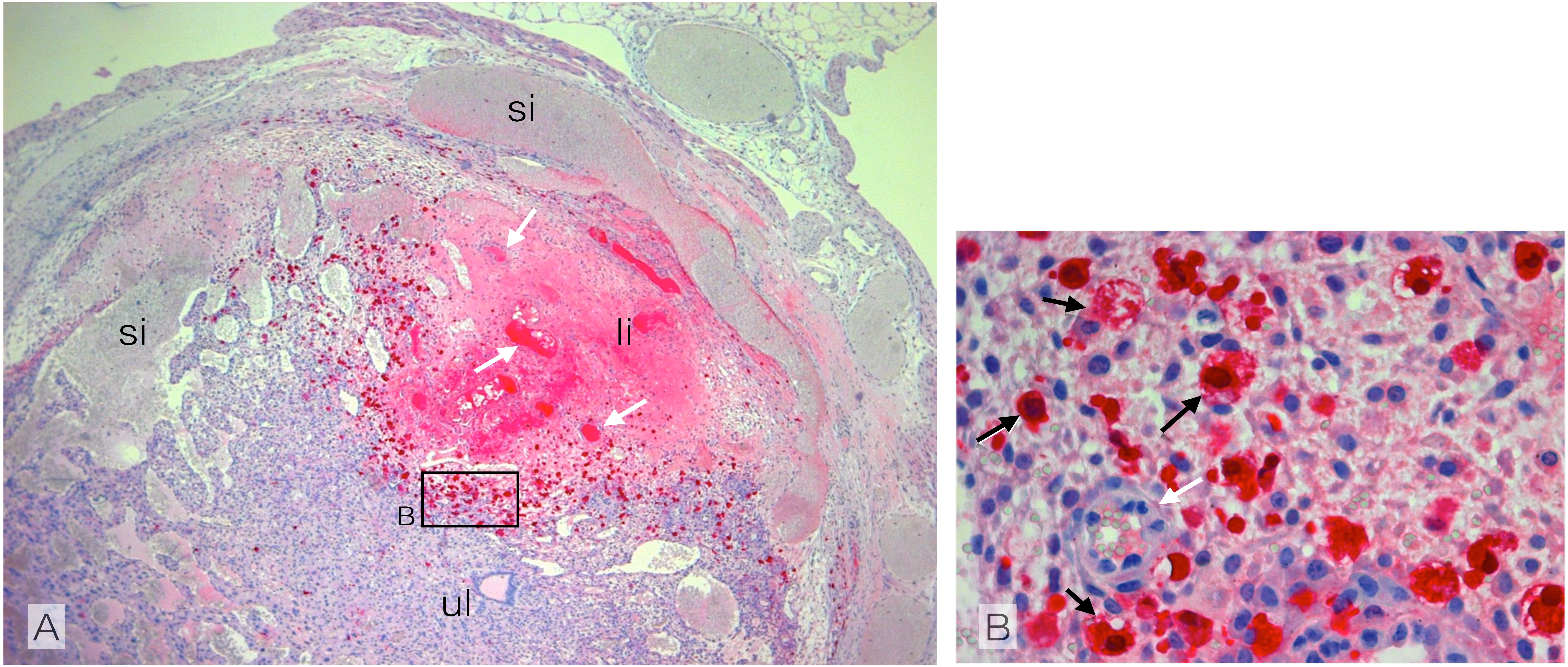

Slide 6: Inset in s4. Liquefaction of decidua basalis via foam cells..

A Centre of liquefaction (li) in the sub-mesometrial decidua surrounding the arteries (white arrows). si congested sinusoids, ul primary uterine lumen. Caspase 3 5x. **B** Inset: Caspase 3 positive foam cells (black arrows), arteries (white arrows).63x.

Composite 3

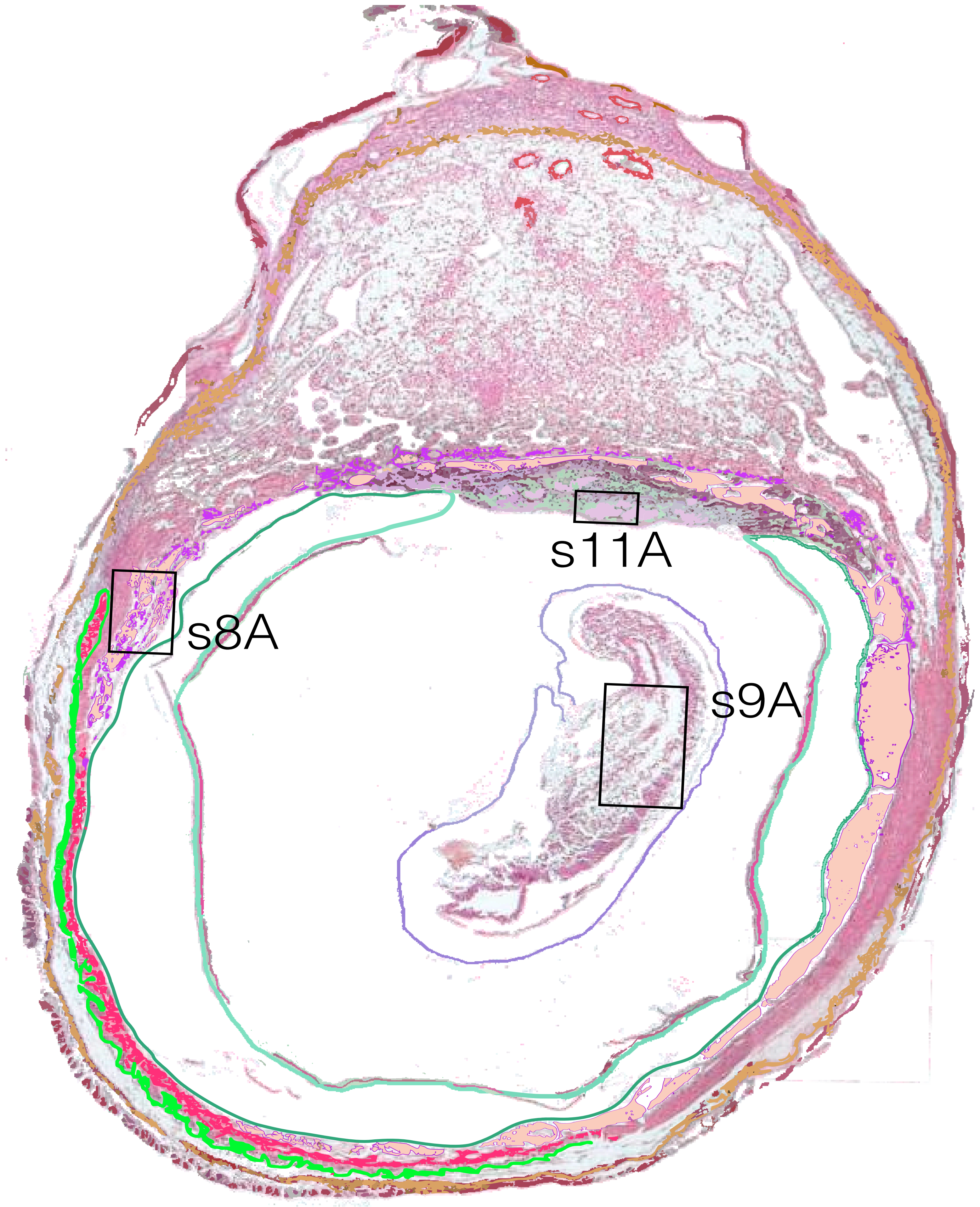

Uterine muscle layers
Arteries
Trophoblast with lacunae
Allantois mesoderm
Amnion
Yolk sac with Reichert membrane
Fibrinoid
Uterine epithelium
Histological section

Slide 7: Composite 3. The apoptotic embryo in situ.

The embryo is enveloped in its membranes. Embryonic tissues are in a state of final apoptosis while the maternal tissue appears still normal. Detection by US: day 9. Histology: day 10. HE

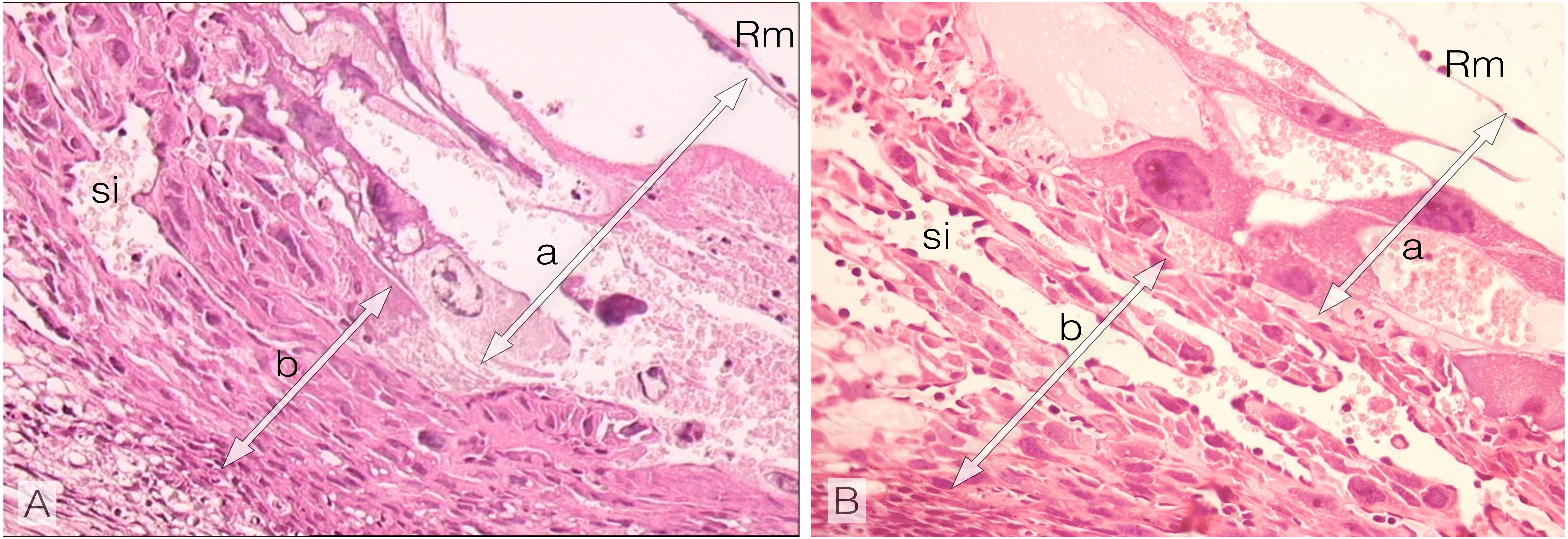

Slide 8: (Inset in s7). Degenerated lacunar trophoblast and intact decidua capsularis.

A Resorption site with intact decidua capsularis and autolytic lacunar trophoblast. HE. 63x **B** Day 9 normal development. HE 63x. Frame A and B (indicated in Slide 7) are located in the yolk sac angle and display the same overall structure. Rm Reichert membrane, si maternal sinusoid, a lacunar trophoblast; b decidua capsularis

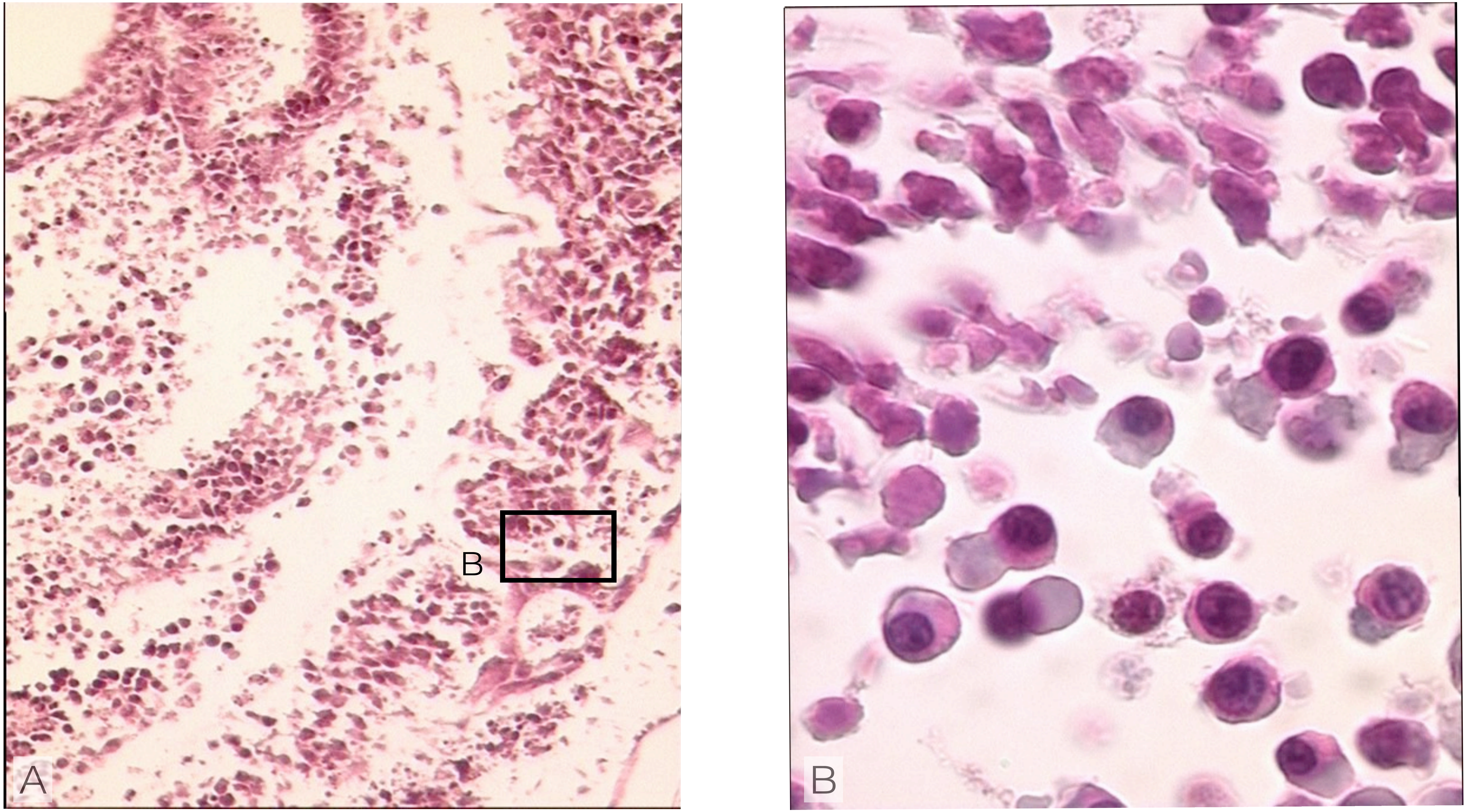

Slide 9 (Inset in s7): Apoptotic tissues and embryonic immune cells.

A Degenerating embryo (detail enlargement of Composite 3, slide 7). Transformed embryonic haematoblasts participate in dissolution of embryonic tissue. HE 10x. **B** Inset: Transformed haematoblasts within cellular debris. 100x

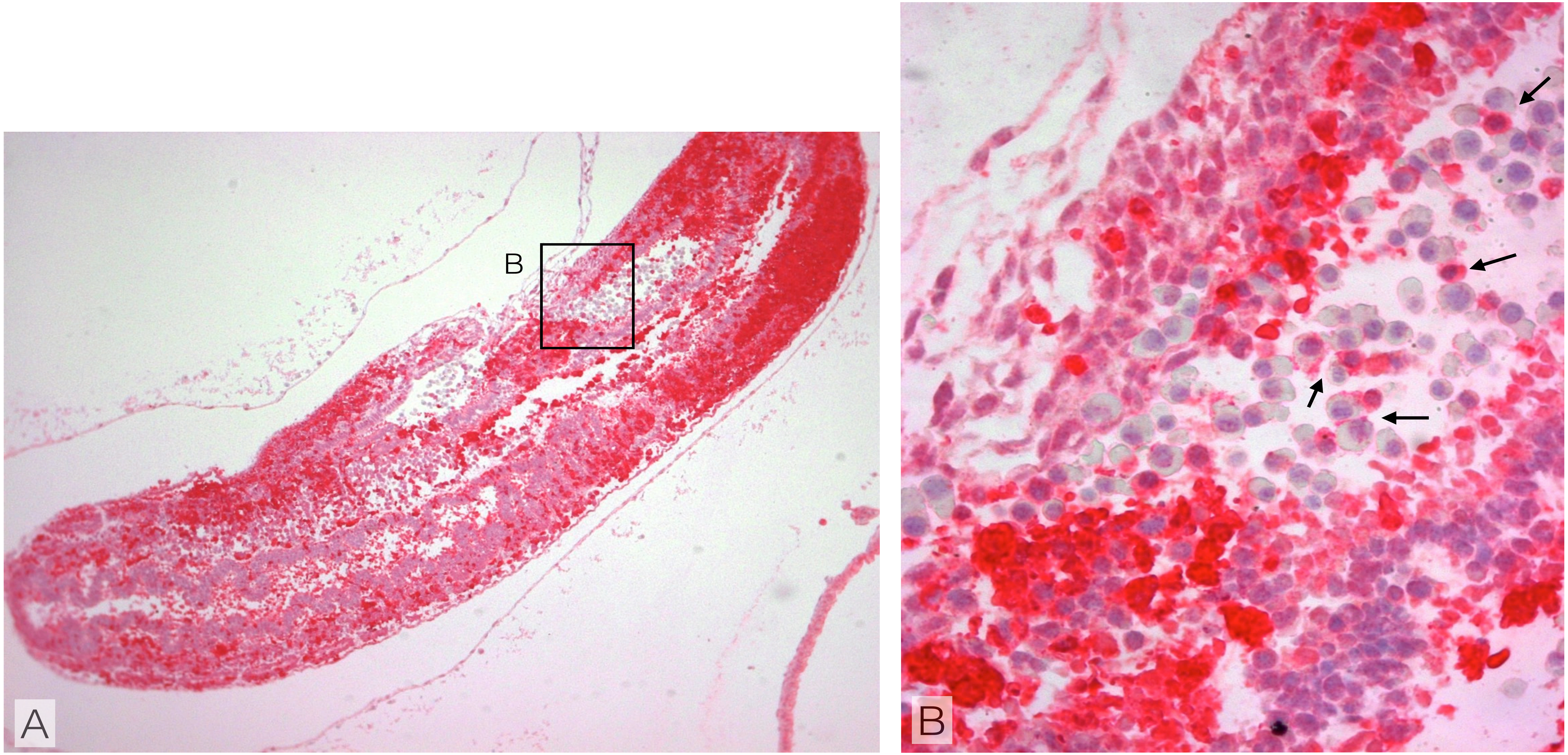

Slide 10: Caspase 3 immunoreactivity. Apoptotic tissues and transformed embryolic haematoblsts .

A In the apoptotic embryo, almost all cells show caspase 3 immunoreactivity. **B** Transformed embryonic haematoblastsare caspase 3 negative and aggregate with caspase 3 positive embryonic cells (black arrows) 100x

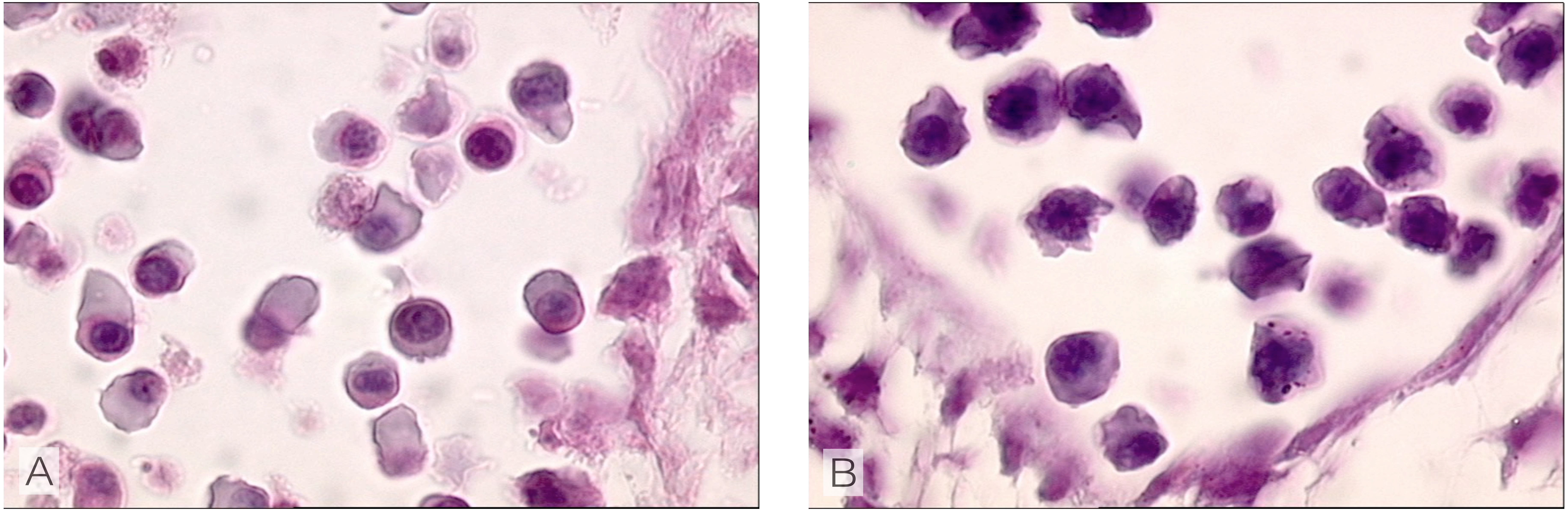

Slide 11 (Inset in s7): Comparison of transformed and normal embryonic blood cells.

A Transformed embryonic haematoblasts with large vacuoles in the allantois mesoderm of R15. 63x **B** Normal embryonic erythrocytes in the allantois at day 9. 63x HE

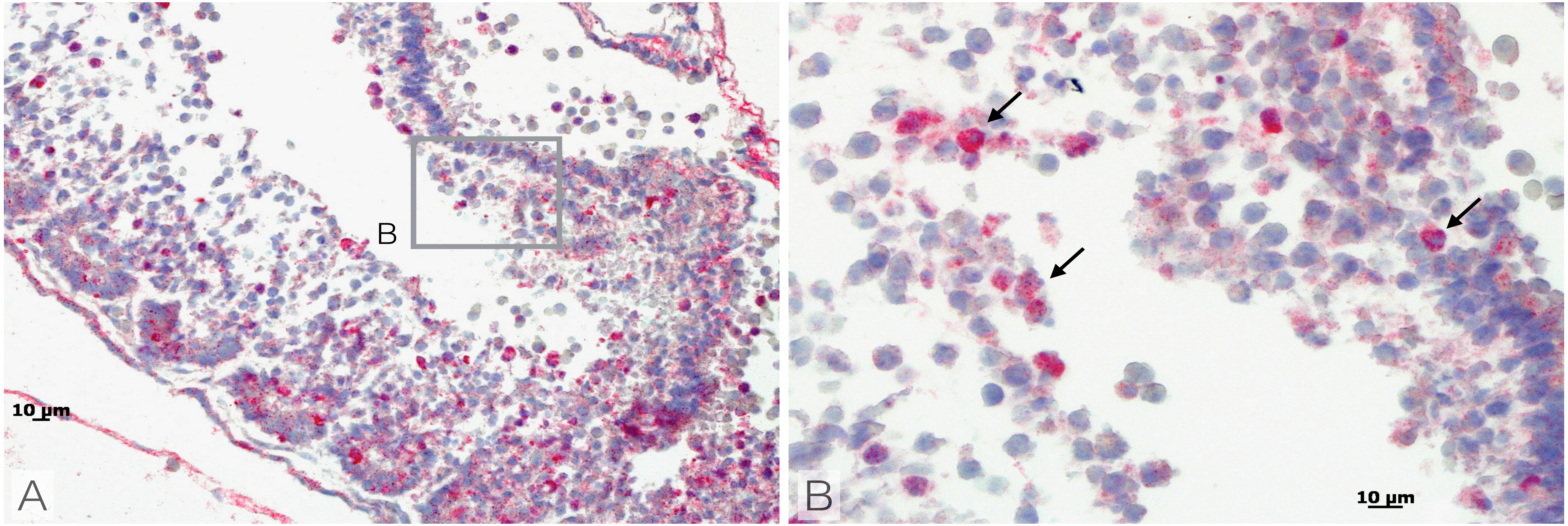

Slide 12: Myeloperoxidase (MPO7) immunoreactivity in transformed embryonic blood cells

A MPO7 reactivity in a subset of transformed embryonic haematoblasts 20x **B** Inset: higher magnification of MPO-7 positive embryonic haematoblasts (black arrows) 40x

Composite 4

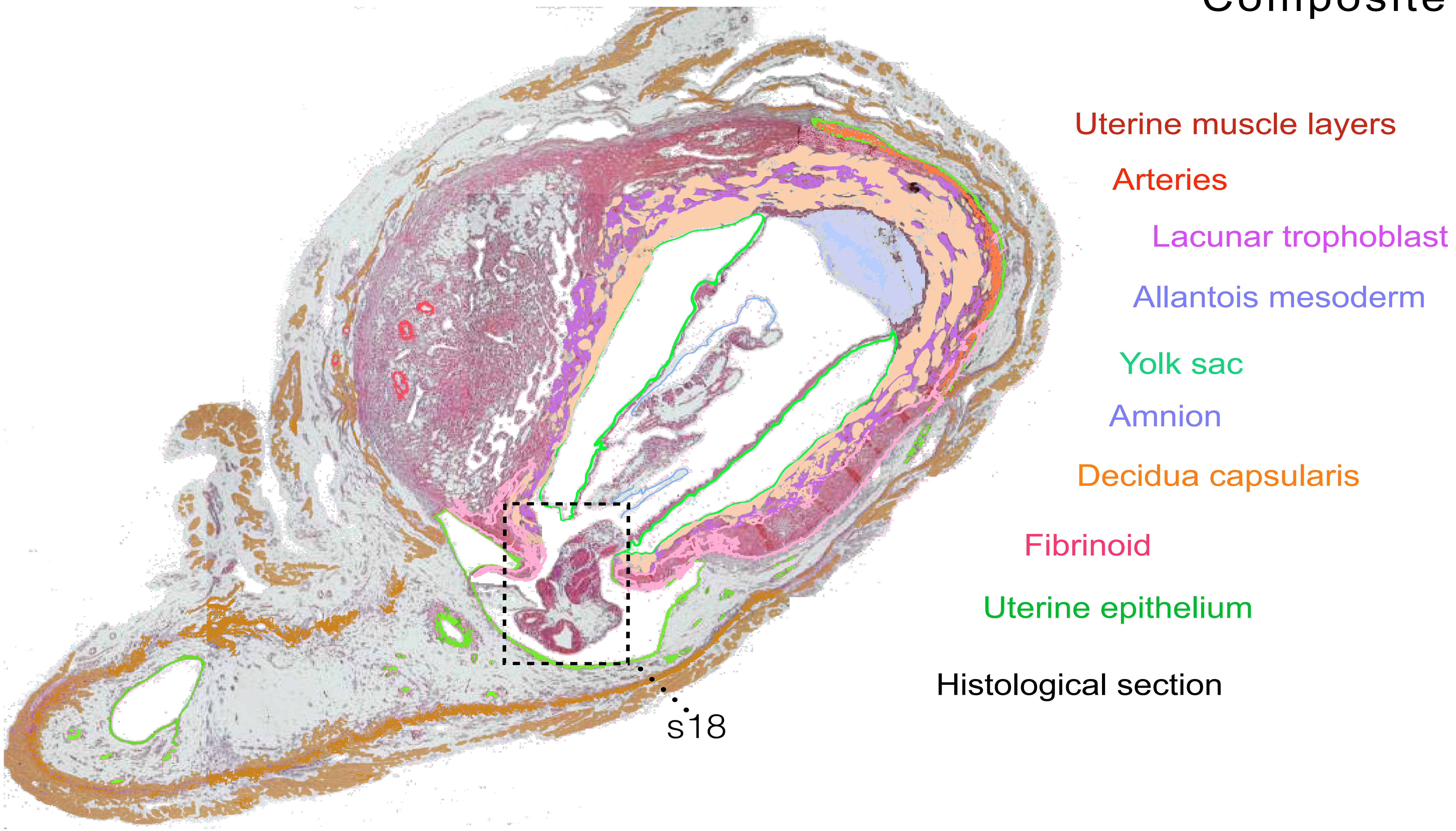

Slide 13: Composite 4 (R11): halfway aborted embryo.

The embryo and its membranes is rotated by about 90° into the longitudinal axis of the uterine lumen and the allantois mesoderm translocated away from the placental area. The ruptured network of the lacunar trophoblast thereby seems to form a sliding surface. The apoptotic embryo is partially expelled into the uterine lumen. Detection by US: day 9. Histology: day 9 HE

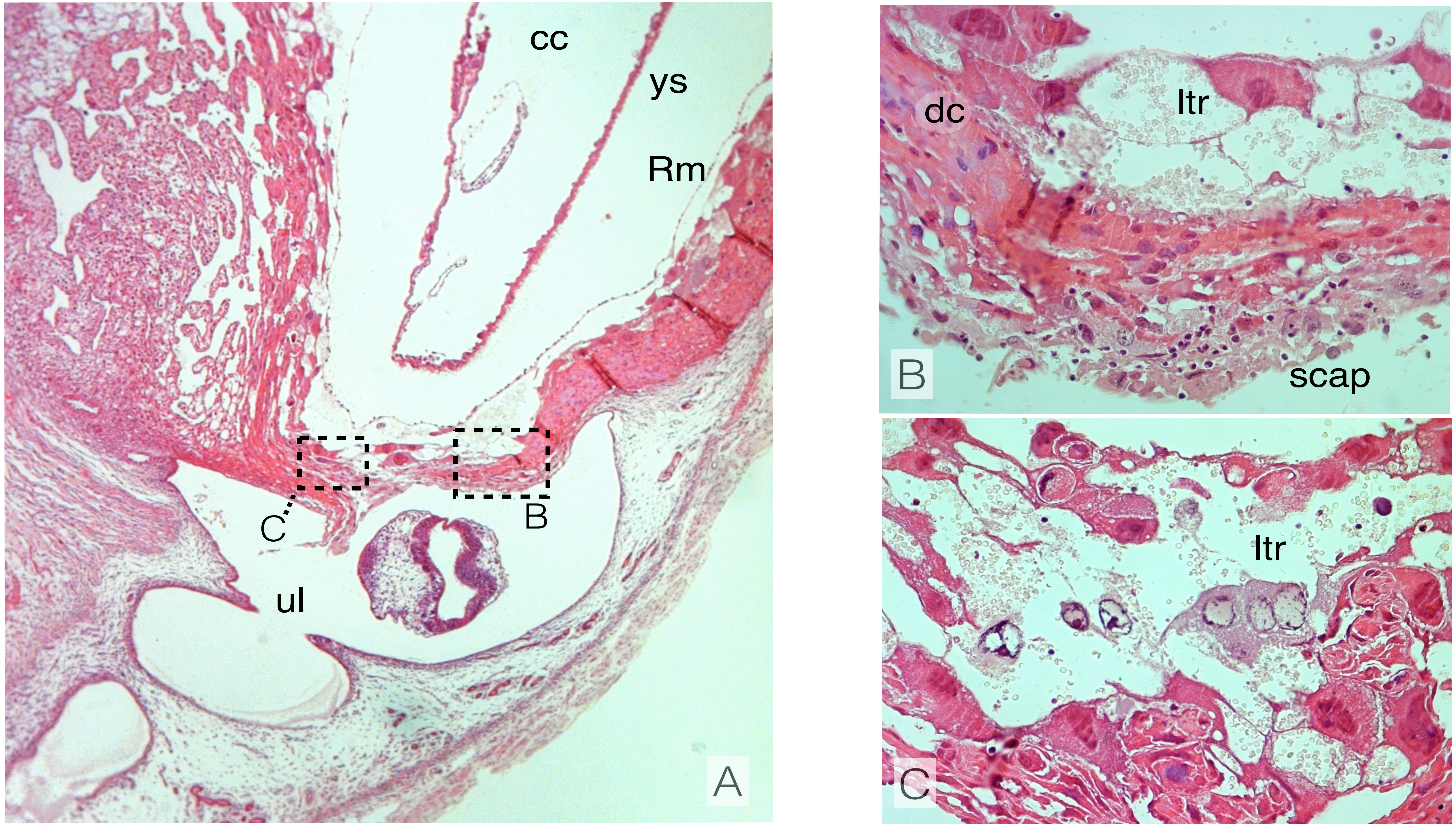

Slide 14: The rupture site.

A Consecutive section of S13 above rupture site. cc chorionic cavity, ys yolk sac, Rm Reichert membrane, ul uterine lumen. HE 5x. **B** Inset:Fibrinoid scap with neutrophils in decidua capsularis (dc) 40x HE. **C** Inset: Nuclear swelling in lacunar trophoblast cells (ltr). 40x HE

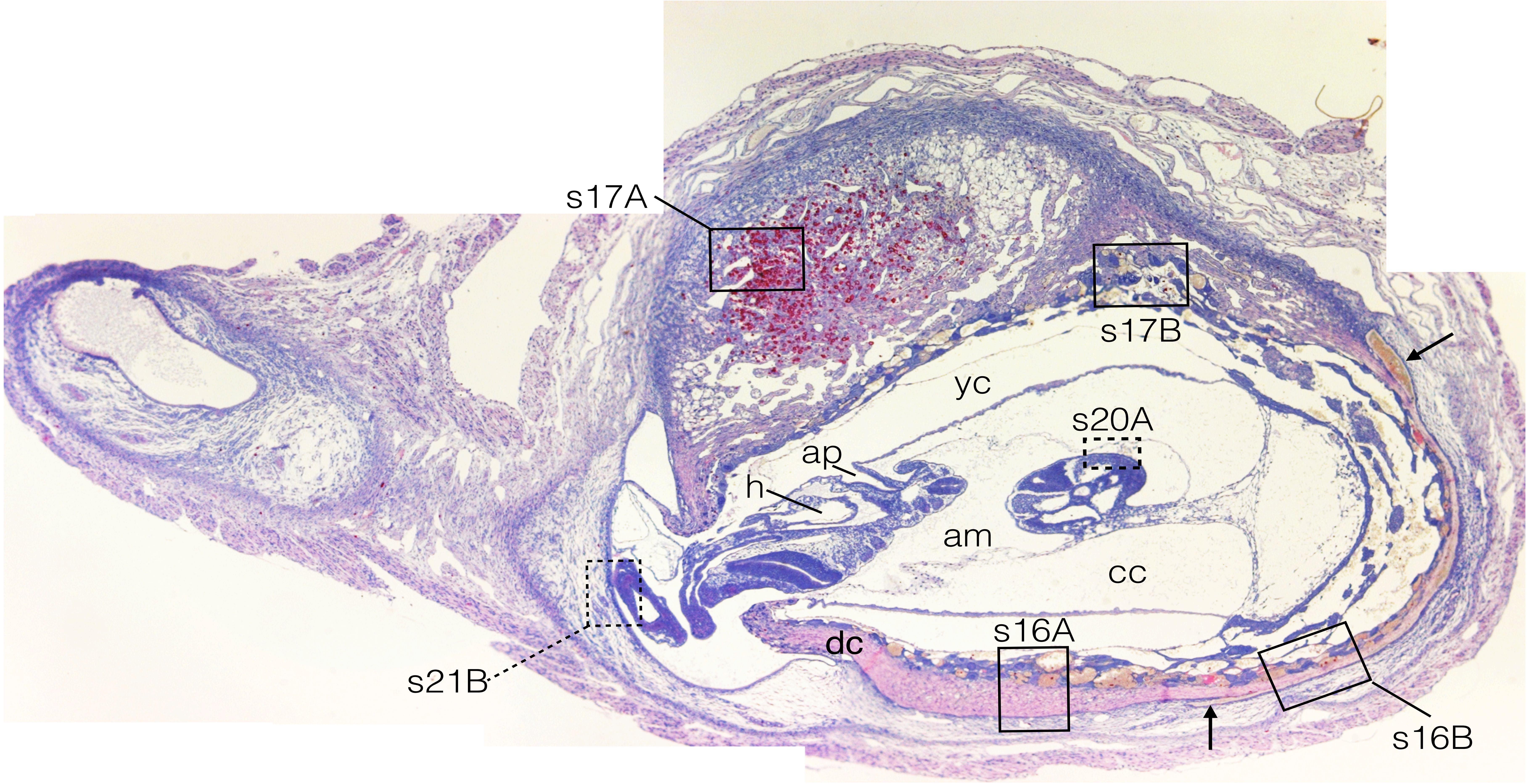

Slide 15: Overview of section with B220 immunoreactivity.

Merged images of whole section HE 5x. h heart; ap anterior intestinal portal; yc yolk sac cavity; am amniotic cavity; cc chorionic cavity; arrows: uterine lumen with coagulated maternal blood

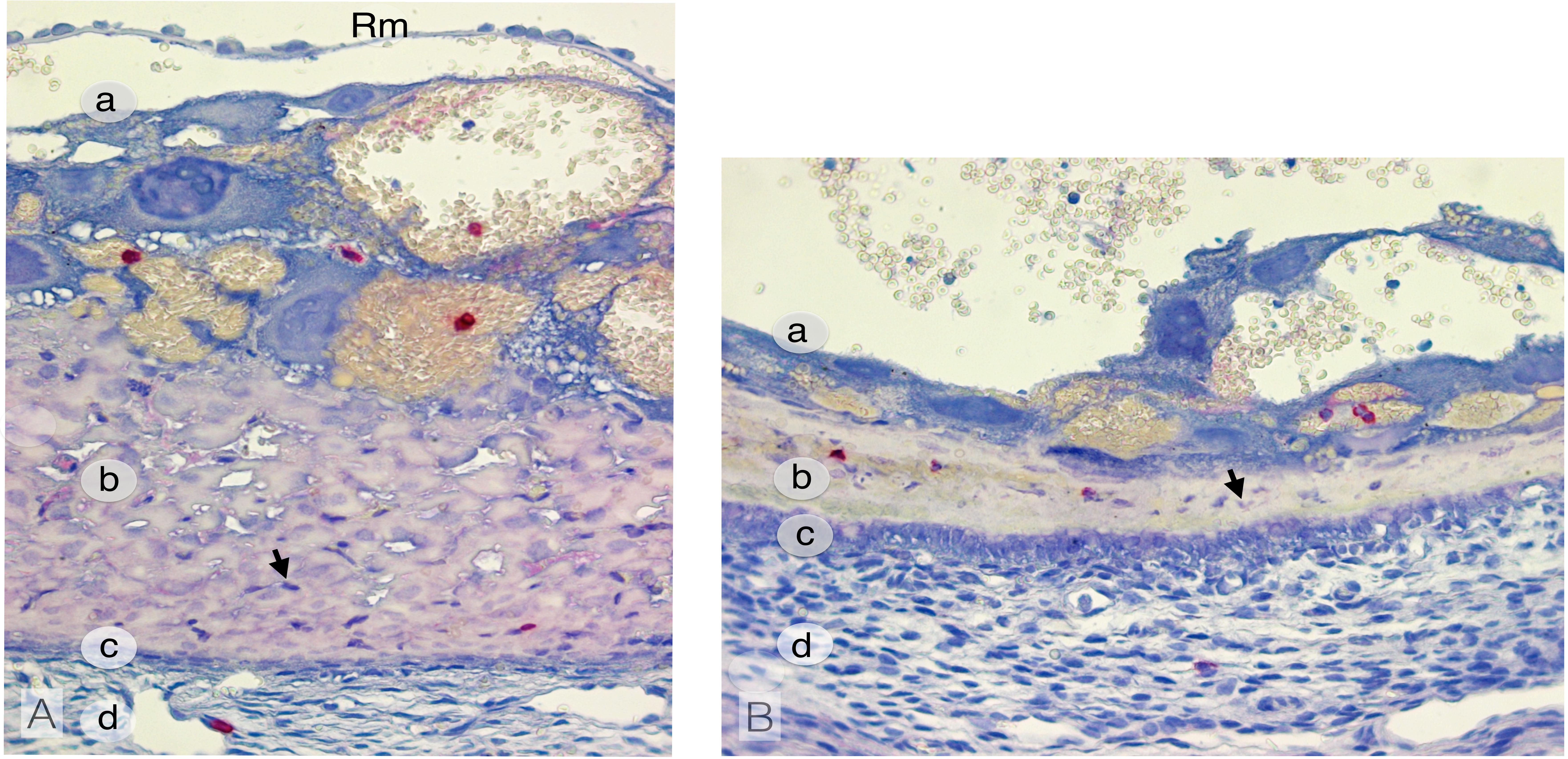

Slide 16: Lower insets of slide 15: Untimely decidualized and degenerated decidua capsularis

A Decidualized layer of antimesometrial decidua with B220 positive small lymphocytes in the maternal blood of the trophoblast lacunae; 40x **B** Fibrinoid remnant of degenerated decidua capsularis with B220 positive maternal small lymphocytes and decomposing neutrophils (arrowhead); 40x Rm Reichert membrane; a lacunar trophoblast; b decidua capsularis layer, c uterine epithelium, d antimesometrial endometrium

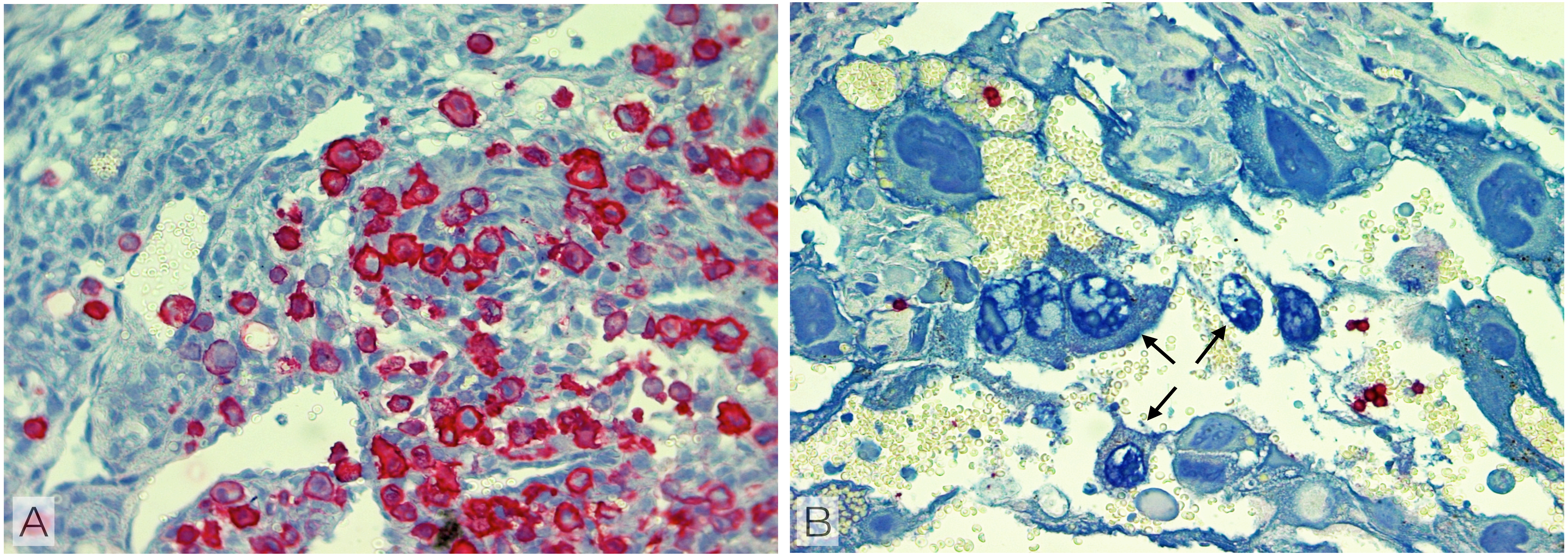

Slide 17: Upper insets of slide 15: B220 immunoreactivity in foam cells and small lymphocytes

A B220 positive foam cells in the decidua basalis. 40x. **B** Degenerating trophoblast cells (arrows); B220 positive small lymphocytes in maternal blood. 40x.

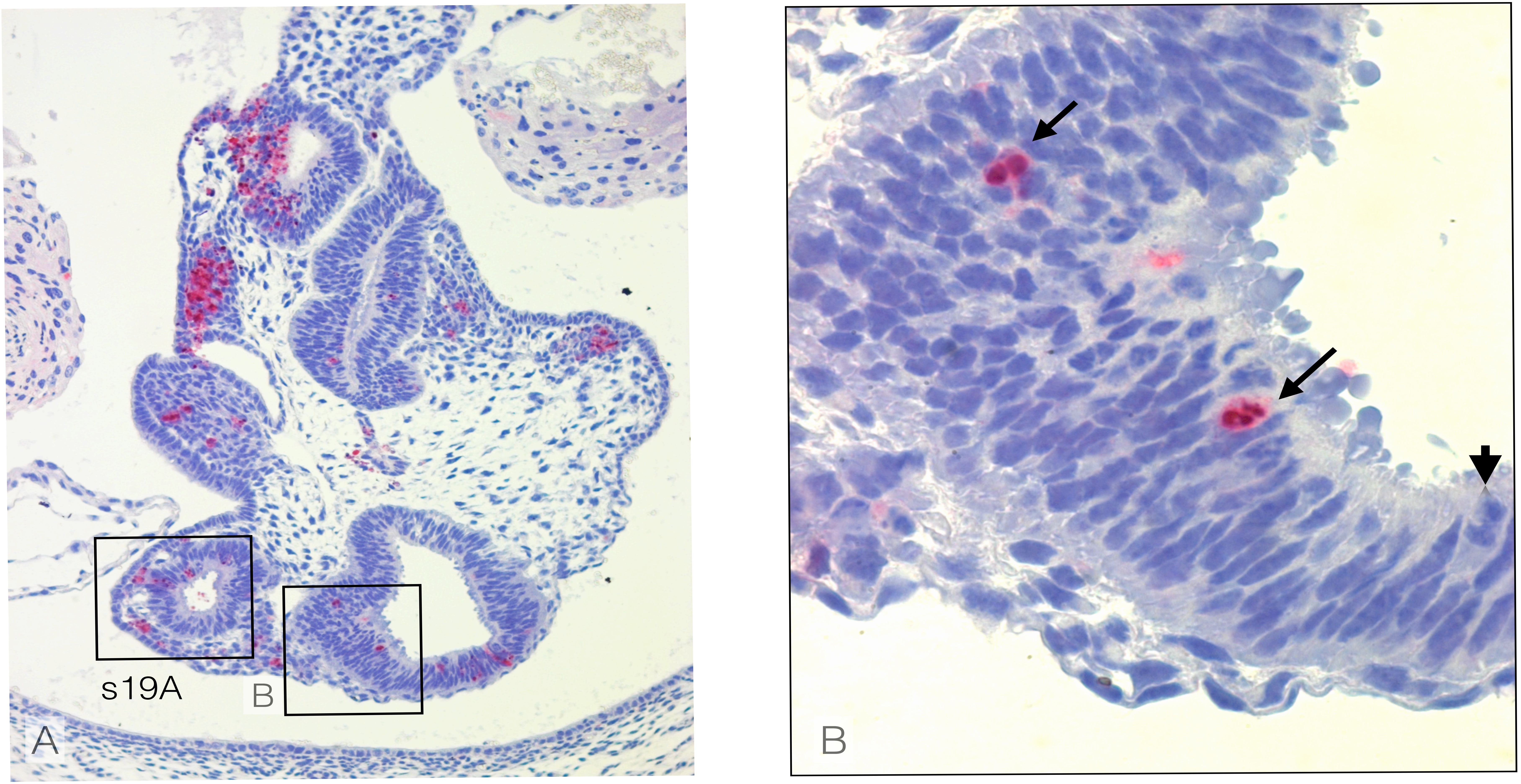

Slide 18: caspase 3 immunoreactivity in apoptotic embryo.

A Extruded embryonic head and neck of embryo (composite 4). Extended caspase 3 positive apoptotic areas in non physiological locations. 20x **B** Inset: Neuroepithelium with apical blebs and apoptotic mitotic figure (arrowhead), invaded by caspase 3 positive maternal neutrophils (arrows). 100x

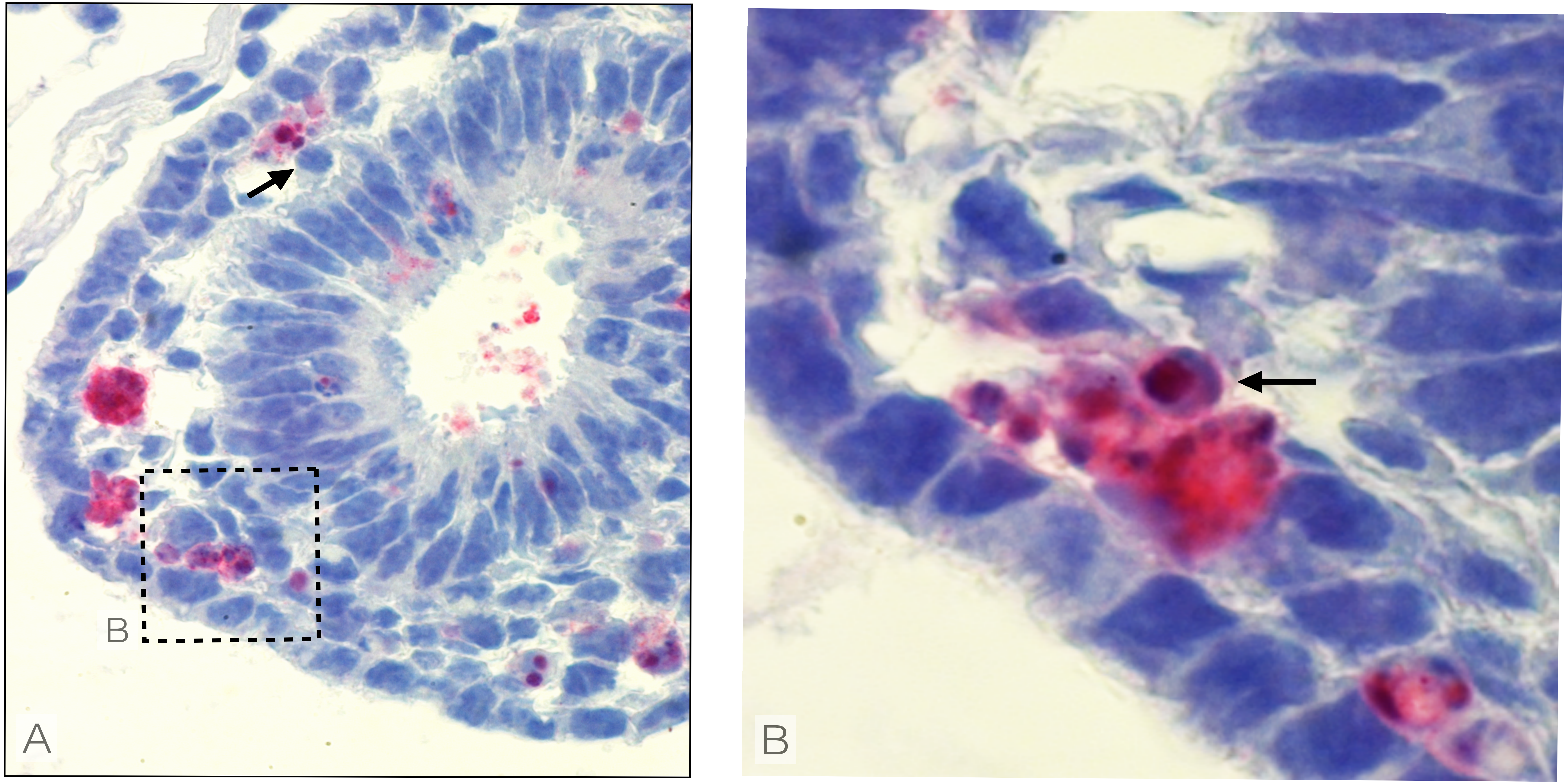

Slide 19: Inset A of Slide 18.

A Consecutive section of slice 13: caspase 3 negative (arrow) and putative positive embryonic blood cell 63x **B** Inset: Putative caspase 3 - positive embryonic blood cell (arrow). 100x

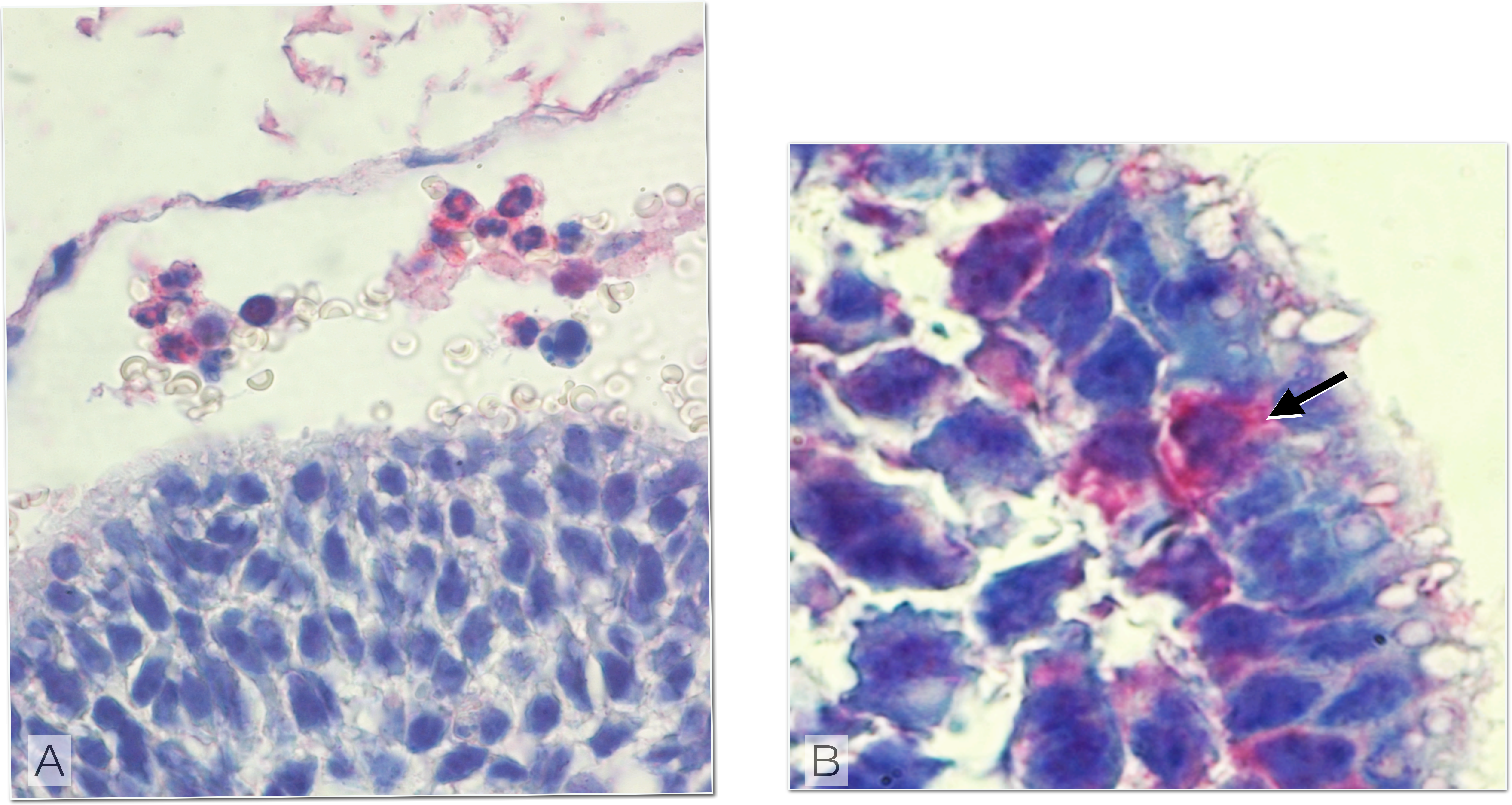

Slide 20: MPO positive maternal neutrophils in the apoptotic embryo

A MPO positive neutrophils in the amniotic cavity. 100x (for localisation compare Slide 15) **B** MPO positive neutrophils (arrow) in ectoderm of head anlage (for localisation compare Slide 21A). 100x with empty magnification

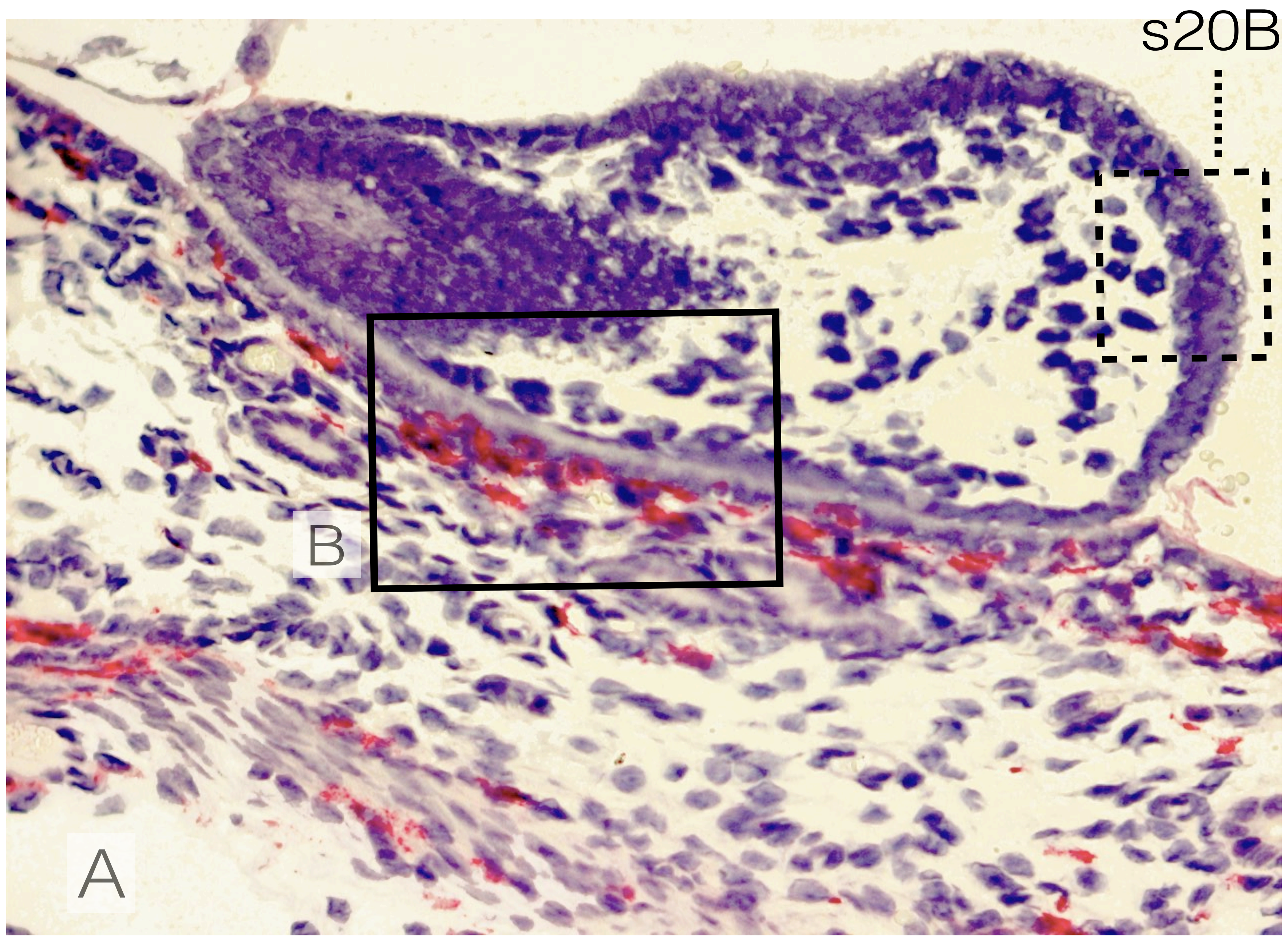

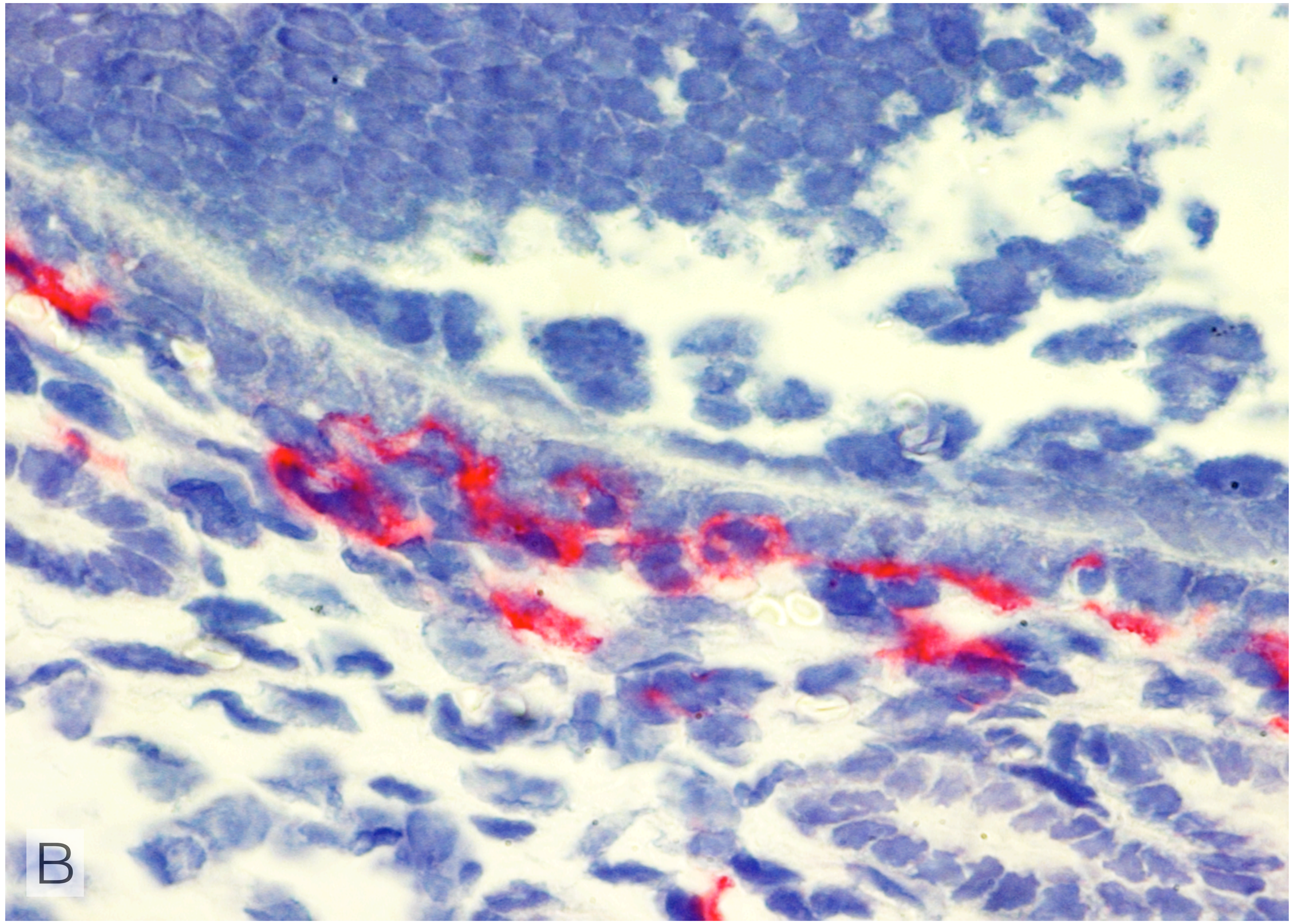

Slide 21: F4/80 positive maternal macrophages beneath uterine epithelium.

A Contact zone between uterine epithelium and extruded embryo. 20x **B** Inset: F80/4 positive maternal macrophages 100x

Composite 5

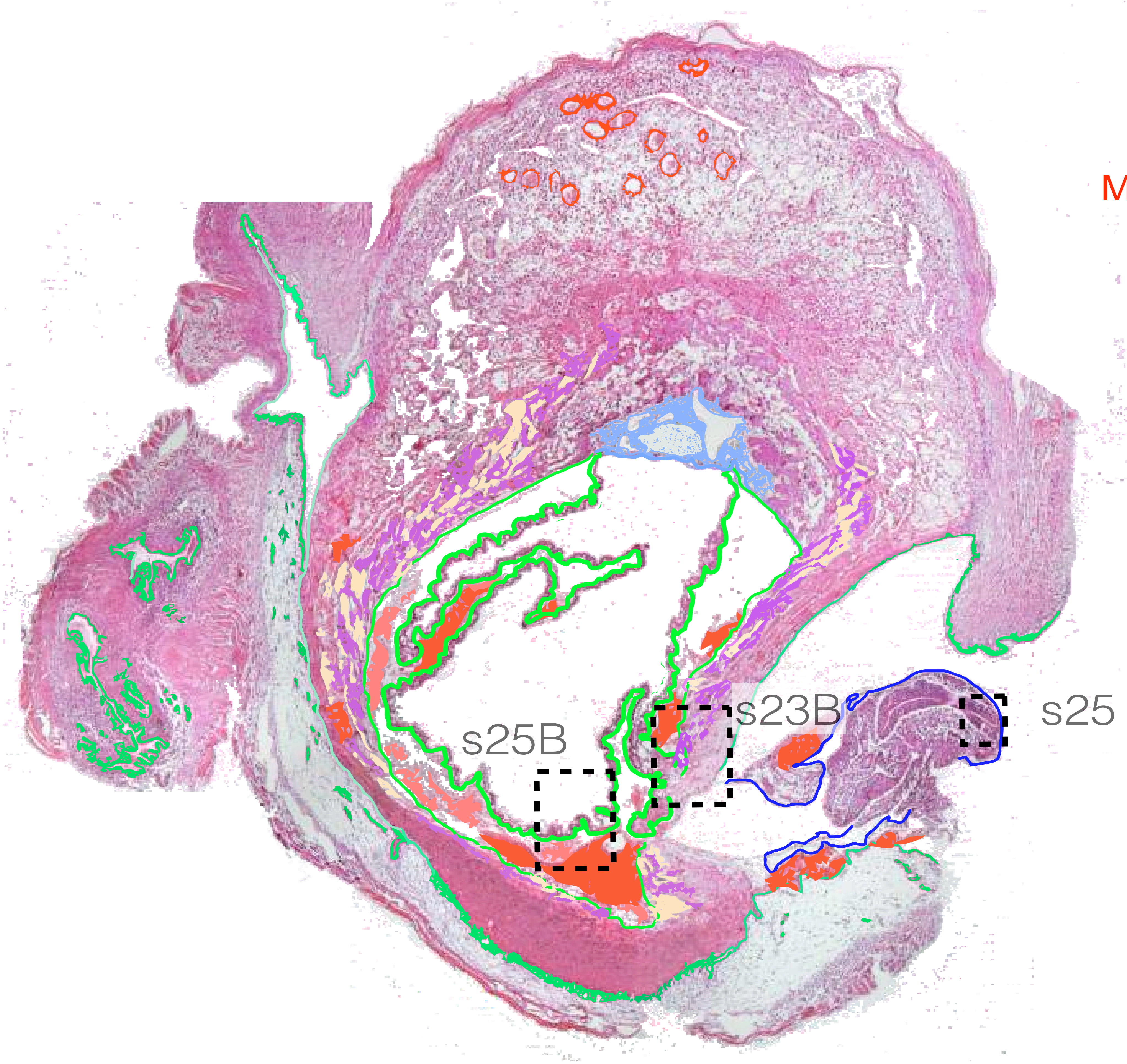

Slide 22 Composite 5 (R13): Resorption with totally aborted embryo and maternal haemorrhage

The embryo is completely expelled into the uterine lumen accompanied by massive maternal haemorrhage. Detection by US day 9, Histology day 9

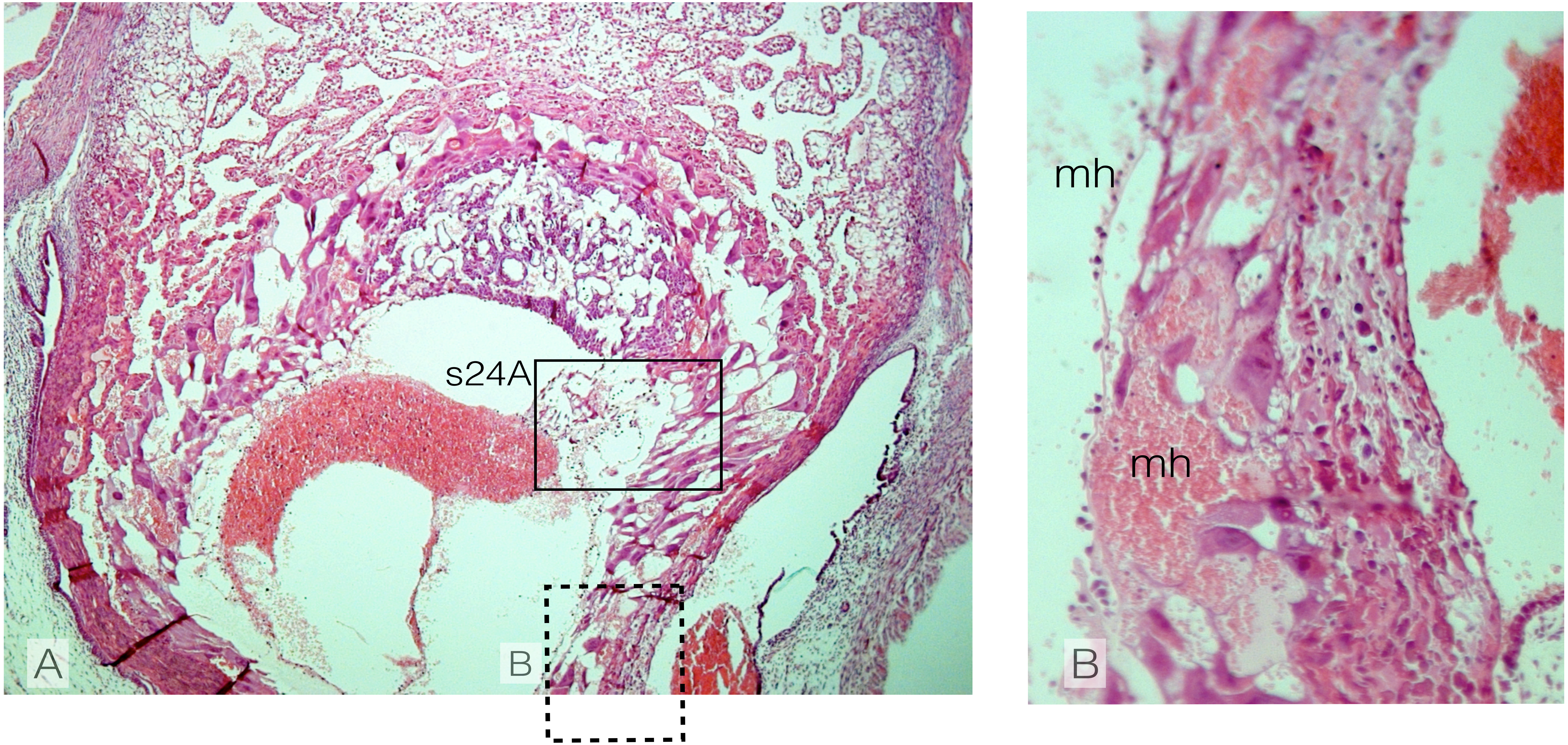

Slide 23: Delamination of embryonic and placental tissue by maternal haemorrhage

A Delamination zone formed by extended and distorted trophoblast lacunae (blue line) 5x HE **B** Inset (consecutive section ofg A): Decomposed decidua capsularis with haemorrhagic infiltration. Rm Reichert membrane, mh maternal haemorrhage 40x

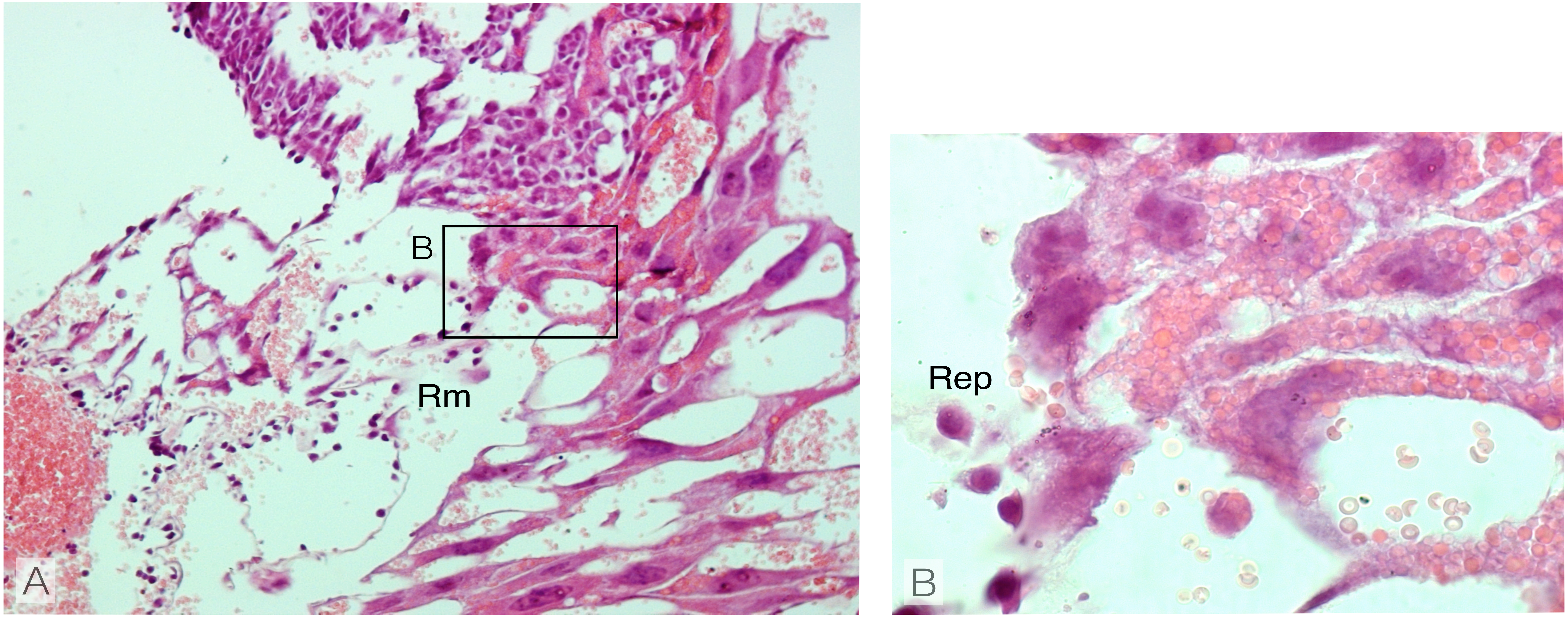

Slide 24: Insets of 23 A: Rupture zone of maternal and embryonic vessels

A Leaking of erythrocytes from maternal sinusoids into implantation chamber. The Reichert membrane (Rm) is ruptured 40x

B Inset: phagocytic trophoblast stuffed with vacuoles. Rep Reichert epithelial cells 100x

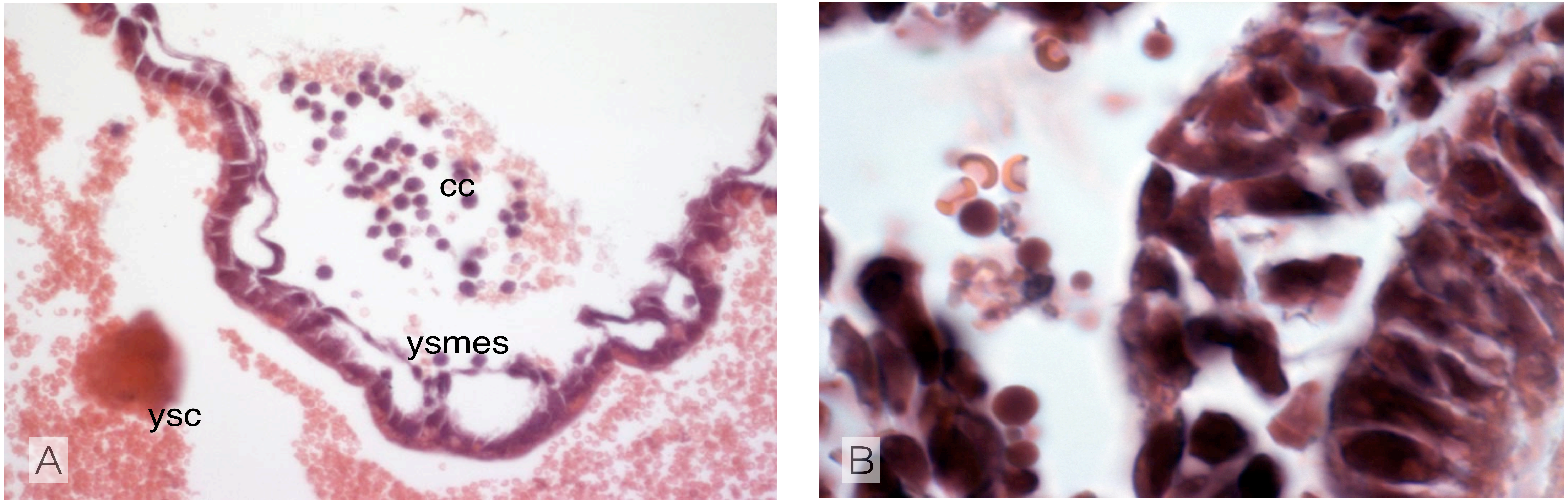

Slide 25 Mixed blood in chorionic cavity and in apoptotic embryo

A Mixed blood in chorionic cavity (cc) and maternal haemorrhage in yolk sac cavity (ysc). ysmes yolk sac mesoderm HE 20x

B Maternal erythrocytes within the aborted apoptotic embryo (for localization compare Slide 22) HE 100x

Composite 6

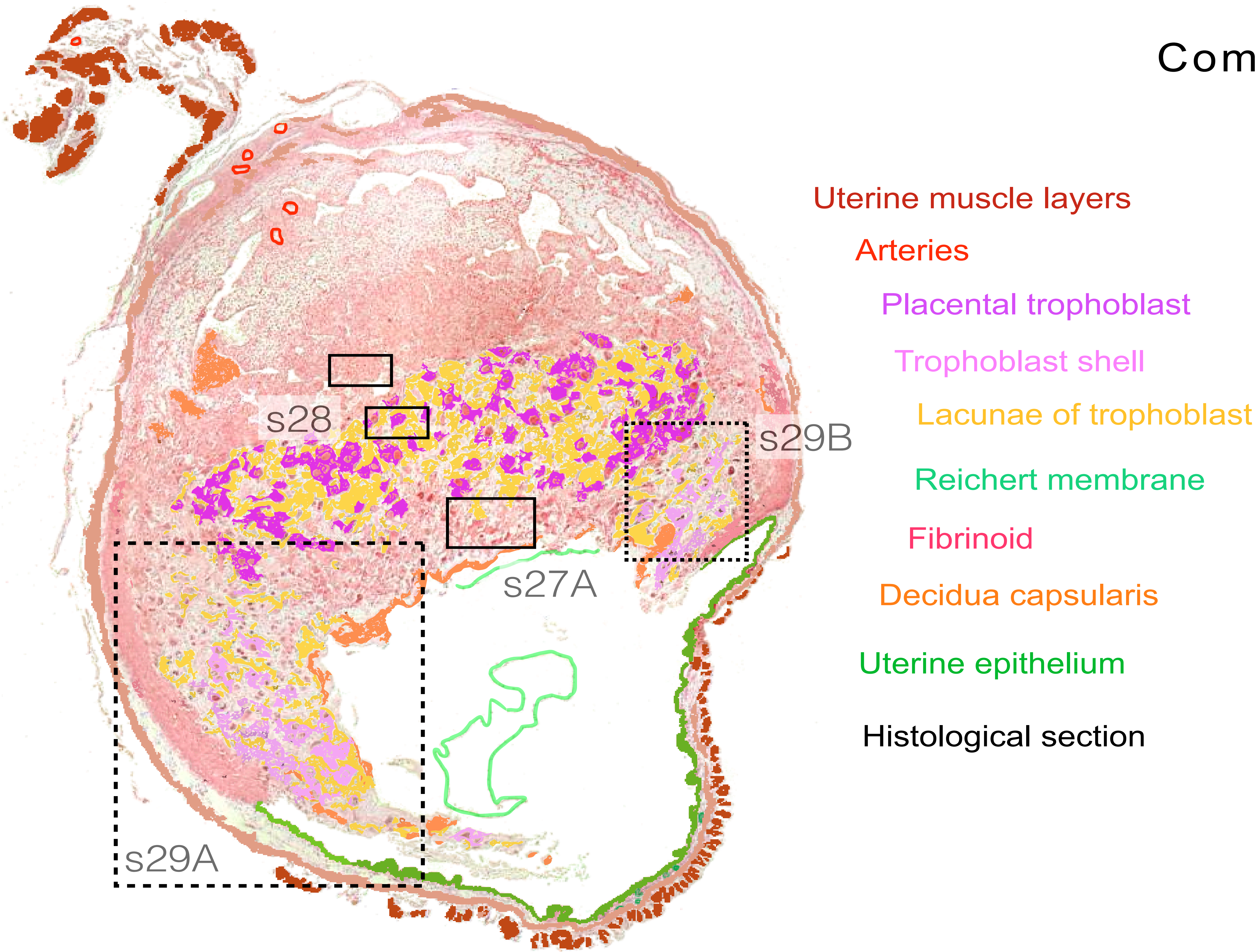

Slide 26 Composite 6 (R16) The final “empty cup” stage of resorption

The self-organizing placental trophoblast with lacunar giant cell character escapes degeneration. Remnants of degenerating outer lacunar trophoblast are attacked by maternal lymphocytes and neutrophils. Detection by US day 9, histology day 11

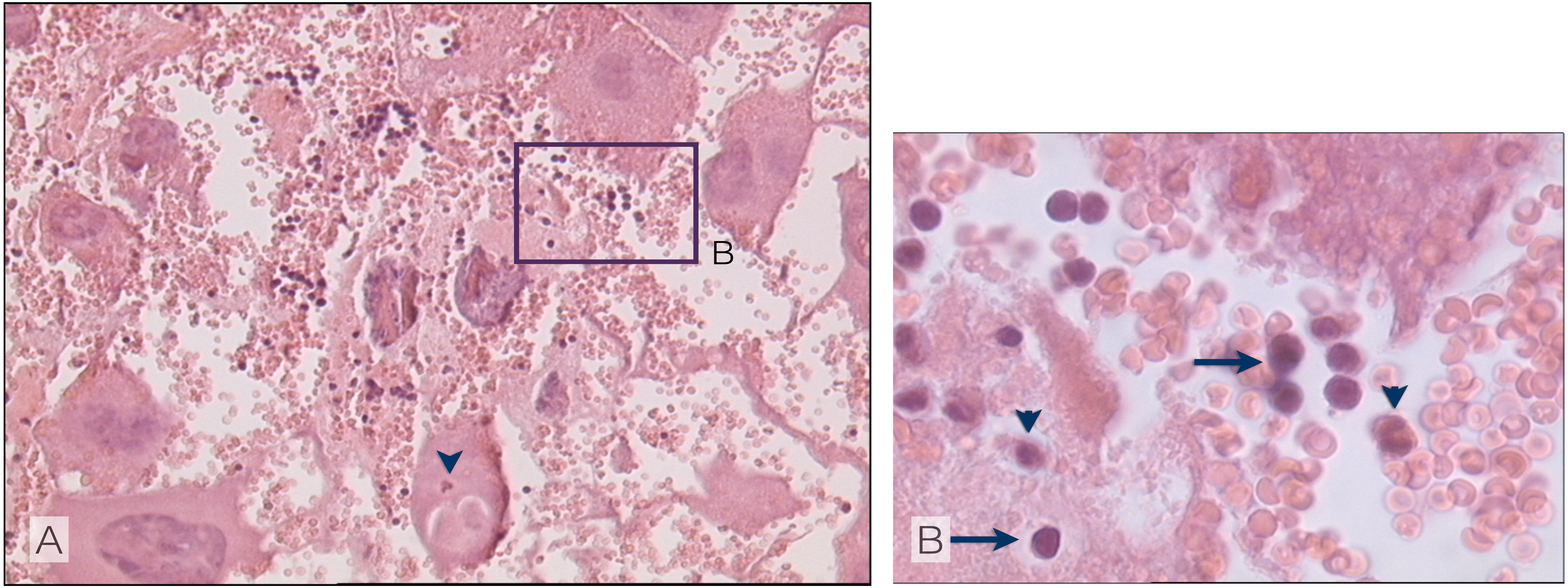

Slide 27: Maternal lymphocytes and neutrophils in the degenerated lacunar trophoblast

A Decaying trophoblast network. Neutrophil (arrow head) within trophoblast cell. HE 63x **B** Inset: lymphocytes (arrowhead) and neutrophils (arrows) invading decomposed trophoblast cells 100x

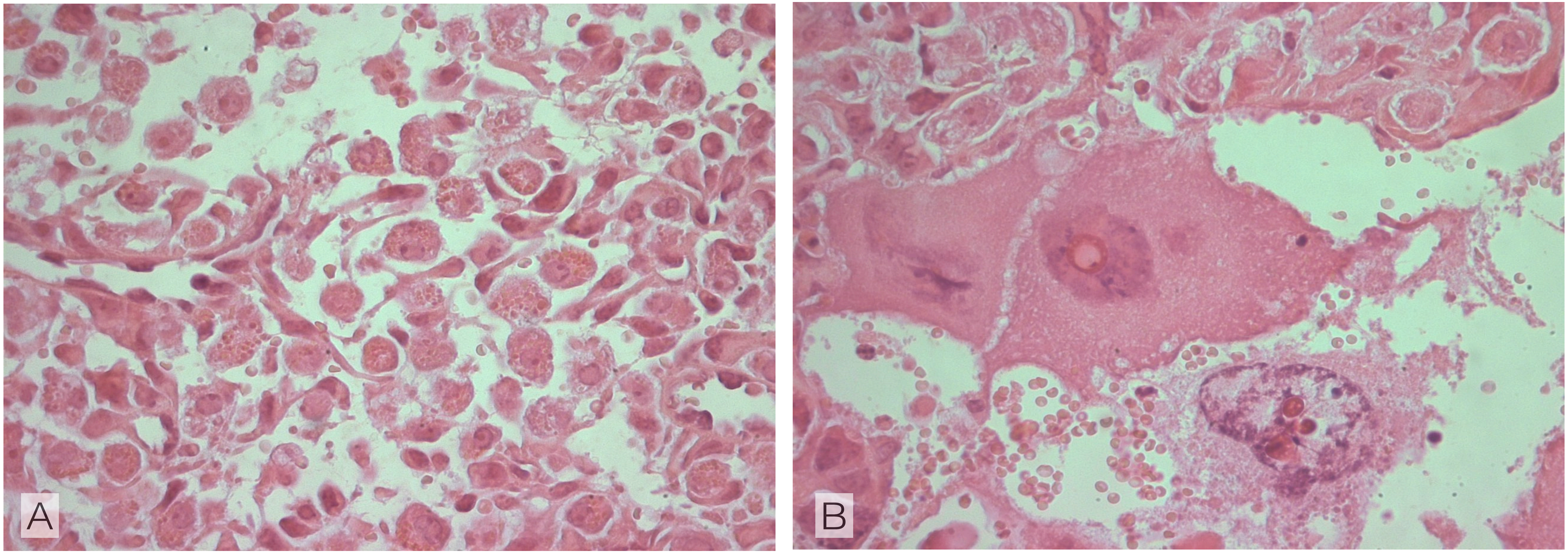

28

Slide 28: Regression of decidua basalis and trophoblast in the placental area

A Foam cells in the decomposing decidua basalis. HE 40x **B** Apoptotic placental trophoblast with pyknotic nucleus. HE 63x

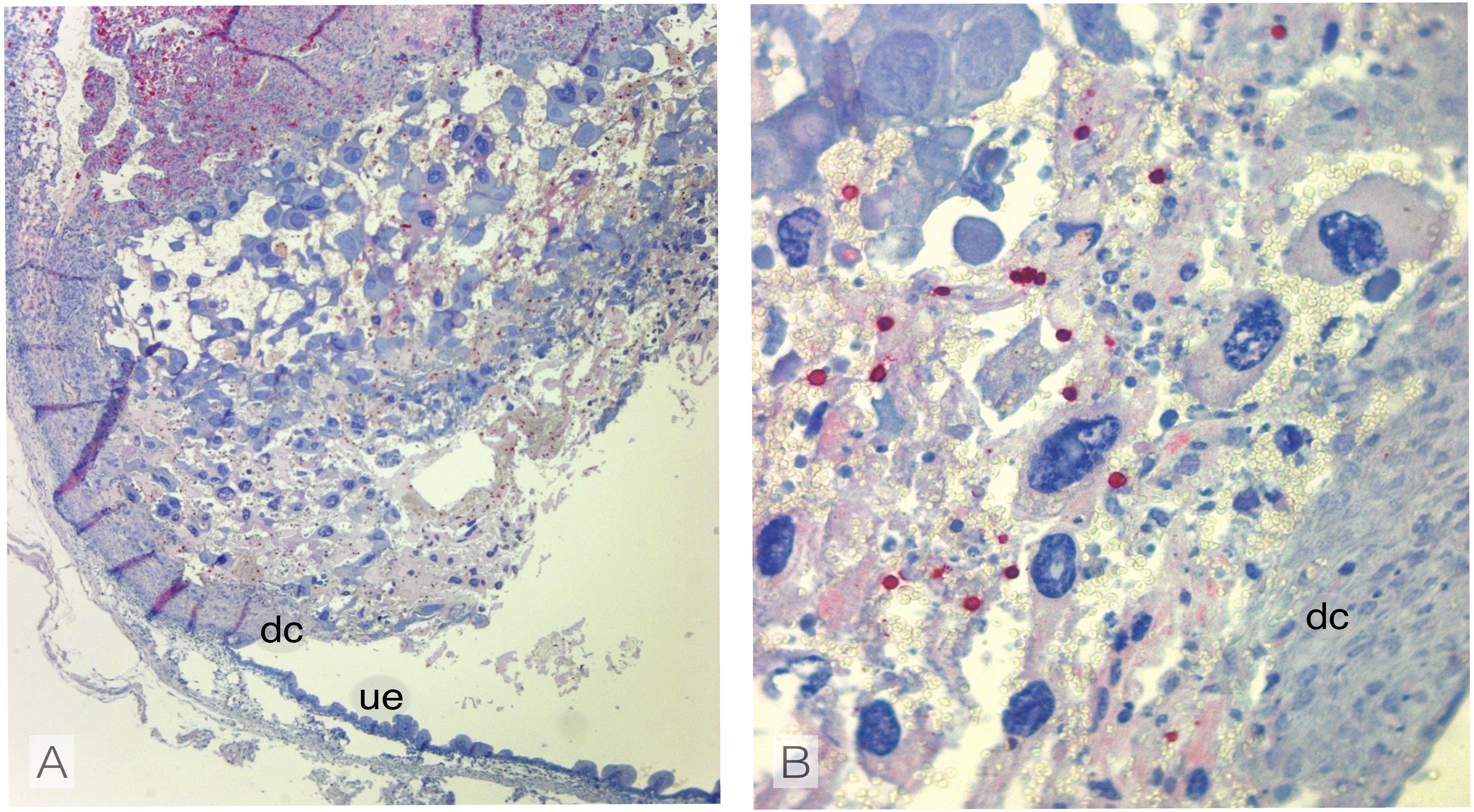

Slide 29: B220 Immunoreactivity in the “final cup” stage

A Denuded open wound area between disintegrating trophoblast area and uterine lumen. Villus-like extrusions in the high columnar uterine epithelium. For B220 immunoreactivity of foam cells in the compact zone of the decidua basalis compare Slide 15 and 17A, additional file 1. dc decidua capsularis, ue uterine epithelium. 5x **B** Removal of trophoblast remnants in the course of sterile inflammation. B220 negative neutrophils and B220 positive small lymphocytes 40x.

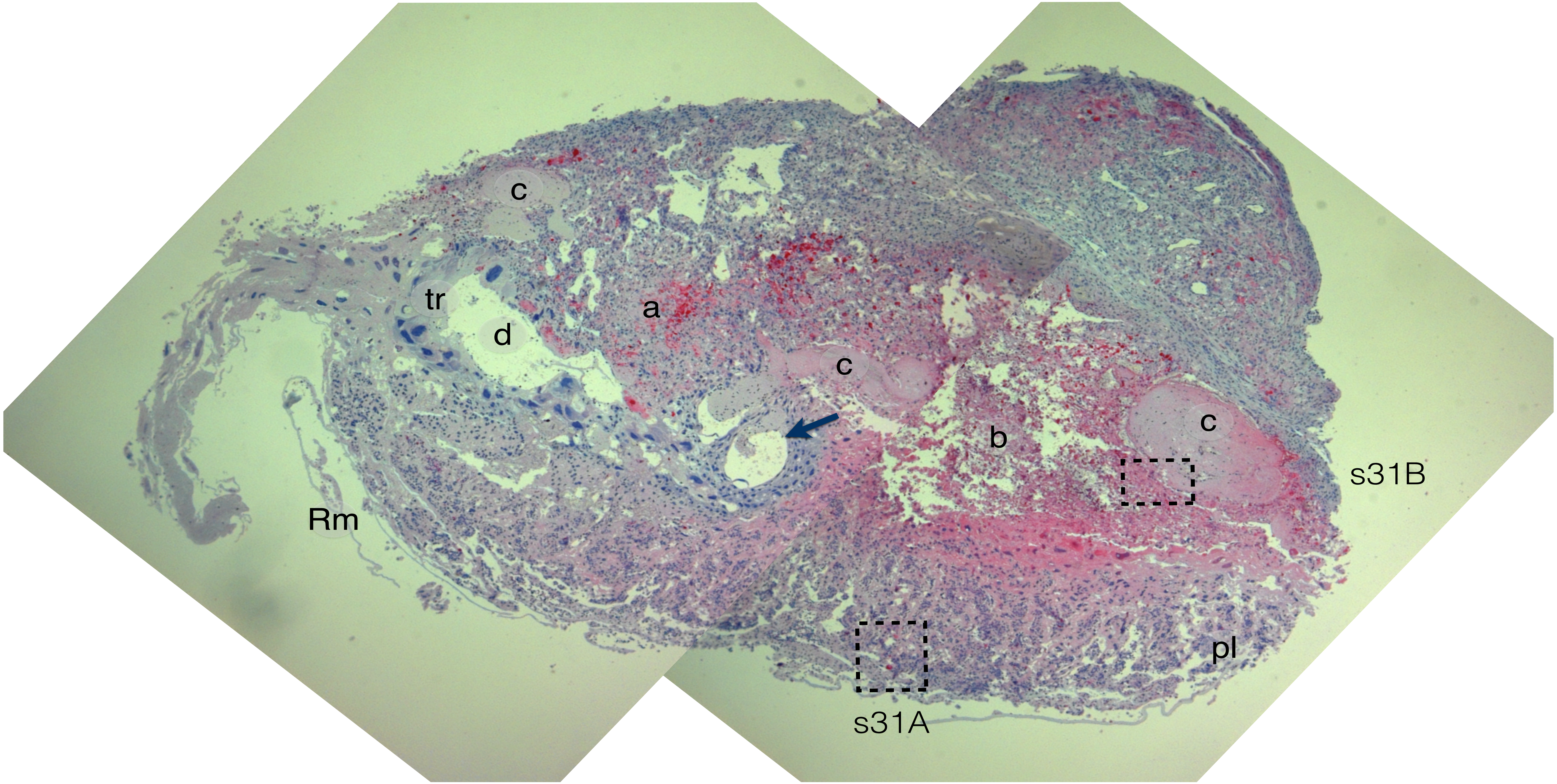

Slide 30: Overview of decaying placenta with caspase 3 immunoreactivity (R21)

Caspase 3 positive array. b zone of disintegration, c purulent foci, d empty space, tr trophoblast cells, pl placenta, Rm Reichert membrane. A purulent focus empties into the central spiral artery (arrow). 5x.

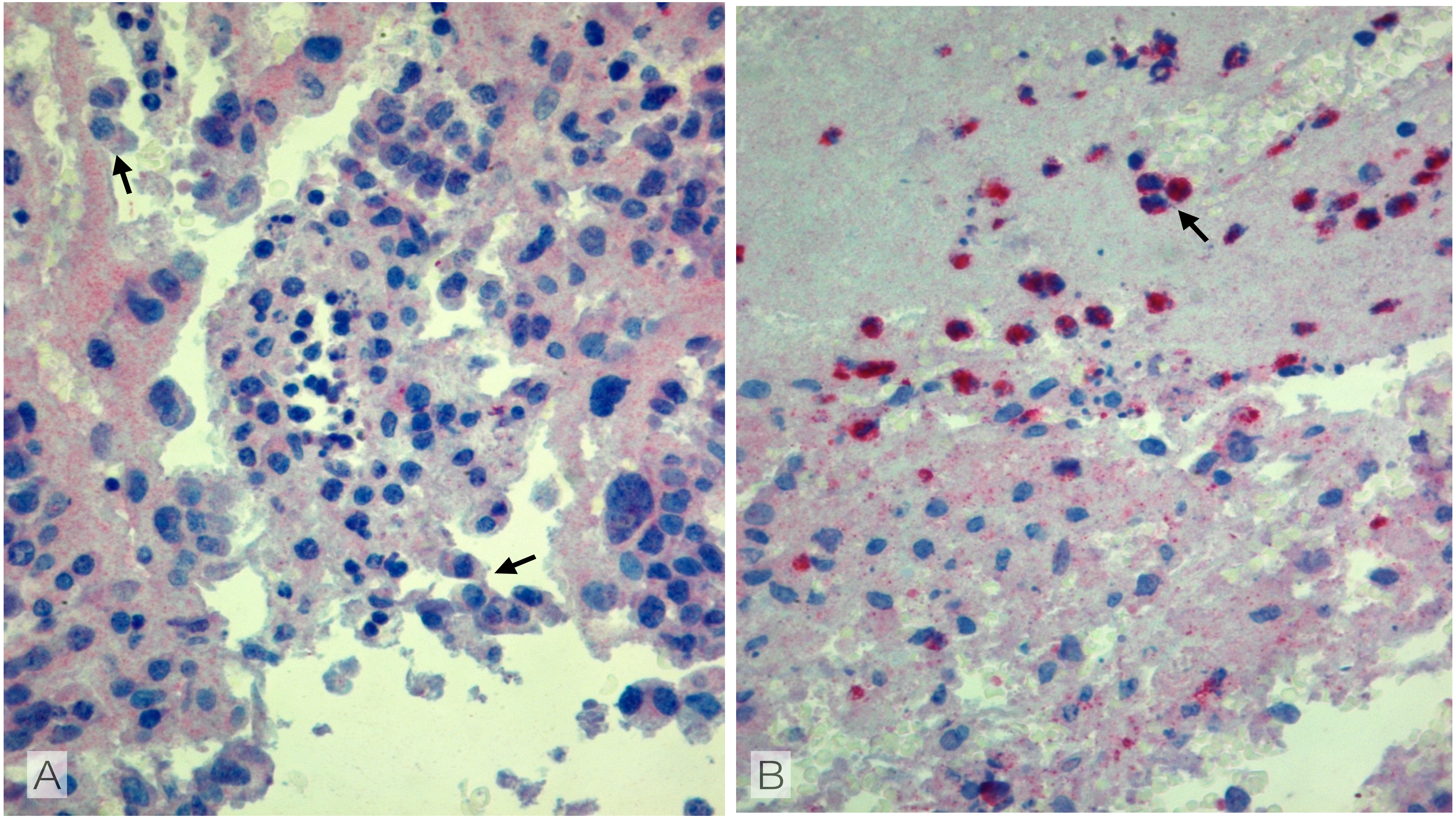

Slide 31: MPO immunoreactivity in placental and decidual areas (R21)

A Placental area with embryonic immune cells (arrows). 63x **B** Purulent focus in decidal area with MPO positive maternal neutrophils (arrow). 63x

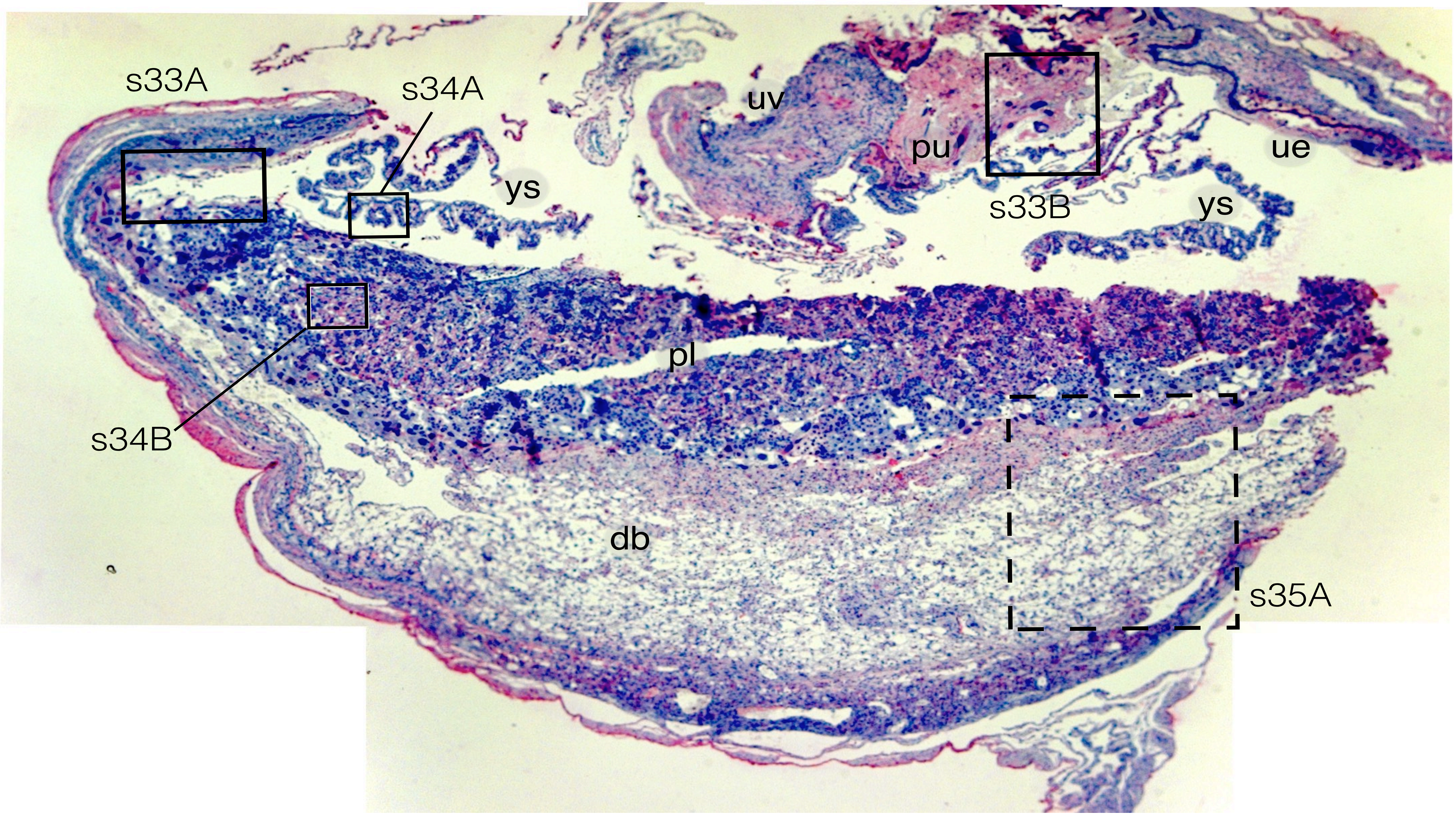

Slide 32: Overview of placenta with embryonic remnants, MPO immunoreactivity (R22)

Relatively well preserved yolk sac placenta, purulent embryonic umbilical area, and dissolution between placenta and basal decidua. db decidua basalis, pl placenta, ys yolk sac, uv umbilical vessels, pu purulent focus, ue uterine epithelium 5x.

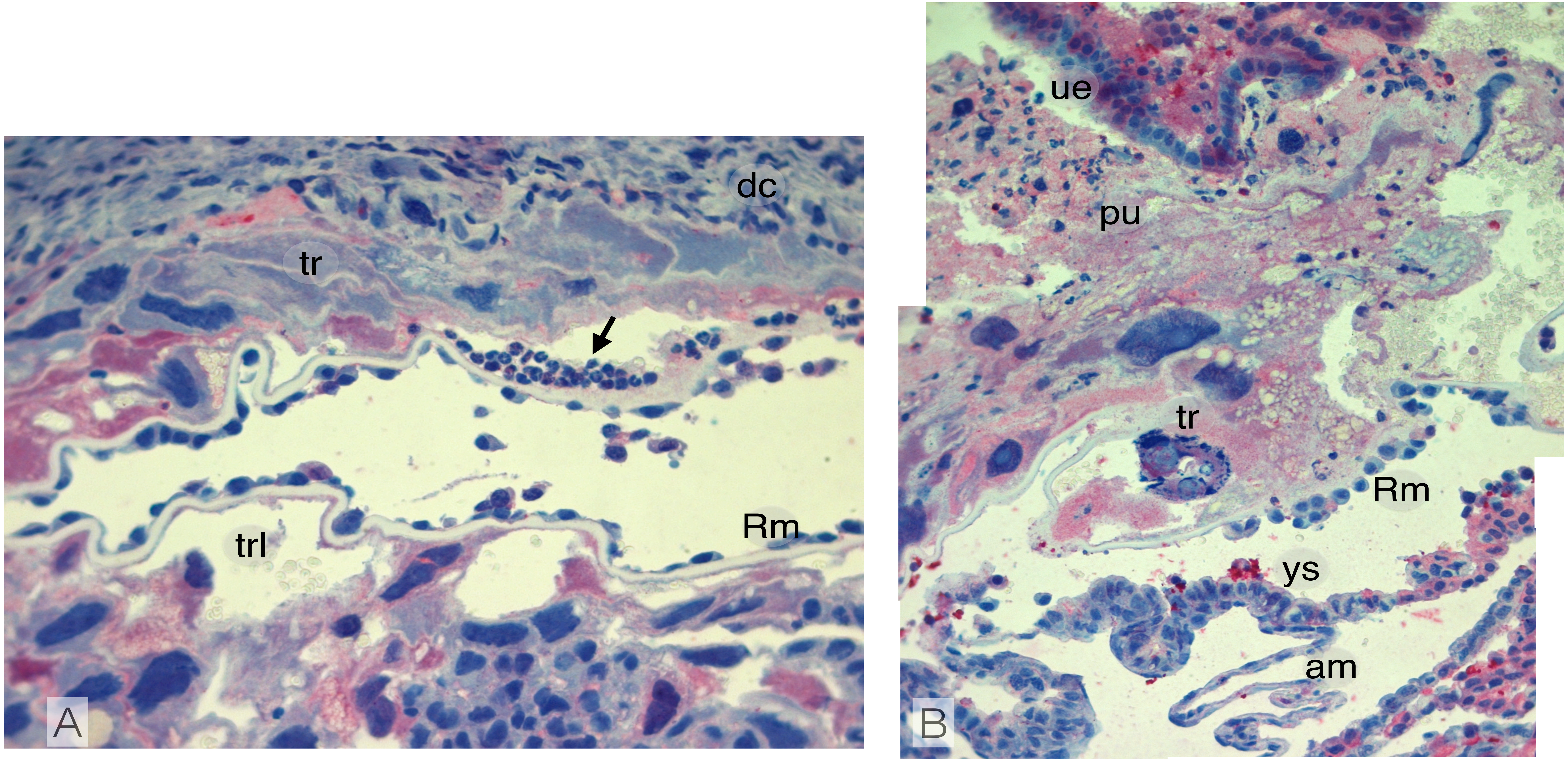

Slide 33: Insets of slide 32 (R22)

A Maternal neutrophils in the yolk sac placenta (arrow). Rm Reichert membrane with Reichert epithelial cells, tr lacunar trophoblast, trl trophoblast lacunae 40x **B** Purulent material (pu) at the site of the former embryo. Rm Reichert membrane, tr degenerated trophoblast, ys yolk sac epithelium, am amnion, ue uterine epithelium. 40x

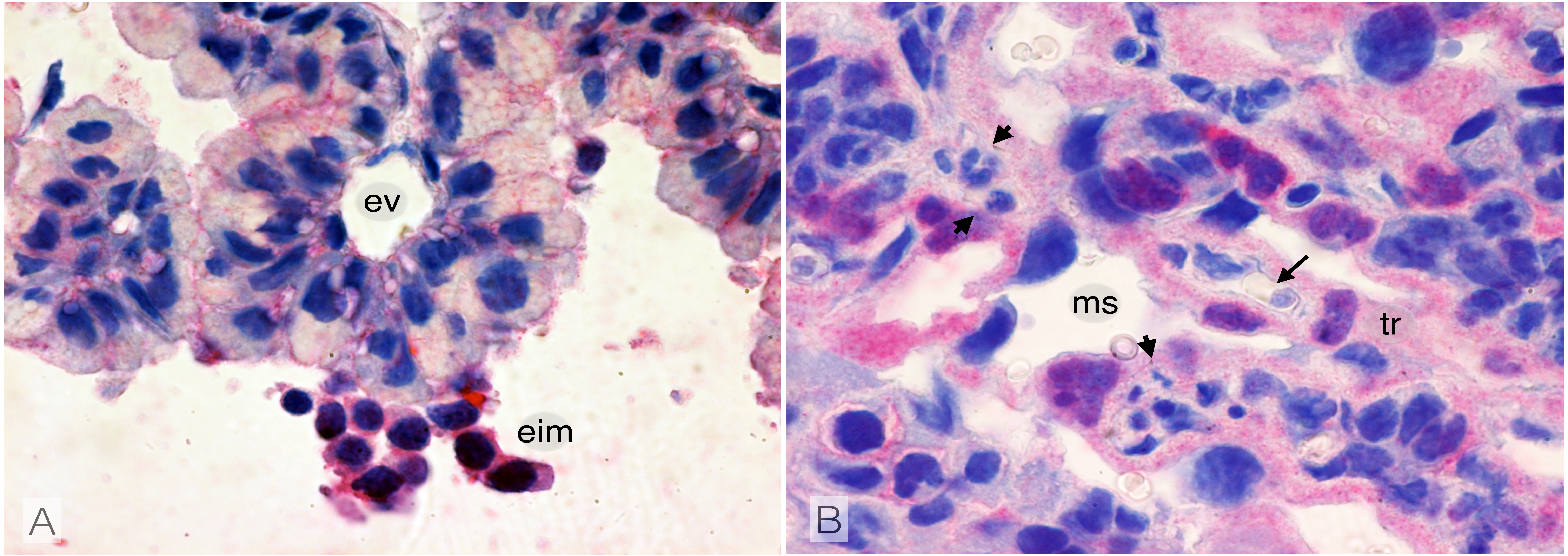

Slide 34: Yolk sac epithelium and labyrinth placenta (R22)

A Yolk sac villus with embryonic vessel (ev) and putative adhering embryonic immune cells (eim). MPO. 100x **B** Labyrinth placenta. ms maternal sinusoid with maternal erythrocytes, tr trophoblast layer with MPO positive granules, maternal neutrophils (arrowheads), embryonic erythrocyte (arrow) in embryonic vessel. 100x

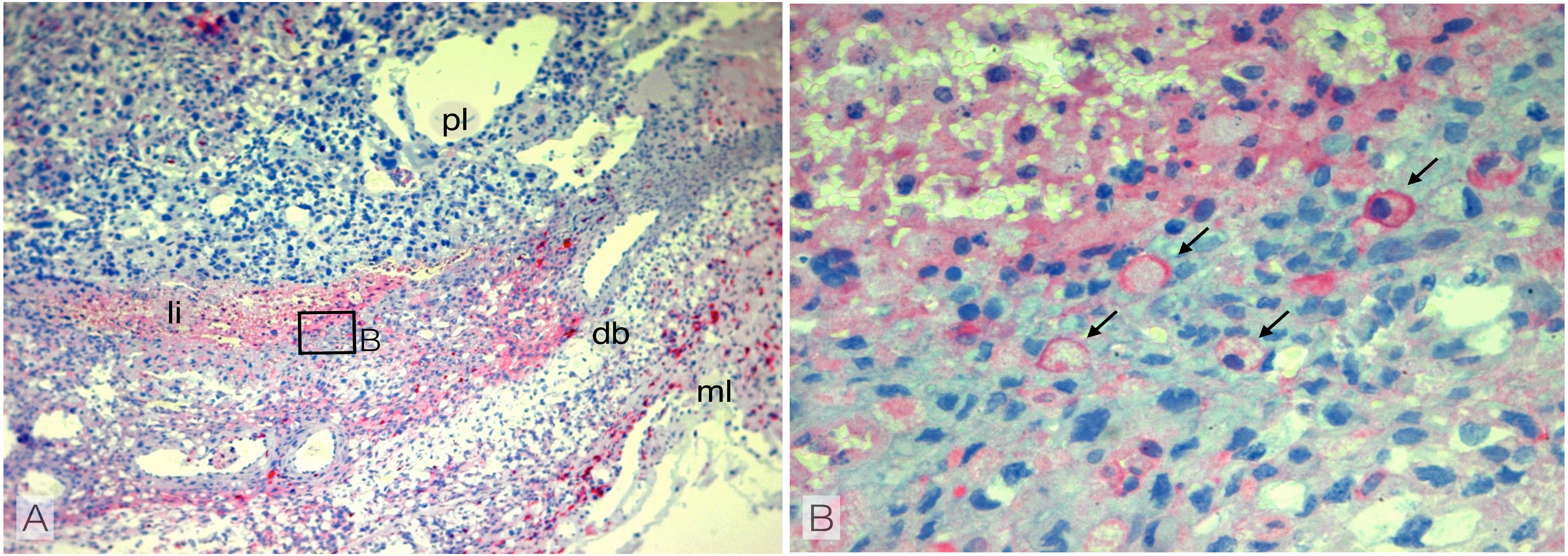

Slide 35: F4/80 immunoreactivity in macrophages and foam cells (R22)

A Area of liquefaction (li) between placenta (pl) and decidua (db). F4/80 positive macrophages in uterine muscle layer (ml). 10x **B** F4/80 positive foam cells (arrows) in the marginal zone. Decaying decidua basalis insinuated with maternal erythrocytes. 40x
