# Supplementary material for: Spontaneous embryo resorption in the mouse is triggered by embryonic apoptosis followed by rapid removal via maternal sterile purulent inflammation
Source: BMC Dev Biol. 2020 Jan 9;20:1. doi: 10.1186/s12861-019-0201-0 (PMC6953269; doi:10.1186/s12861-019-0201-0)

Composite 1

Interactive version
open side bar and check
layers

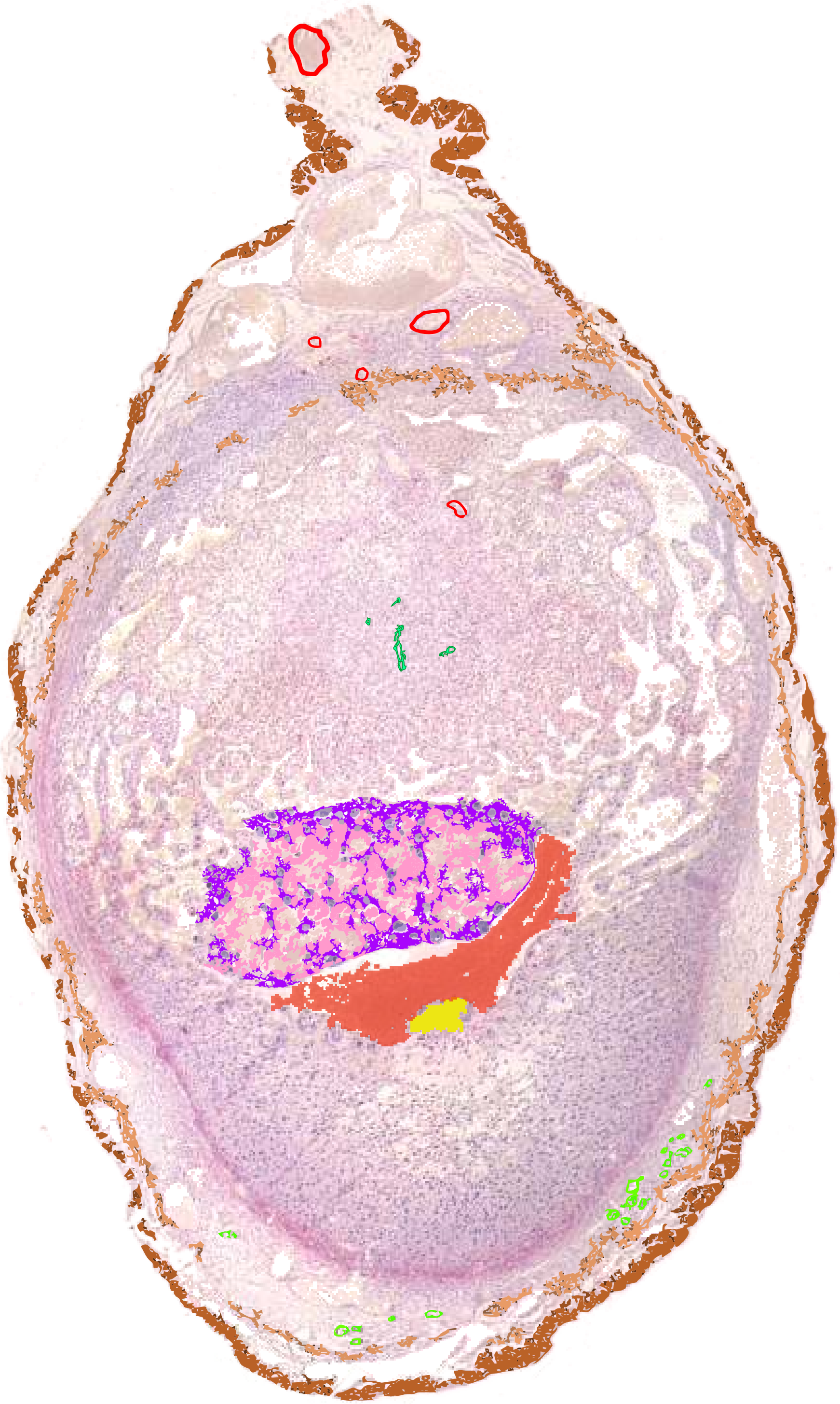

Uterine muscle layers

Arteries

Primary uterine lumen

Trophoblast array

Maternal haemorrhage

Purulent focus

Endometrial glands

Histological section

Composite 2

Interactive version
open side bar and check layers

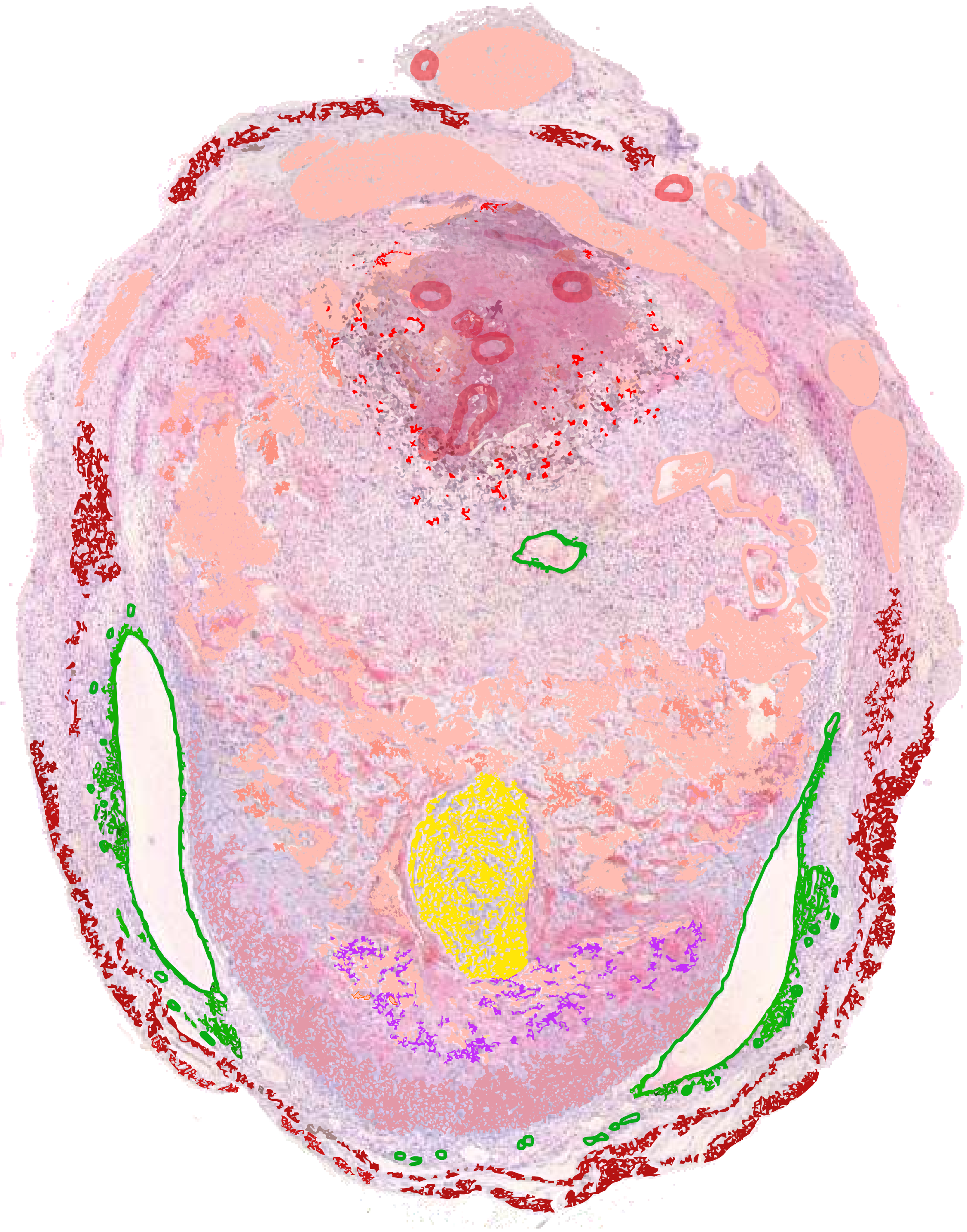

Uterine muscle layers

Arteries

Congested sinusoids

Liquified decidua basalis

Uterine epithelium

Purulent focus

Trophoblast

Decidua capsularis

Histological section

Composite 3

Interactive version
open side bar and check layers

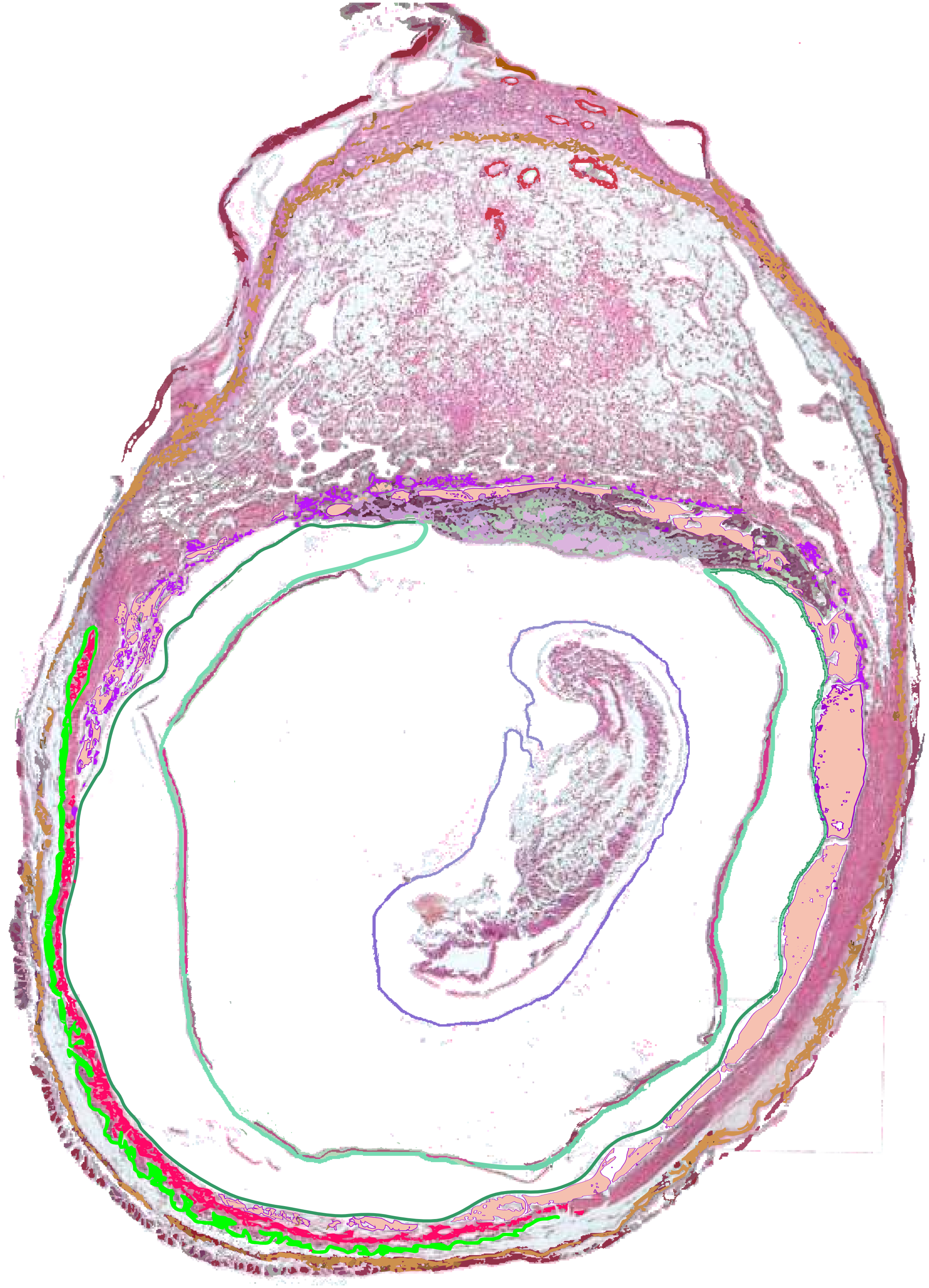

Uterine muscle layers

Arteries

Trophoblast with lacunae

Allantois mesoderm

Amnion

Yolk sac with Reichert membrane

Fibrinoid

Uterine epithelium

Histological section

Composite 4

Interactive version
open side bar and check layers

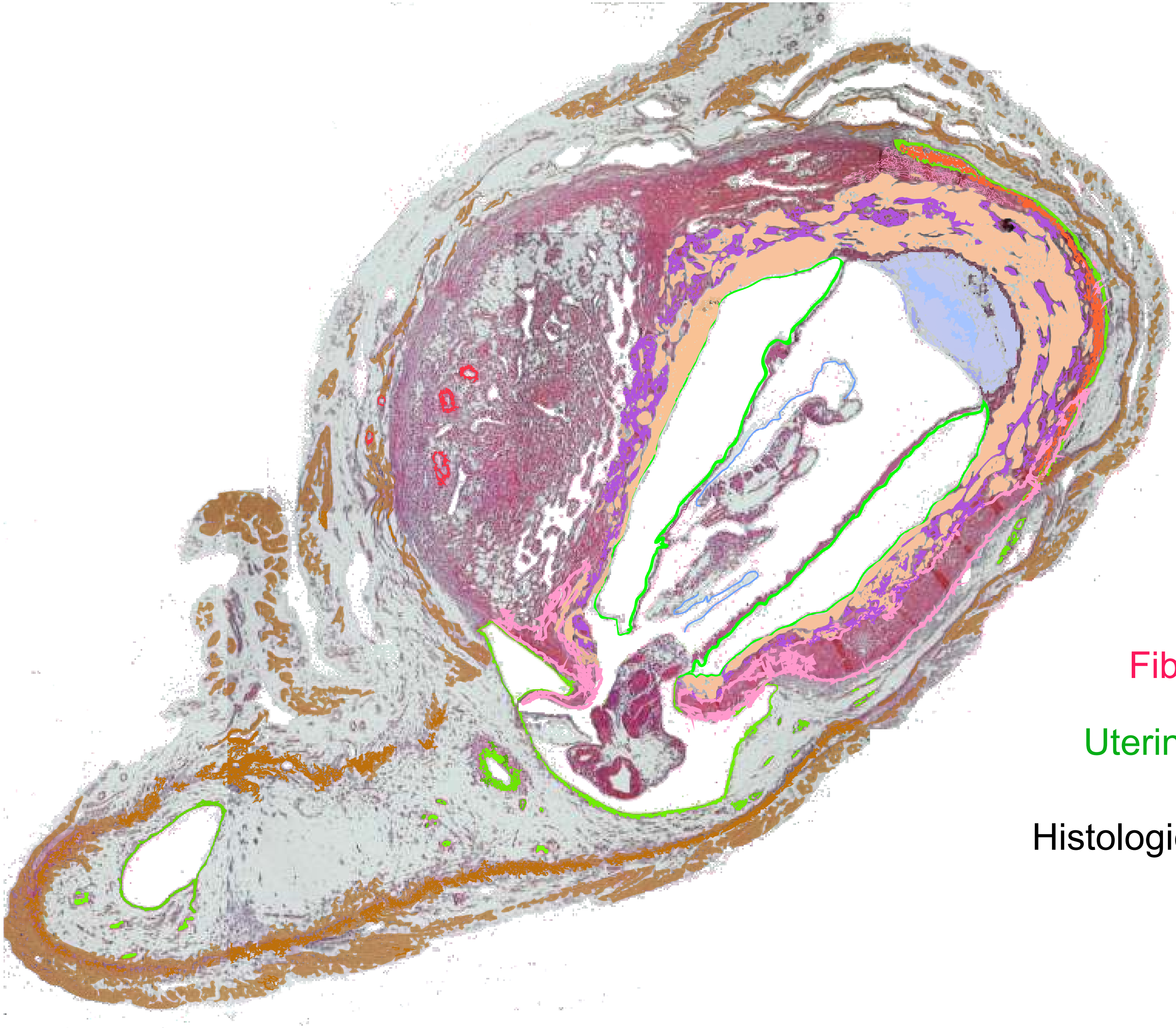

Uterine muscle layers

Arteries

Lacunar trophoblast

Allantois mesoderm

Yolk sac

Amnion

Decidua capsularis

Fibrinoid

Uterine epithelium

Histological section

Interactive version
open side bar and check layers

Composite 5

Interactive version
open side bar and check layers

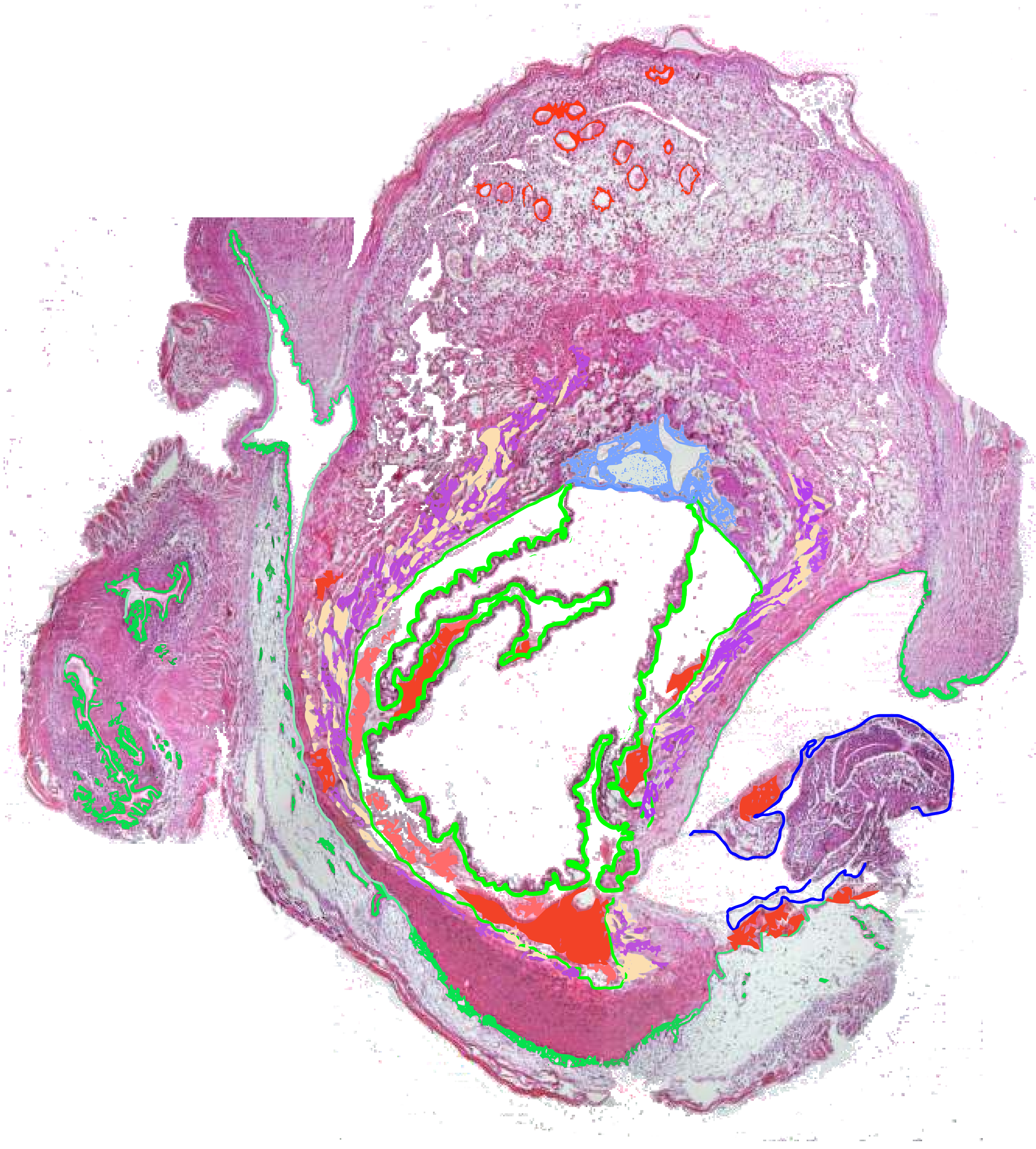

Maternal arteries

Lacunar trophoblast

Allantois mesoderm

Yolk sac

Clotting maternal blood

Uterine epithelium

Amnion

Histological section

Composite 6

Interactive version
open side bar and check layers

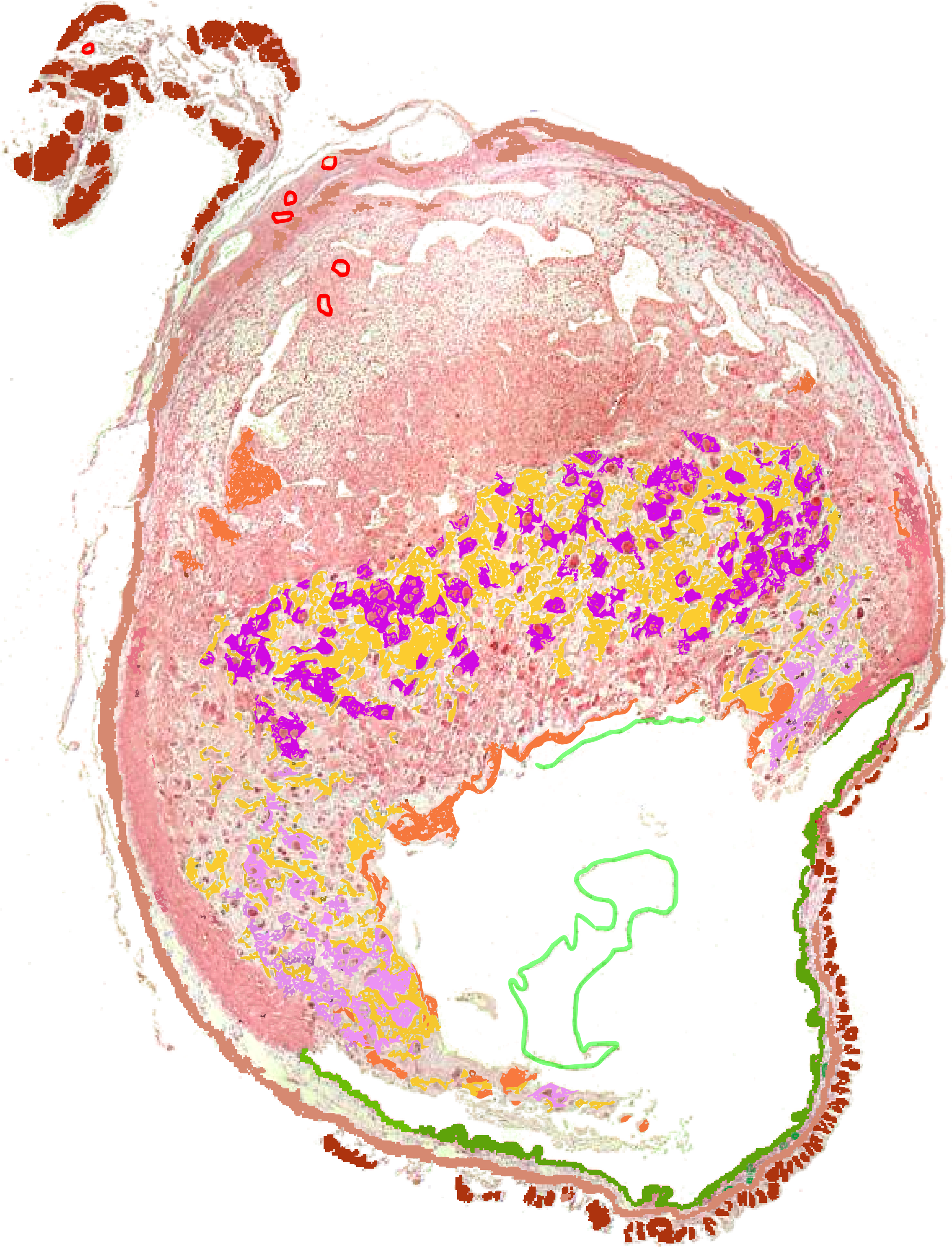

Uterine muscle layers

Arteries

Placental trophoblast

Trophoblast shell

Lacunae of trophoblast

Reichert membrane

Fibrinoid

Decidua capsularis

Uterine epithelium

Histological section

Day 6 normal embryo

Interactive version
open side bar and check layers

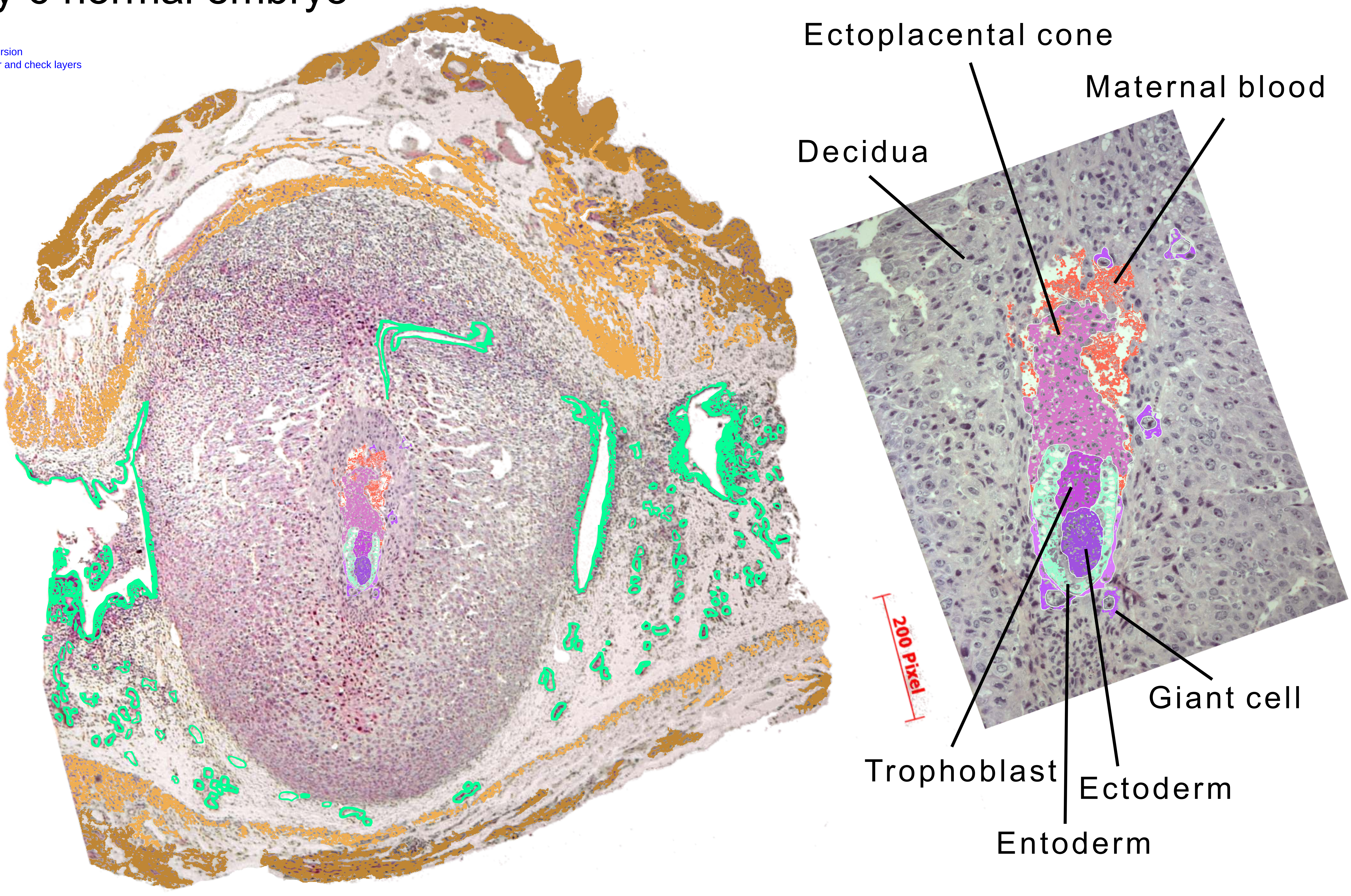

Day 9 normal embryo

Interactive version
open side bar
and check layers

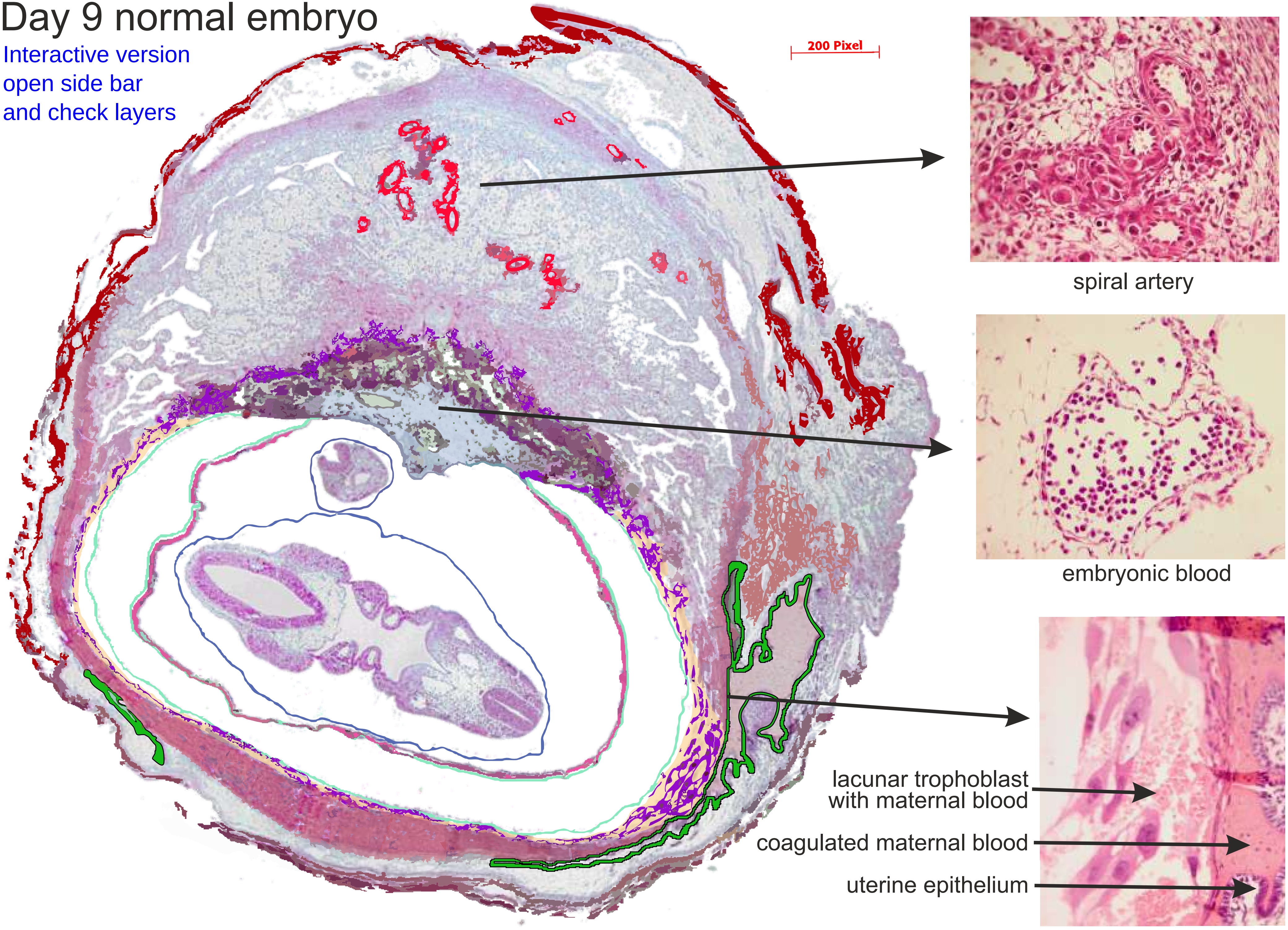

Supplement: Supplementary file 2 — Additional file 2. Interactive composites. [file 12861_2019_201_MOESM2_ESM.pdf]
